# Diversity in Zwitterionic Metal Ammonium Tris(phenolate)s for the Controlled Immortal Polymerization of Lactide: Dramatic Activity Enhancement and Mechanistic Insight on Expansion beyond Zirconium

**DOI:** 10.1021/acscatal.5c01857

**Published:** 2025-05-14

**Authors:** Matthew G. Davidson, Catherine J. Frankis, Matthew D. Jones, Gabriele Kociok-Köhn, Frank Marken, Strachan N. McCormick, James Tipler, Philip B. Yang

**Affiliations:** † Institute of Sustainability and Climate Change, 1555 University of Bath, Bath BA2 7AY, U.K.; ‡ Department of Chemistry, 1555 University of Bath, Bath BA2 7AY, U.K.; § Core Research Facilities (CRF), 1555 University of Bath, Bath BA2 7AY, U.K.

**Keywords:** ring-opening polymerization, rare earth, lanthanide, coordination geometry, redox switch, lactide

## Abstract

Reaction of tris­(2,4-dimethylbenzyl)­amine, H_3_L^Me^, with tris- or tetrakis­(alkoxide)­s of large metals consistently affords, respectively, *pseudo*-homoleptic and homoleptic zwitterionic compounds [M­(III)­(HL^Me^)­(H_2_L^Me^)] (M = Yb­(III), Y­(III), Pr­(III), La­(III), Sc­(III), Sm­(III)) and [M­(IV)­(HL^Me^)_2_] (M = Zr­(IV), Hf­(IV), Ce­(IV)). The Zr­(IV) congener is known to be a robust and efficient catalyst for the ring-opening polymerization of lactides under industrially relevant solvent-free conditions, exhibiting some heteroselectivity in the polymerization of the racemic monomer. The generality of the synthetic route, encompassing various metals, permits exploration of the role of metal center size and other subtle structural variations in influencing catalytic activity and selectivity. Kinetic studies have revealed all M­(III) compounds assessed (M = Yb­(III), Y­(III), Pr­(III), La­(III)) to be significantly more active than the Zr­(IV) system, exhibiting a clear correlation between ionic radius and reaction rate, while generally retaining a high degree of control. The La­(III) compound, in particular, offers both remarkable activity (>20 × Zr­(IV) at 120 °C, 50 wt %/vol monomer in PhCl) and resilience under challenging, industrially relevant conditions (180 °C, solvent-free, 2×10^–3^–5×10^–3^ mol % catalyst). Comprehensive structural analyses have, additionally, afforded insight into the unusual mechanism favored by these catalysts. Although only the Zr­(IV) and Hf­(IV) systems exhibit appreciable stereoselectivity, variable-temperature ^1^H NMR spectroscopic and crystallographic methods have illuminated trends regarding the conformational chirality of the ligand systems in the compounds of interest, the facility of inversion of which we propose underpins much of the variation in their catalytic properties. Additionally, whereas the Ce­(IV) system, despite its greater metal size, did not tend to outperform Zr­(IV), in situ reduction to the anionic [Ce­(III)­(HL^Me^)_2_]^−^ provided an activity enhancement assessed to exceed 2 orders of magnitude. Accordingly, Ce­(III) offers a similarly dramatic rate enhancement when benchmarked against Zr­(IV).

## Graphical abstract

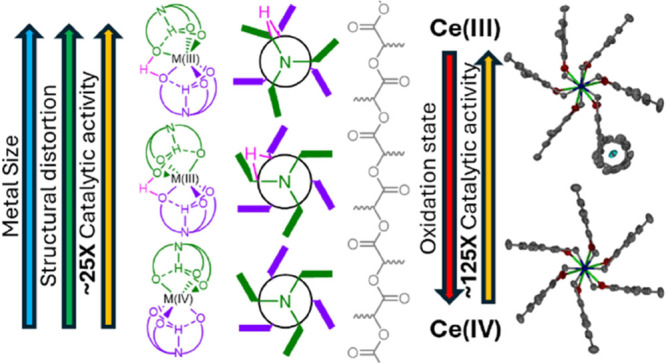

## Introduction

The provision of renewable, compostable plastics with properties sufficiently varied and tunable for comprehensive replacement of oil-derived materials is an enduring challenge for synthetic chemists. Moreover, the limited structural diversity of currently scalable biobased feedstocks, relative to petrochemical resources, necessitates alternative approaches to manipulating polymer properties, including controlled and switchable catalytic methods for the modification of polymer architecture via sequence control. Specifically, the stereoselective ring-opening polymerization (ROP) of racemic lactide (*rac*-LA) in the presence of metal-based catalysts, which, irrespective of stereoselectivity, typically proceeds via either a coordination–insertion^1–7^ or activated monomer mechanism,^2,5,8,9^ has been extensively studied. However, while the polymerization of lactides (Scheme 1) is already established on an industrial scale, this typically corresponds to the use of nonstereoselective catalyst systems for the ROP of a stereopure monomer feed (generally l-lactide, l-LA). Indeed, the development of robust, industrially relevant catalytic protocols with enhanced capabilities is reliant upon improved understanding of the mechanistic bases and structure–activity relationships upon which various systems’ polymerization activity and, in particular, selectivity are predicated.

**Scheme 1:**
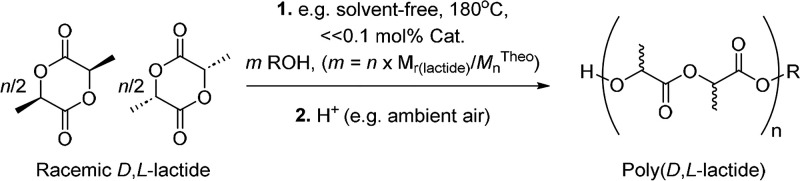
General Scheme for the Immortal Catalytic ROP of *rac*-LA under Industrially Relevant Conditions

Our group and others, most notably Kol and co-workers, have extensively studied the use of amine tris­(phenolate)-supported Group 4 metal-based initiators for the polymerization of lactides, including examples of highly active^10^ and industrially relevant^2^ protocols.^1,11–13^ All such compounds are characterized by conformational chirality of the ligand system (denoted ***P***/***M***, using notation established for helical systems) arising from directional (paddlewheel-like) arrangement of the phenolate groups comprising the *C*_*3*_-symmetric ligand framework. Of greatest relevance to the current work, we have recently reported a robust (air- and moisture-stable), highly active, and inexpensive catalyst, based around zirconium, a benign and earth-abundant metal, as a credible alternative to tin­(II) bis­(2-ethylhexanoate), Sn­(Oct)_2_, for the industrial ROP of lactides. We have also demonstrated its use for ROP on the kg-scale, and on application to the ROP of *rac*-LA under solvent-free conditions (174 °C), that protocol, employing homoleptic, zwitterionic Zr­(IV) ammonium tris­(phenolate) compound **1**, formed on reaction of [Zr­(O*^i^* Pr)_4_·(HO*^i^* Pr)] with pro-ligand tris­(2-hydroxy-3,5-dimethylbenzyl)­amine, afforded a heterotactically enriched product (*P*_r_ = 0.67).^2,14^ Such heteroselectivity has been attributed to the dynamic conformational chirality of the tris­(2-hydroxy-3,5-dimethylbenzyl)­amine-derived ligand framework.^2,11,12,15^

Having established the wide-ranging attraction of the Zr-based initiator system **1**, we became interested in the development of related protocols beyond this prototypical example, specifically to encompass alternative metal centers. The rare earth elements, encompassing yttrium, scandium and the lanthanide series are, like Zr, of interest in the context of industrial catalysis, due to their relatively high crustal abundance and low toxicity, as well, in the case of the lanthanides, as their large ionic radii and flexible coordination geometry, governed principally by steric constraints, with bonding interactions being largely nondirectional and ionic in nature.^16–23^ Additionally, the chemistry of the rare earth elements is dominated by the +3 oxidation state, with most exhibiting little or no significant redox chemistry. Cerium is a notable exception, its chemistry owing much to the well-established Ce­(III)/Ce­(IV) redox couple.^24–26^ Moreover, the “lanthanide contraction”, known since the early 20th century, results in dramatic variation in ionic radius across a series of otherwise chemically similar elements.^27^ These properties afford the opportunity to study in near-isolation the role of metal size and, by association, steric congestion on the catalytic behavior of congeneric species differentiated only by the identity of the metal center. Indeed, there exist many reports describing a correlation between the ionic radius and catalytic activity of mutually isostructural rare earth metal-based systems for the ROP of lactides and, conversely, an inverse relationship between ionic radius and selectivity (including stereoselectivity) and control.^28–34^

Such a size continuum is not conveniently available for the study of tetravalent metals, as the lanthanides provide for trivalent species. Nonetheless, in the absence of chemical or electrochemical reductants, Ce­(IV) affords a convenient comparator for compounds of the much smaller, redox-inactive Group 4 elements Zr and Hf in their typical +4 oxidation states.

In this context, we herein describe the facile synthesis and catalytic study of two such series of isostructural homoleptic (or *pseudo*-homoleptic), zwitterionic compounds based around various trivalent (Y­(III), La­(III), Pr­(III), Yb­(III)) and tetravalent (Zr­(IV), Hf­(IV), Ce­(IV)) metal centers, encompassing a range of ionic radii. Accordingly, the resulting systems and the redox chemistry of the Ce­(IV) species were to be exploited for investigation of the relationships between metal size and chiral inversion rate, activity, control, and stereoselectivity in the ROP of *rac*-LA.

## Methods

With the exception of the lactide monomer, all reagents and solvents were purchased from commercial suppliers. Manipulations were typically carried out under an inert argon atmosphere using standard laboratory techniques. Polymerization reaction mixtures (50 wt % LA in anhydrous PhCl) were prepared in a glovebox and sealed in 4 mL glass reaction vials, with PTFE-lined melamine resin screw caps before removal and heating in a preheated, thermostatically regulated aluminum heating block. Kinetic data were obtained by carrying out reactions of identical compositions over multiple distinct reaction durations. Samples were homogenized prior to ^1^H NMR spectroscopic analysis by the addition of chloroform-*d*. Complete synthetic methods and characterization data for compounds **2**–**10** and all polymerization reactions can be found in the Supporting Information.

## Results and Discussion

### Catalyst Synthesis and Structure

In addition to **1**,^12,14^ our group has also reported previously the structurally related zwitterionic Sn­(II) species [Sn­(HL^Me^)].^14^ In contrast, the reaction of [Ti­(O*^i^* Pr)_4_] with pro-ligand H_3_L^Me^ has been shown to afford the heteroleptic species [L^Me^Ti­(O*^i^* Pr)],^35^ presumably due to the small size of the Ti­(IV) center disfavoring the required close approach of two coordinated ammonium tris­(phenolate) ligands. In the current work, the reaction of the same pro-ligand with [Hf­(O*^i^* Pr)_4_·HO*^i^* Pr] and [Ce­(O­(CH_2_)_2_OCH_3_)_4_], respectively, readily afforded in high yield [Hf­(HL^Me^)_2_], **2**, and [Ce­(HL^Me^)_2_], **3**. Compounds **2** and **3** are both isostructural with **1** (Scheme 2, Figure 1), with the two ammonium tris­(phenolate) ligands in both cases adopting opposing conformational chirality (a heteroditopic arrangement) in the solid phase. As expected, the Ce­(IV) compound exhibited significantly longer M–O bond distances while the values for **1** and **2** are very similar (mean M–O distances: 2.06, 2.05, and 2.21 Å, for **1**, **2**, and **3**, respectively; see Table 1). Unlike the colorless Group 4 compounds, **3** is an intense brown color. The generality of the structural motif adopted on reaction of pro-ligand H_3_L^Me^ with large tetravalent metal precursors is consistent with the known robustness of **1**; both **2** and **3** are similarly robust toward ambient atmospheric conditions, both in the solid phase and in solution.^14^

**Scheme 2:**
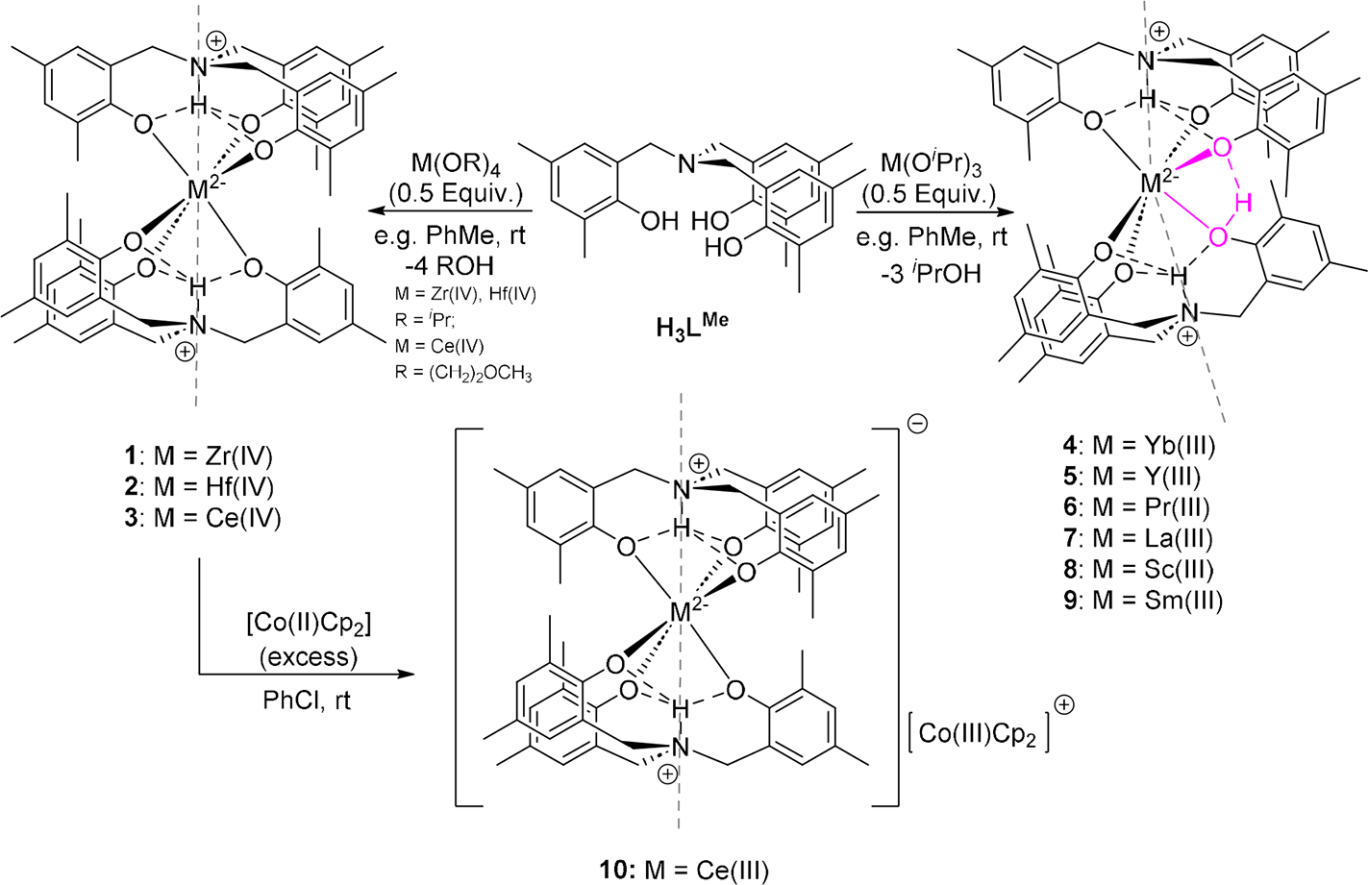
Syntheses and Structures of Compounds **1**–**10** ^a^

a The hydrogen-bonded O–H···O motif of compounds **4**–**9** is highlighted in pink. The N–M–N angle, the value of which corresponds to a compound’s linearity and symmetry, is illustrated with gray, dashed lines. Representations of compounds **1**–**10** do not describe accurate coordination geometries

**Figure 1:**
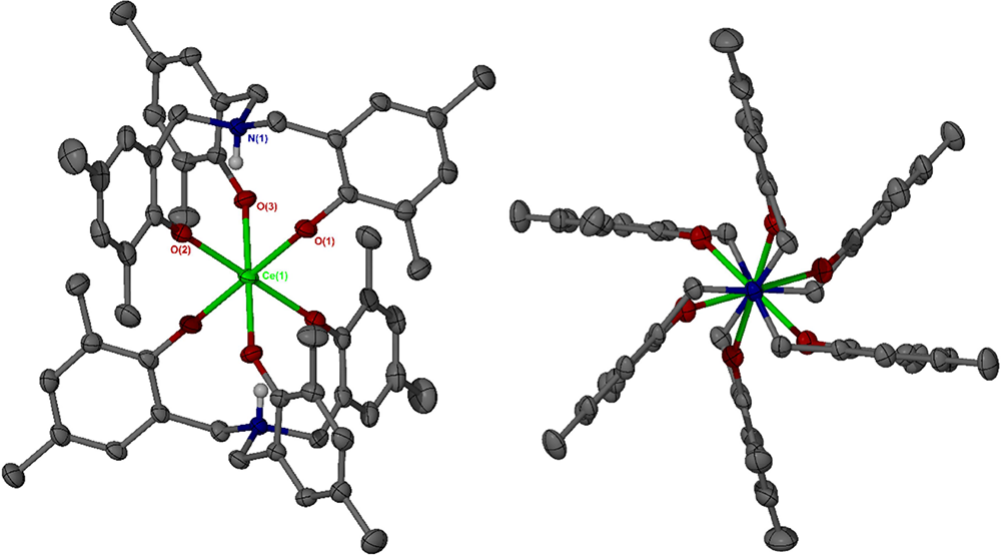
Solid-phase structure of Ce­(IV) compound **3**, including N1–Ce1–N1 axial view, showing the heteroditopic ligand conformation and staggered M–O bonds. Selected structural parameters are provided in Table 1. Ellipsoids are drawn at the 50% probability level. All solvent molecules, carbon-bonded hydrogen atoms and intramolecular N–H···O interactions have been omitted for clarity.

**Table 1:** Selected Structural Parameters for Compounds **1**–**10** in the Solid Phase

			bond distances, Å							angles, deg		
compound (metal)	solvent^a^	literature ionic radius,^b^ Å	M–O1	M–O2	M–O3	M–O4	M–O5	M–O6	mean M–O	O1–M–O2	N1–M–N2	conformation
**1** (Zr)^c^	PhMe	0.72	2.064(2)	2.058(2)	2.057(2)	N/A	N/A	N/A	2.06	N/A	180^d^	heteroditopic, staggered
**2** (Hf)	PhMe	0.71	2.0647(15)	2.0472(16)	2.0470(16)	N/A	N/A	N/A	2.05	N/A	180^d^	heteroditopic, staggered
**3** (Ce(IV))	PhMe	0.87	2.214(2)	2.206(2)	2.203(2)	N/A	N/A	N/A	2.21	N/A	180^d^	heteroditopic, staggered
**4** (Yb)	PhCl	0.87	2.533(4)	2.246(3)	2.150(4)	2.148(4)	2.132(3)	2.126(4)	2.22	61.42(13)	163.17	homoditopic, eclipsed
**4** (Yb)	CDCl_3_	0.87	2.624(2)	2.218(2)	2.159(2)	2.144(2)	2.132(2)	2.103(2)	2.23	63.48(9)	166.3	heteroditopic, staggered
**5** (Y)	PhCl, PhMe	0.9	2.457(6)	2.319(6)	2.182(5)	2.181(5)	2.163(5)	2.160(6)	2.24	61.4(2)	163.31	homoditopic, eclipsed
**5** (Y)	CDCl_3_	0.9	2.6212(14)	2.2512(13)	2.1988(13)	2.1812(13)	2.1618(13)	2.1230(14)	2.26	63.19(5)	166.19	heteroditopic, staggered
**6** (Pr)	PhMe	0.99	2.264(7),^f^ 2.265(7)	2.270(6),^f^ 2.270(7)	2.507(8),^f^ 2.507(8)	N/A	N/A	N/A	2.35	58.7(4)^e^	165.96^d^,^f^	homoditopic, eclipsed
**6** (Pr)	CDCl_3_	0.99	2.636(5)	2.425(5)	2.291(5)	2.277(4)	2.257(5)	2.237(4)	2.35	57.61(16)	156.65	homoditopic, eclipsed
**7** (La)	PhMe	1.03	2.697(5)	2.447(5)	2.333(4)	2.312(4)	2.306(4)	2.302(4)	2.40	57.90(14)	164.35	homoditopic, eclipsed
**7** (La)	CDCl_3_	1.03	2.661(3)	2.465(2)	2.338(2)	2.324(3)	2.302(3)	2.276(2)	2.39	57.43(8)	156.67	homoditopic, eclipsed
**8** (Sc)^g^	PhMe	0.745	2.483(3)	2.103(3)	2.063(3)	2.043(3)	1.998(3)	1.961(3)	2.11	68.23(12)	169.94	heteroditopic, staggered
		0.745	2.542(3)	2.142(3)	2.050(3)	2.028(3)	1.983(3)	1.962(3)	2.12	64.85(11)	167.62	heteroditopic, staggered
**9** (Sm)	PhMe	0.958	2.562(4)	2.408(4)	2.237(3)	2.230(3)	2.220(3)	2.217(3)	2.31	59.28(12)	165.34	homoditopic, eclipsed
**10** (Ce(III))	PhCl	1.01	2.322(3)	2.346(3)	2.337(3)	2.362(3)	2.345(3)	2.324(3)	2.34	N/A	178.79	heteroditopic, staggered

a Identity of any solvent molecules present in the unit cell.

b Literature values.^38^

c Literature values.^14^

d N1–M–N1 angle, due to only half of one molecule of the relevant compound appearing in the asymmetric unit.

e O1–M–O1 angle.

f Half of the molecule in the asymmetric unit, with the ligand disordered over two positions.

g Two molecules of **8** present in the unit cell.

Similar to the capacity of the heavier Group 4 metals to bear two dianionic ammonium tris­(phenolate) ligands, herein we report the reaction and structural characterization of metal compounds formed on reaction of H_3_L^Me^ with the tris­(isopropoxide) compounds of various trivalent metals; Yb­(III), Y­(III), Pr­(III), La­(III), Sc­(III), and Sm­(III). In total, ten single-crystal X-ray structures of these compounds have been obtained (see Figure 2 for selected examples), all of which exhibit zwitterionic ligands and adopt a *pseudo*-homoleptic structure closely related to **1**, **2**, and **3**. Interestingly, this is in contrast to observations made by some of us, and others, that large pentavalent metal centers, Nb­(V) and Ta­(V), consistently favor formation of heteroleptic products under comparable synthetic conditions.^1,13,36,37^ The structure adopted by the M­(III) systems can be generalized as [M­(HL^Me^)­(H_2_L^Me^)], in which neutrality is maintained by retention of an additional proton, formally resulting in one dianionic and one monoanionic ligand. All of these colorless species slowly undergo visible discolouration in the solid phase or in solution over the course of hours or days in ambient air, but are indefinitely stable under inert conditions.

**Figure 2:**
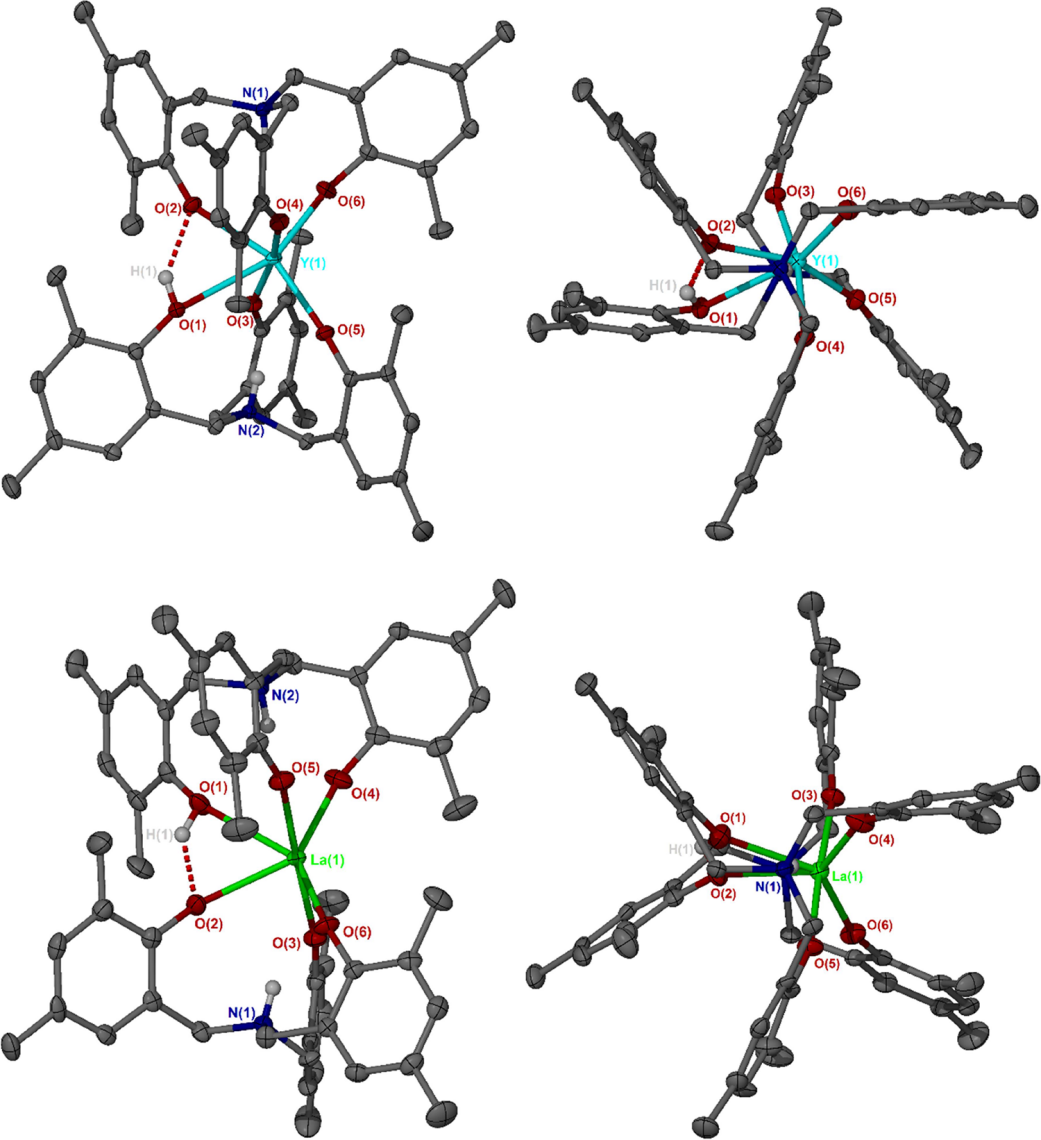
Solid-phase structures of Y­(III) and La­(III) compounds **5** (top) and **7** (bottom), respectively, both crystallized from chloroform-*d.* In both cases the O1–H1···O2 hydrogen bonding motif and displacement of the metal center out of the N1–N2 axis are visible. N1–N2 axial views are included, showing, respectively, the heteroditopic and homoditopic ligand conformations of **5** and **7**, with correspondingly staggered and eclipsed M–O bonds. Selected structural parameters are provided in Table 1. Ellipsoids are drawn at the 50% probability level. All solvent molecules, carbon-bonded hydrogen atoms and intramolecular N–H···O interactions have been omitted for clarity.

Variations in mean M–O bond lengths across both the M­(IV) and M­(III) series of metal compounds, respectively, are in agreement with known values for their ionic radii,^38^ as summarized in Table 1. Moreover, the tetravalent metal compounds generally exhibit shorter M–O distances, consistent with an increased effective nuclear charge, relative to comparable trivalent species. This may correspond to a significant difference in the lability of the metal-coordinating oxygenic moieties of the ligand systems between the M­(III) and M­(IV) species, which is of interest in relation to potential catalytic application.

In the cases of [Yb­(HL^Me^)­(H_2_L^Me^)], **4**, and [Y­(HL^Me^)­(H_2_L^Me^)], **5**, the additional proton was readily located at one of the ligand oxygen atoms in the solid phase. In both cases, there was an associated hydrogen-bonding interaction between the protonated phenolate group and one of the phenolate oxygen atoms of the opposing ligand, resulting in a highly distorted structure, when compared to the symmetric Zr­(IV), Hf­(IV) or Ce­(IV) compounds, **1**, **2**, or **3**, although the specific geometry did exhibit some variation, dependent upon the solvent system from which the sample was crystallized.

In the structures of both **4** and **5**, irrespective of the nature of the solvent from which the sample was crystallized (present in the unit cell), the protonated phenolate group exhibited a significantly elongated metal–oxygen distance relative to the other M–O distances in the same compound, while the phenolate oxygen atom to which this moiety is hydrogen-bonded is also more distant from the metal center, albeit to a lesser extent. Unlike the *pseudo*-octahedral M­(IV) species, **1**–**3**, in which the N1–M–N2 angle is 180°, **4** and **5** exhibit deformation to between 166° and 163°, consistent with the observed presence of the hydrogen bonding interaction between metal-coordinated phenolate moieties of opposing ligands, and their resulting mutual spatial proximity and acute O1–M–O2 angle (generally 61°–63° versus equivalent O–M–O angles in **2** exceeding 90°). Unexpectedly, whereas the solid-phase structures of the compounds of these two intermediate-sized trivalent metals, Y­(III) and Yb­(III), containing aromatic solvent molecules in the unit cell adopted a homoditopic conformation, in which the two ligands present are of similar conformational chirality (denoted as ***P*** *,* ***P*** or ***M*** *,* ***M***, see Scheme 3), the structure in the unit cell of which CDCl_3_ was present, in both cases, described a heteroditopic arrangement. Moreover, whereas in all of the homoditopic systems prepared in the current work (further examples discussed below), the metal–oxygen bonds of the two ligands appear almost completely eclipsed with respect to their positions about the N1–N2 axis (the metal center not falling into alignment with the N atoms to afford a linear N1–M–N2 axis in the M­(III) systems), the heteroditopic forms of **4** and **5** both adopt a staggered arrangement, consistent with **1**–**3**, and the N1–M–N2 angle is also slightly closer to linearity (by approximately 3° in both cases) than in their corresponding homoditopic conformations.

**Scheme 3:**
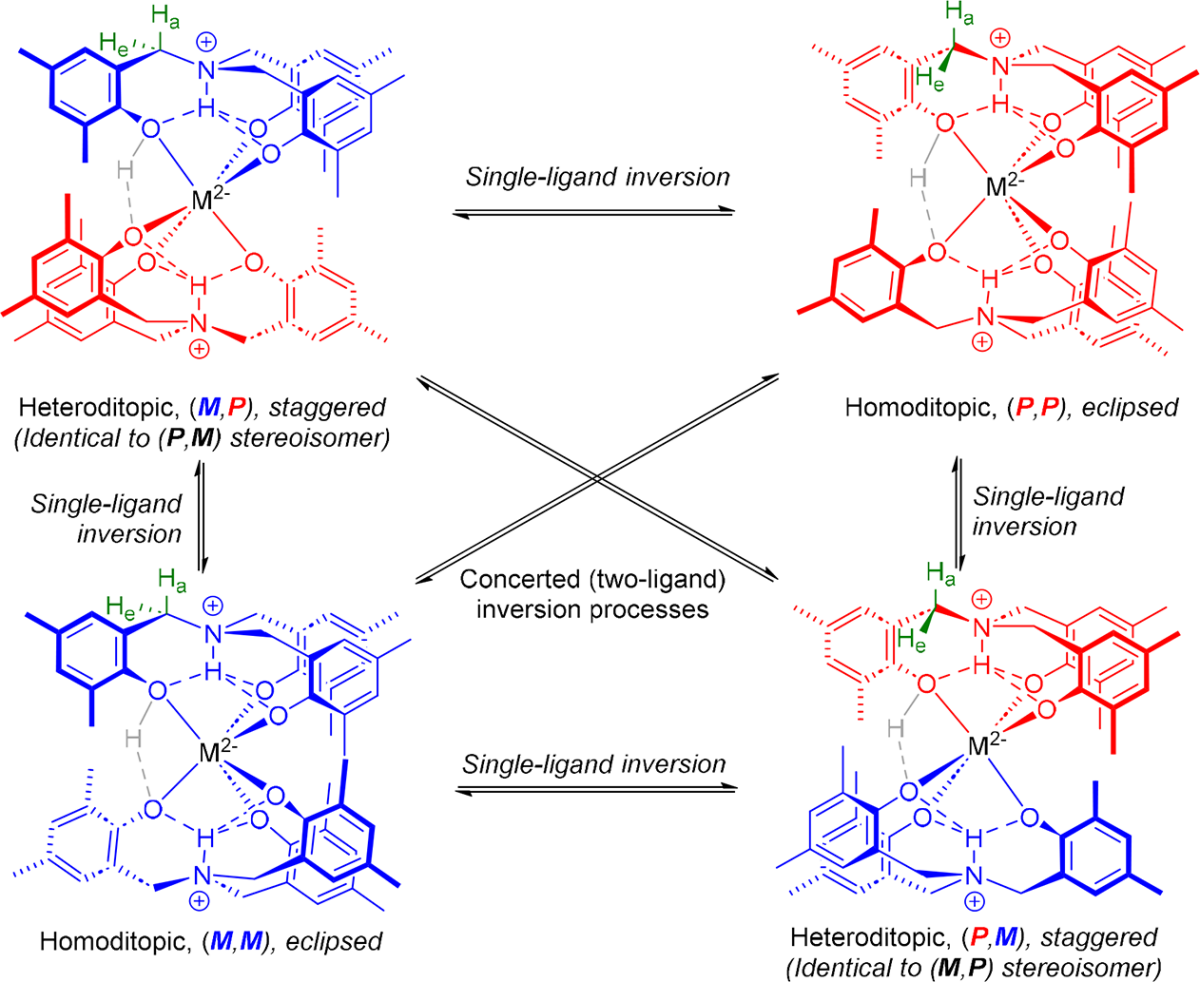
All Plausible Ligand Inversion Processes for Compounds **1**–**10** (in the Absence of Monomer and where the OH Proton is Delocalized on the NMR Time Scale under Solution-Phase Conditions)^a^

a Representative diastereotopic methylene protons are shown in green. An OH···O (protonated phenolate) proton and associated hydrogen bonding motif, placed in a representative static position, is shown in grey, corresponding to its presence only in the cases of neutral M­(III)-containing compounds **4**–**9**.

It is evident from the capacity of both **4** and **5** to adopt distinct conformations in the solid phase, differentiated by inversion of the conformational chirality of the H_3_L^Me^-derived ligand scaffolds, when crystallized from different solvents at ambient temperature, that the energetic difference between the two arrangements is small, and the barrier to the associated inversion process low (Scheme 3). Although the hydrogen atom of the protonated phenolate group was only directly observed in the solid-phase structures of **4** and **5**, wherein CDCl_3_ was present in the unit cell, the structural distortions attributed to the presence and hydrogen-bond donating character of that moiety were consistently apparent in all structures of M­(III) compounds.

In the case of the Pr­(III) compound, [Pr­(HL^Me^)­(H_2_L^Me^)], **6**, in which the metal center is larger than either Yb­(III) or Y­(III), the OH proton could not be directly observed in the solid phase, irrespective of whether CDCl_3_ or toluene (PhMe) was present in the unit cell. However, in the PhMe-containing structure, only half of one molecule of the compound appeared in the asymmetric unit, with the ligand system appearing disordered over two positions. In the La­(III) system, [La­(HL^Me^)­(H_2_L^Me^)], **7**, containing the largest-radius M­(III) center of those described herein, the OH proton was readily refined in the solid-phase structure containing CDCl_3_, but not in that wherein PhMe was present. In both **6** and **7**, the ligands were always arranged in a homoditopic fashion, regardless of the solvent of crystallization. Nonetheless, both species consistently exhibited structural distortions consistent with the presence of a single protonated phenolate moiety and associated hydrogen bonding motif. Accordingly, **6** and **7** are structurally and conformationally highly reminiscent of the homoditopic forms of **4** and **5**, although the N1–M–N2 (N1–M–N1 for **6**) and O1–M–O2 angles were slightly more acute in the larger congeners, consistent with the greater spatial requirements of those metal centers necessitating a more distorted geometry, relative to **1**, **2**, and **3**. Additionally, while remaining conformationally homoditopic, the N1–M–N1/N2 angles of **6** and **7** both became significantly more acute (by approximately 10° and 8°, respectively) when CDCl_3_ was present, relative to PhMe, deviating from linearity to a much greater extent than the heteroditopic conformations adopted by **4** and **5** in the presence of CDCl_3_. High-resolution mass spectrometric analysis of **4**, **5**, **6**, and **7** was, in each case, consistent with the presence of the species observed in the solid phase.

The solid-phase structures of Sc­(III) and Sm­(III) systems, [Sc­(HL^Me^)­(H_2_L^Me^)], **8**, and [Sm­(HL^Me^)­(H_2_L^Me^)], **9**, (containing PhMe in the unit cell), confirmed them to be structurally analogous to the other M­(III) species reported herein. Compound **8**, which, while Sc­(III) is significantly larger than Ti­(IV), represents the smallest-radius metal for which this type of structure has been observed to form,^35^ adopts a heteroditopic ligand conformation in the solid phase, with M–O bonds staggered about the N1–N2 axis. This is notable given the association observed in the cases of **4**–**7** for structures wherein aromatic solvent molecules were present to favor homoditopicity. By contrast, **9**, containing a Sm­(III) center, intermediate in ionic radius between **5** (Y­(III)) and **6** (Pr­(III)), was homoditopic, with this discrepancy being consistent with the observations discussed above. Similarly, the N1–M–N2 angle of **8** was the most linear of all M­(III) systems discussed (see Table 1).

The consistent preference of the protonated phenolate group to form an interligand, rather than intraligand, hydrogen bond is indicative of the structural integrity of the tripodal (H_3_L^Me^)^2–^, or (H_3_L^Me^)^−^, scaffold. In order to accommodate a short hydrogen bond, structural distortion involving rotation and tilting of the two ligands relative to each other is preferred over reorientation of individual aryl groups within a single ligand (Figure 3).

**Figure 3:**
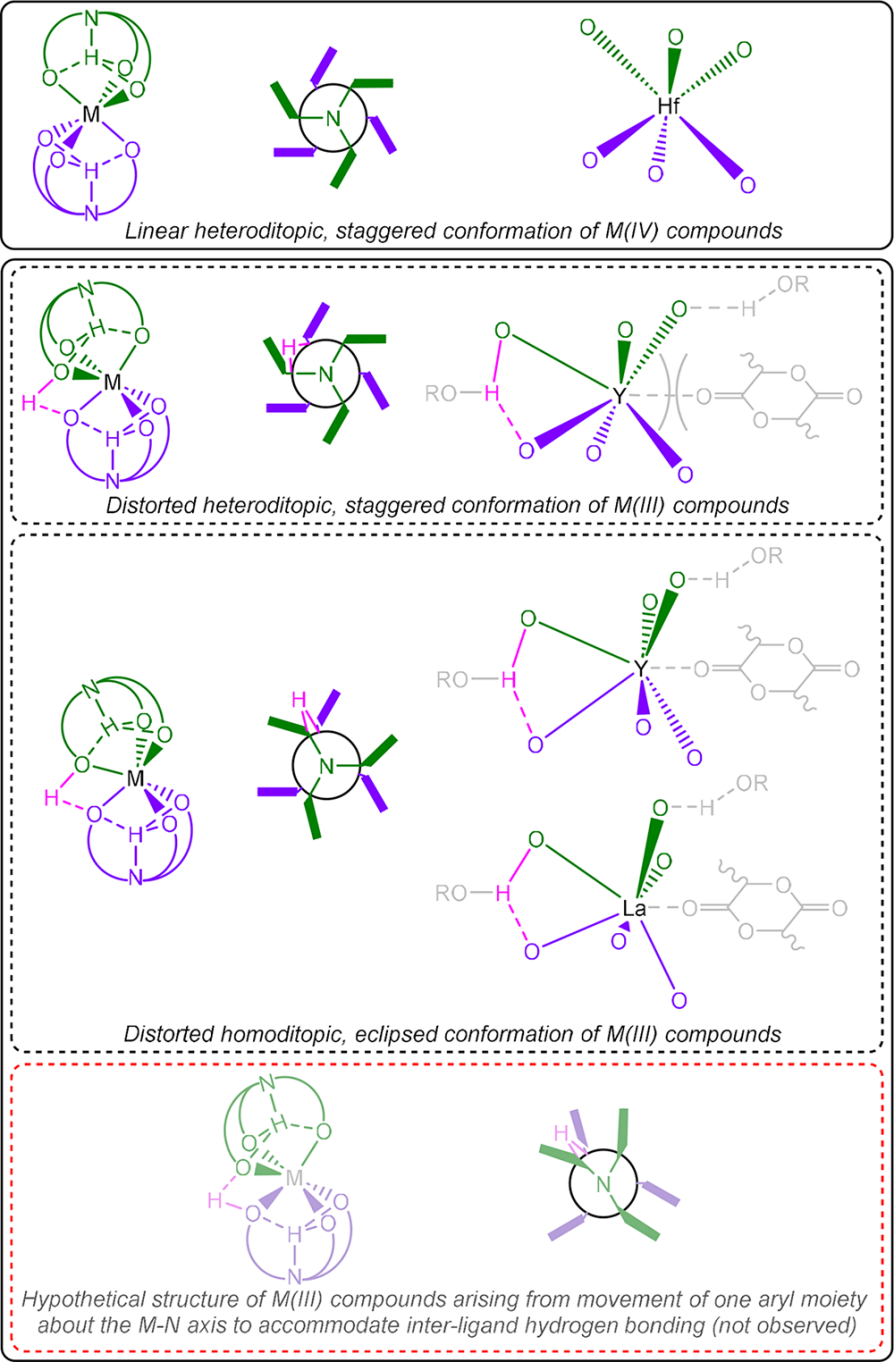
Conformational variation of ammonium tris­(phenolate) compounds. Schematic representations and Newman-type projections, respectively, illustrate the tilting motion required by each ligand in relation to the other and the rotation about the relevant M–N axis, with associated inversion of conformational chirality, observed in the cases of larger metal centers. Representations of the MO_6_ core of selected compounds, drawn from crystallographic data, show the degree to which the metal center is exposed in each case, while the OH proton (pink) has been placed in a representative position, and the putative positions of monomer and co-initiator prior to phenolate dissociation and nucleophilic attack at an activated metal center of similar geometry are represented in gray. A protic co-initiator may occupy a position analogous to that of the OH proton.

It is apparent that the eclipsed arrangement adopted by the M–O bonds of the homoditopic forms of compounds **4**–**7** finds its basis in such mutual reorientation of the two ligands to accommodate the proximity of O1 and O2 demanded by the associated hydrogen bonding interaction. The rotational component of such ligand reorientation, consistently associated with (and thus presumably necessitating) homoditopicity, is increasingly necessary in order to achieve the requisite proximity in the cases of compounds based on larger metal centers. Moreover, the tendency of those larger species to favor such a homoditopic, eclipsed conformation results in an empirically highly exposed face of the metal center located *trans* to the hydrogen bonding motif, arising from dramatic distortion of the *pseudo*-octahedral MO_6_ coordination environment. This, in combination with the associated trend for such systems to exhibit more acute O1–M–O2 angles (corresponding to greater displacement of the metal center from the N1–N2 axis), gives rise to a highly accessible channel between phenolate moieties, which may plausibly permit an approaching monomer greater facility of access to the metal center. Despite the ligand systems’ resistance toward movement of individual aryl groups, it is evident that sufficient flexibility remains, with respect to rotation of those groups about axes parallel to the M–N axis, via repositioning of the methylene moieties, that they are able to adopt the positions necessary to confer, and invert, conformational chirality. Stereocontrol, and selectivity more generally, in the metal-catalyzed ROP of lactones have been widely attributed in the literature to a sterically constrained monomer coordination site at the metal center, characteristically achieved through the use of a conformationally inflexible ancillary ligand.^3^

The apparent inverse correlation between the ionic radius of the metal center present and the system’s amenability to adopting a heteroditopic conformation suggests that, in high-symmetry, linear compounds, this spatial arrangement serves to minimize steric interactions between the two H_3_L^Me^-derived ligands but is sensitive to subtle variation in the coordination geometry about the metal center. Such variation is enforced by the presence of the hydrogen bonding motif involving the protonated phenolate group, which demands greater geometric distortion with increasing metal center size, concomitantly exposing the metal center. This trend is of interest in the current work, given the significant steric demands associated with a lactone monomer approaching a highly coordinated metal center in the course of ROP catalysis and the putative role of the conformational chirality of the ligand in effecting stereocontrol and influencing catalytic activity, *vide infra*. Moreover, the unambiguous relationship between the size of the metal center in an M­(III) compound, and the conformation preferentially adopted in the solid phase further confirms that, even at ambient temperature, the energy surface upon which the various conformational stereoisomers lie is rather flat, and that its topography is readily influenced by the identity of the metal center. More sterically congested species, preferentially heteroditopic in form, may be anticipated to more selectively undergo inversion of conformational chirality as a concerted process involving both ligands, whereas larger systems are likely comparatively more amenable to independent inversion of individual ligands.

^1^H NMR spectroscopic analyses, including variable-temperature studies, suggest that in solution, all species for which data were obtained adopt a heteroditopic arrangement (see below). This further illustrates the capacity of those compounds isolated as homoditopic stereoisomers in the solid phase to readily undergo such nonconcerted inversion processes. However, we note that, while in the absence of a coordinating substrate concerted inversion from an initial heteroditopic conformation results in no overall change to the chirality of the species, this is not necessarily the case in the presence of the monomer, which may confer overall asymmetry, as well as influencing the facility of inversion more generally. The ability of **4**–**7** to also undergo nonconcerted inversion processes presumably further increases geometric and stereochemical variation and instability about any monomer coordination site, with likely mechanistic implications. It is plausible, too, that in the context of a catalytic regime, the relationship between the size of the metal center and the flexibility of the ligand conformation may also apply to the M­(IV) series with similar mechanistic, stereochemical relevance.

In solution-state ^1^H NMR spectroscopic analyses of **4**, **5**, **7**, and **8** (the paramagnetic nature of Pr­(III) and Sm­(III) largely precluding such analysis for **6** and **9**), each compound appears to be *C*_3_ *-*symmetric, with equivalency between all comparable phenolate ^1^H environments within each system. For **4**, **5**, and **7** this was confirmed to be the case across a wide temperature range (223.15–≥323.15 K, see below), indicating that, in solution, the OH proton is likely delocalized on the NMR time scale between all phenolate oxygen atoms, resulting in no persistent distortion, and thus apparent *C*_3_ symmetry about the N1–N2 axis. Accordingly, the OH proton is not directly observable under normal conditions via ^1^H NMR spectroscopy. Nonetheless, it is plausible that distortion similar to that observed in the solid phase may occur transiently in association with such a dynamic process as proton delocalization. It is therefore unclear whether such observed *pseudo*-*C*_3_ symmetry may, in a catalytic context, provide an analogy to the truly *C*_3_-symmetric M­(IV) systems or instead offer a distinct conformational environment.

In addition to crystallographic analyses revealing subtle trends in relation to ligand conformation and symmetry, variable temperature ^1^H NMR (VT-NMR) spectroscopic analyses of compounds **1**–**7** (**1**–**3** in solution in *protio*-chlorobenzene and **4**–**7** in chloroform-*d*) sought to establish the relationship between the size of the metal center and the rate of solution-phase inversion of conformational chirality prior to catalytic studies. Specifically, where possible, coalescence was observed in resonances corresponding to inversion of diastereotopic proton environments at the methylene groups adjacent to the bridgehead nitrogen atom of the ligand scaffolds (Scheme 3). The paramagnetic character of **6** precluded the definitive identification of the relevant signals. Nonetheless, there appeared to be little correlation between the size of the metal center and the rate of inversion for otherwise analogous compounds. However, the M­(III) systems were much more conformationally dynamic than the sterically congested M­(IV) species, with the coalescence temperatures, *T*_c_, of **4** and **5** being in the region of 273 K, and that of **7** being approximately 263 K, whereas those of the M­(IV) series, **1**–**3**, were consistently around 353 K, consistent with the highly interdigitated nature of those small, linear heteroditopic species’ ligands. Accordingly, for each system, Δ*G* ^‡^ was calculated for the inversion process (Table 2).^39^ However, it could not be ascertained from the VT-NMR data whether the observed inversion processes involved concerted movement of interdigitated ligands or independent motion of individual ligands, precluding further delineation of the relationship between metal size and the manner in which conformational inversion occurs. Similarly, it has not been determined whether the more dynamic nature of the M­(III) systems is attributable exclusively to the radii of the metal centers, or if the anticipated increase in ligand lability arising from the presence of the OH proton, and lower effective nuclear charge, *Z*_eff_, may also contribute.

**Table 2:** Coalescence Temperatures, *T*_c_, and Calculated Δ*G* ^≠^ Values for **1**–**7**, Determined via Variable Temperature ^1^H NMR Spectroscopy^39^

compound	metal center	*T*_c_, K	Δ*G* ^‡^, kJ mol^–1^
**1**	Zr(IV)	353	64.74
**2**	Hf(IV)	353	64.80
**3**	Ce(IV)	353	64.53
**4**	Yb(III)	273	49.85
**5**	Y(III)	273	49.98
**6**	Pr(III)	N/A	N/A
**7**	La(III)	263	48.00

On the basis of the structural trends discussed above, and the structural variation between M­(IV) and M­(III) systems, the catalytic activity and selectivity of each of the trivalent metal-based species in the polymerization of *rac*-LA were quantified both in the presence and absence of an exogenous alcohol co-initiator. The tetravalent systems, of which **1** has previously been subject to detailed mechanistic investigation,^2^ were applied to the ROP of *rac*-LA in the presence of an alcohol.

### Polymerization Studies

Representing a range of ionic radii in both the M­(III) and M­(IV) series, complexes **1**–**7** were selected for investigation of catalytic activity in the ROP of lactide. Kinetic studies of the polymerization of *rac*-LA in the presence of each respective catalyst candidate were undertaken using multiple identical reaction mixtures of 50% wt/vol monomer in anhydrous chlorobenzene ([LA] = 3.47 mol dm^–3^). Such “near-melt” conditions ensure effective mixing on a small scale, while also requiring lower reaction temperatures than those required for solvent-free ROP processes, permitting acquisition of data with sufficient resolution for kinetic analysis, while maintaining its broad applicability to industrial contexts. Reaction vials were placed into a heated aluminum block and then sequentially removed and analyzed (to determine conversion, polymer molecular weight, and stereochemical enrichment) at various time points, to facilitate observation of reaction progress. The monomer was purified by recrystallization prior to use.

When the polymerization of *rac*-LA was undertaken at 120 °C (Al block temperature), the tetravalent systems **1**, **2** and **3** all exhibited very similar reaction rates (*k*_obs_ = 7.6 × 10^–3^ min^–1^, *k*_obs_ = 6.5 × 10^–3^ min^–1^, and *k*_obs_ = 6.0 × 10^–3^ min^–1^, respectively, where monomer, catalyst and alcohol co-initiator 4-methylbenzyl alcohol, 4-MeBnOH, were in the molar ratio [*rac*-LA]:[Cat.]:[4-MeBnOH] = 1000:1:3; see Table 4, Figure 4), with each system requiring at least 8 h to attain equilibrium conversion (95%). **3** had initially been anticipated to outperform **1** and **2**, due to the larger ionic radius of Ce­(IV) relative to the Group 4 congeners.^28–34^ However, the minimal difference in the respective activity observed for the three systems is consistent with their similar *T*_c_ values, determined via VT-NMR experiments. This indicates that each propagation event may be mechanistically associated with concerted rearrangement of the ligand scaffold(s), as opposed to heteroselectivity in the cases of **1** and **2** (see below), simply originating from inversion occurring spontaneously at a rate commensurate with that of propagation. Compound **3**, however, offered markedly reduced stereoselectivity in comparison to **1** and **2**, which were found to significantly favor heterotactic propagation under the current conditions (*P*_r_ = 0.79 and *P*_r_ = 0.80 versus *P*_r_ = 0.55, for **1**, **2** and **3**, respectively; see Table 3, Figure 5), this being unexpected in the context of concomitant rates, but nonetheless characteristic of the Ce­(IV) system having a less constrained coordination environment. **3** was also distinct from **1** and **2** in exhibiting a gradual reduction in *P*_r_ with increasing reaction time and conversion (see Table S2, Figure S16 in the Supporting Information). Such a progressive reduction in *P*_r_ likely corresponds to the occurrence of side reactions deleterious to stereosequence retention, such as transesterification or epimerization, although the persistence of some heterotactic enrichment of the polymer product obtained even at long reaction times indicates that the extent of proliferation of either process was low.

**Table 3:** Selected Polymerization Data for Compounds **1**–**7** and **10**

entry	cat.	metal center	[*rac*-LA]/[Cat.]/[4-MeBnOH]^d^	*T*, °C	duration, min	conversion,^e^ %	*P*_r_ ^f^	*M*_*n*_^Theo^,^g^ g mol^–1^	*M*_*n*_^SEC^,^h^ g mol^–1^	*Đ*_M_ ^h^
1^a^,^b^	**1**	Zr(IV)	1000:1:3	120	480	96	0.79	46,242	71,900	1.68
2^a^,^b^	**1**	Zr(IV)	1000:1:10	120	960	96	0.77	13,958	23,500	1.68
3^a^,^b^	**2**	Hf(IV)	1000:1:3	120	480	95	0.80	45,762	61,950	1.74
4^a^,^b^	**2**	Hf(IV)	1000:1:10	120	960	96	0.76	13,958	23,200	1.71
5^a^,^b^	**3**	Ce(IV)	1000:1:3	120	960	95	0.55	45,762	60,450	1.63
6^a^,^b^	**3**	Ce(IV)	1000:1:10	120	240	82	0.57	11,952	18,050	1.59
7^a^,^b^	**3**	Ce(IV)	1000:1:10	120	960	95	0.57	13,827	22,750	1.65
8^a^,^b^	**4**	Yb(III)	1000:1:0	120	240	90	0.57	N/A	59,550	1.72
9^a^,^b^	**4**	Yb(III)	1000:1:3	120	240	96	0.55	46,242	38,400	1.80
10^a^,^b^	**4**	Yb(III)	500:1:0	120	240	96	0.55	N/A	49,150	1.69
11^a^,^b^	**4**	Yb(III)	250:1:0	120	240	96	0.55	N/A	33,100	1.99
12^a^,^b^	**4**	Yb(III)	1000:1:10	120	240	96	0.53	13,958	20,200	1.78
13^a^,^b^	**5**	Y(III)	1000:1:0	120	120	95	0.57	N/A	62,700	1.66
14^a^,^b^	**5**	Y(III)	1000:1:3	120	120	97	0.57	46,723	47,150	1.65
15^a^,^b^	**5**	Y(III)	500:1:0	120	120	97	0.55	N/A	51,350	1.65
16^a^,^b^	**5**	Y(III)	250:1:0	120	120	97	0.53	N/A	37,700	1.63
17^a^,^b^	**5**	Y(III)	1000:1:10	120	60	97	0.53	14,102	22,150	1.60
18^a^,^b^	**6**	Pr(III)	1000:1:0	120	60	95	0.53	N/A	73,200	1.81
19^a^,^b^	**6**	Pr(III)	1000:1:3	120	30	95	0.51	45,762	53,250	1.58
20^a^,^b^	**6**	Pr(III)	500:1:0	120	60	96	0.51	N/A	69,400	1.56
21^a^,^b^	**6**	Pr(III)	250:1:0	120	60	97	0.55	N/A	45,900	1.59
22^a^,^b^	**6**	Pr(III)	1000:1:10	120	60	97	0.51	14,102	21,650	1.63
23^a^,^b^	**7**	La(III)	1000:1:0	120	60	92	0.55	N/A	85,800	1.58
24^a^,^b^	**7**	La(III)	1000:1:3	120	30	94	0.55	45,281	51,450	1.59
25^a^,^b^	**7**	La(III)	500:1:0	120	60	96	0.53	N/A	68,700	1.58
26^a^,^b^	**7**	La(III)	250:1:0	120	60	97	0.49	N/A	44,300	1.60
27^a^,^b^	**7**	La(III)	1000:1:10	120	60	97	0.49	14,102	19,150	1.72
28^a^,^c^	**10**	Ce(III)	1000:1:3	120	2	61	0.62	21,250	31,400	1.48
29^a^,^c^	**7**	La(III)	1000:2:3	80	240	98	0.49	47,203	47,350	1.65
30^a^,^c^	**10**	Ce(III)	1000:2:3	80	60	95	0.62	45,762	30,450	1.61

a Conditions: Vessels placed in an aluminum heating block with thermocouple, preheated to temperature *T*, and with magnetic stirring.

b 500 ± 5 mg *rac*-LA in 500 μL anhydrous PhCl dosed as a stock solution of Cat. + 4-MeBnOH where relevant.

c 400 ± 5 mg *rac*-LA in 400 μL anhydrous PhCl dosed as a stock solution of Cat. + 4-MeBnOH.

d Molar ratio.

e Conversion determined via ^1^H NMR spectroscopy, by integration of LA and PLA methine resonances.

f *P*_r_ calculated via polymer microstructure analysis (^1^H­{^1^H} NMR); *P*_r_ = √(2-[*sis*]).^40^

g *M*_n_^Theo^ assuming an immortal kinetic regime, and a catalyst bearing no functional initiating group, calculated from conversion and alcohol concentration: ${M_{n}}^{\mathrm{Theo}}=\left\{\left(M_{r,\mathrm{LA}}\times \frac{{\%}_{\mathrm{conv}}}{100}\times \frac{\left[\mathrm{LA}\right]}{\left[4-\mathrm{MeBnOH}\right]}\right)+M_{r,4-\mathrm{MeBnOH}}\right\}$ .

h Determined via size-exclusion chromatography (SEC) in THF, using a refractive index detector calibrated against polystyrene standards of known molecular weight.

**Figure 4:**
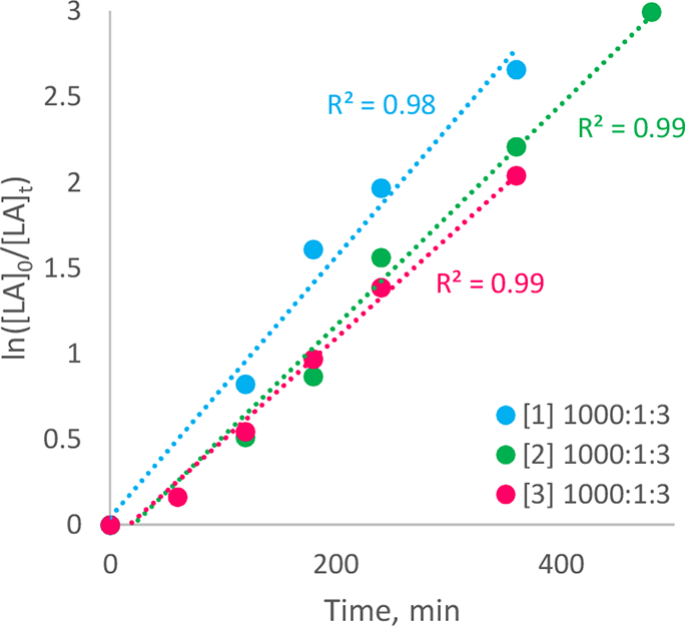
Semilogarithmic initial rate plots for the ROP of *rac*-LA in the presence of 0.1 mol % of catalysts **1**, **2**, and **3** (corresponding to Zr, Hf, and Ce­(IV)) and 0.3 mol % 4-MeBnOH at 120 °C in anhydrous PhCl.

**Figure 5:**
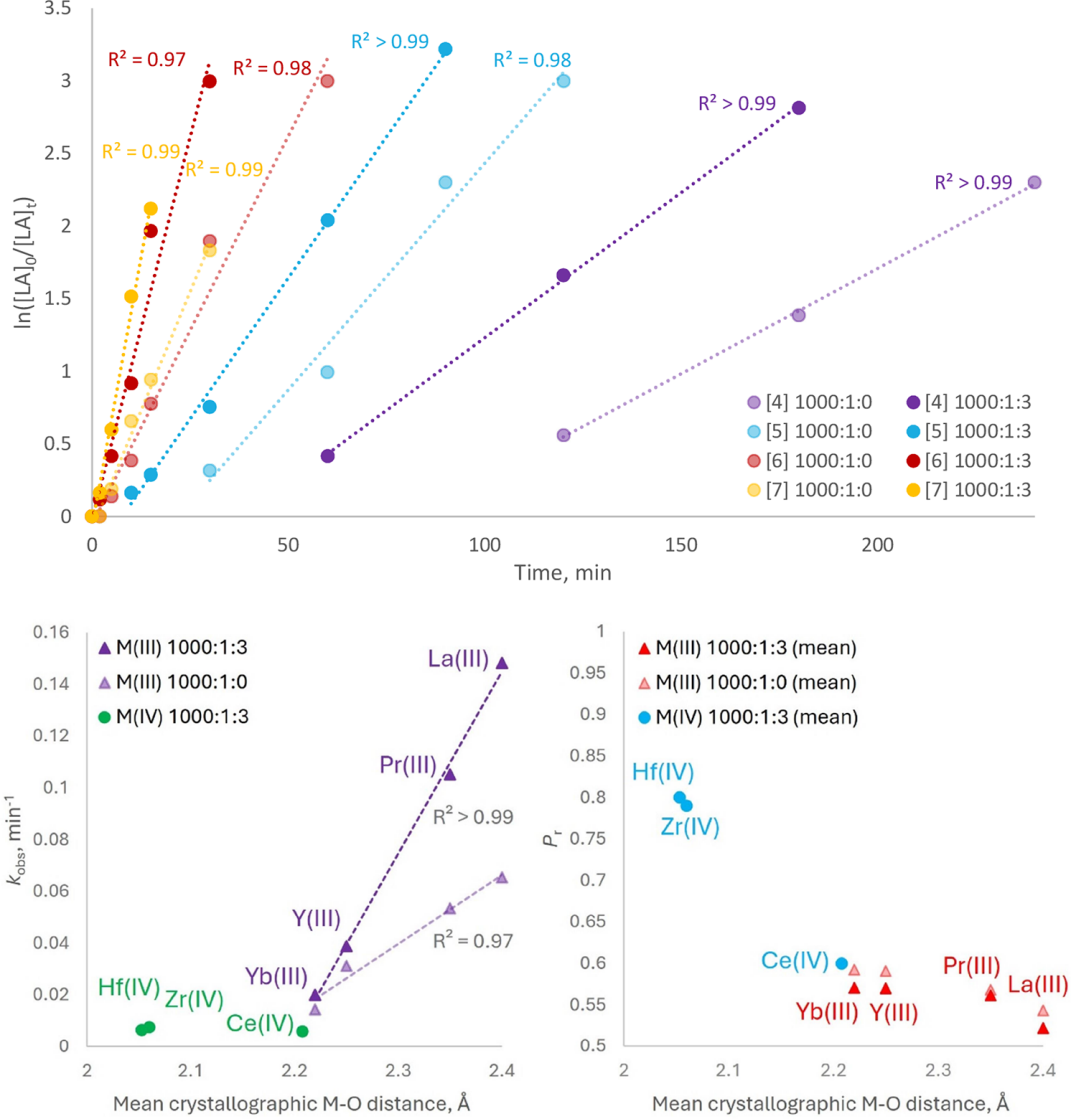
Top: Semilogarithmic initial rate plots for the ROP of *rac*-LA in the presence of 0.1 mol % of catalysts **4**, **5**, **6**, and **7** (corresponding to Yb, Y, Pr, and La) variously in the presence and absence of 0.3 mol % exogenous 4-MeBnOH, at 120 °C in anhydrous PhCl. Bottom: Plots of *k*_obs_ and mean product *P*_r_, respectively, against mean solid-phase catalyst M–O bond distance for the ROP of *rac*-LA in the presence of 0.1 mol %, variously, of catalysts **1**–**3** and 0.3 mol % 4-MeBnOH, and, variously, of 0.1 mol % catalysts **4**–**7**, both in the presence and the absence of 0.3 mol % 4-MeBnOH. Data points are each labeled with the identity of the corresponding metal center for clarity. Where two solid phase structures of a catalyst have been collected, the mean M–O distance has been calculated using data from both structures.

Stereoselectivity in the ROP of lactides is typically attributed to one of two general mechanisms of kinetic control: chain end control and enantiomorphic site control.^3,41–45^ In the former, the stereochemistry of the growing chain end (corresponding to the most recently inserted monomer unit) determines the relative probability of the following propagation event being either *syndio* (giving rise to heterotactically enriched PLA) or *iso* in nature. Catalyst systems adherent to a chain-end control mechanism can exhibit a preference for either heteroselective or isoselective propagation. Enantiomorphic site control typically occurs in the presence of a chiral catalyst species, wherein the morphology of the ligand environment surrounding the monomer coordination site preferentially accommodates one stereoisomer of the monomer, irrespective of the chain end stereochemistry. In a classical enantiomorphic site control mechanism, wherein the morphology of the enantiomorphic site is persistent and the stereochemistry of the growing chain end has no significant influence, heterotactic enrichment is never favored.

We have previously reported that *pseudo*-*C*_3_ *-*symmetric, heteroleptic Zr­(IV), Hf­(IV), and germanium­(IV) alkoxides, supported by a bulky *tert*-butyl-substituted amine tris­(phenolate) ancillary ligand are able to afford highly heterotactic PLA when applied to the ROP of *rac*-LA (0.78 ≤ *P*_r_ ≤ 0.98).^11,12,46^ A similar Ce­(IV) system has very recently been reported to be comparably stereoselective.^47^ Such selectivity has been attributed to the presence of a dynamic enantiomorphic site, in which inversion of the conformational chirality of the ligand scaffold occurs on a time scale commensurate with that of propagation. However, the causal mechanistic association between such conformational rearrangement and the heteroselective propagation event has not been conclusively elucidated.^11,12,47^ Notably, other simple *C*_3_-symmetric species, exhibiting helical chirality, have been reported to afford remarkable isoselectivity in the ROP of *rac*-LA, presumably in the absence of dynamic conformational inversion.^18^ Moreover, there exist many examples in the literature of *C*_3_-symmetric and, in particular, helical systems being applied with high selectivity to various asymmetric catalytic transformations, notably including the diverse reactivity of Shibasaki’s rare earth-based bimetallic binolate systems.^26,48–52^

The observed propensity of **1** and **2** for effecting significantly heterotactic ROP may, in similarity to the Zr­(IV), Hf­(IV), and Ge­(IV) alkoxide systems, be attributed to the presence of a dynamic enantiomorphic site arising from the *C*_3_-symmetric, paddlewheel-like, ligand environment.^11,12,46^ However, the ligand-assisted activated monomer mechanism under which **1** and, presumably, **2** operate in polymerization catalysis is distinct from the classical coordination–insertion pathway favored by metal alkoxide catalysts, including the aforementioned *pseudo*-*C*_3_-symmetric species.^11,12,46^ Moreover, any such mechanism is likely complicated in the case of **1**, **2**, and related systems, by the presence of two tripodal ligands, which may conceivably double the number of distinct stereochemical environments that can be adopted by the active catalyst in the presence of an asymmetric monomer, relative to the heteroleptic alkoxide systems.^11,12,46^

In the cases of all tetravalent catalysts, **1**–**3**, polymer dispersity was relatively high, and in the presence of **1** and **2**, the value of *Đ*_M_ increased with conversion. However, given the retention of a high degree of heterotactic enrichment (*P*_r_ ∼ 0.8), particularly in the case of the smallest-radius Hf­(IV)-based system, **2**, this may correspond primarily to imperfect adherence to an ideal immortal kinetic regime (defined by the occurrence of nonrate-determining chain transfer processes in the presence of an alcohol co-initiator) under the reaction conditions, rather than uncontrolled transesterification activity. Furthermore, the observed degree of heteroselectivity is significantly greater under the current conditions than when **1** has been used previously under high-temperature (174 °C), solvent-free conditions (then, *P*_r_ ∼ 0.67). Notably, however, we have previously confirmed that **1** is able to afford PLA of very narrow dispersity when used under such industrially relevant conditions, perhaps attributable simply to more efficient mixing and lower melt viscosity at high temperature.^2^ Additionally, the molecular weight, relative to polystyrene standards, of PLA produced in the presence, variously, of **1**, **2**, and **3**, increased linearly with conversion in all cases, characteristic of a well-controlled polymerization, with the absolute values exhibiting close agreement between the three systems. SEC analysis of polymer samples produced in the presence, respectively, of **1** and **2** revealed that at low or moderate conversion, a single narrow molecular weight distribution was present, with apparent conversion to a second, broader distribution, with a distinct modal (peak *M*_p_^SEC^) value, but comparable *M*_n_^SEC^. This is consistent with near-complete suppression of transesterification in the presence of a high monomer concentration, with some very limited proliferation occurring at higher conversion. **3**, by contrast, afforded a broad polymer molecular weight distribution irrespective of reaction progress, consistent with more uncontrolled transesterification. Moreover, while kinetic studies for **1**, **2** and **3** were undertaken such that [*rac*-LA]:[Cat.]:[4-MeBnOH] = 1000:1:3, in each case additional reactions, wherein [*rac*-LA]:[Cat.]:[4-MeBnOH] = 1000:1:10, were used to successfully confirm that polymer molecular weight could be manipulated via variation of the alcohol concentration, indicative of a kinetic regime exhibiting immortal character.

Kinetic studies in the presence of **4**, **5**, **6**, and **7** were undertaken using identical conditions and methods to those used for **1**, **2**, and **3**, although given the structural distinction, and associated possibility of mechanistic deviation, from **1**,^2^ data was acquired both in the presence and absence of three equivalents of 4-MeBnOH. In all cases, polymer molecular weight increased with reaction conversion (see Figure 6 and Tables S3–S6 in the Supporting Information), although greater linearity was observed in the presence of 4-MeBnOH than when it was absent. Control was enhanced in the presence of **4** and **5**, relative to **6** and **7**, consistent with greater kinetic control arising from a more spatially constrained monomer coordination environment. In the absence of 4-MeBnOH, polymer molecular weight values were typically higher than in comparable cases wherein the co-initiator was present. Initiation in such cases is likely attributable to the presence of protic impurities in the lactide feed.^53^ When additional reactions were undertaken using each respective M­(III) system, for which the catalyst loading was varied in the absence of 4-MeBnOH ([*rac*-LA]:[Cat.]:[4-MeBnOH] = 500:1:0 and [*rac*-LA]:[Cat.]:[4-MeBnOH] = 250:1:0, respectively), there was a modest inverse relationship between catalyst loading and polymer molecular weight at equilibrium conversion (∼95%). However, the magnitude of this effect was always much smaller than would be expected, for example, in the case of quantitative, stoichiometric initiation by the catalyst, further consistent with initiation instead being facilitated by the presence of protic impurities.^53^

**Figure 6:**
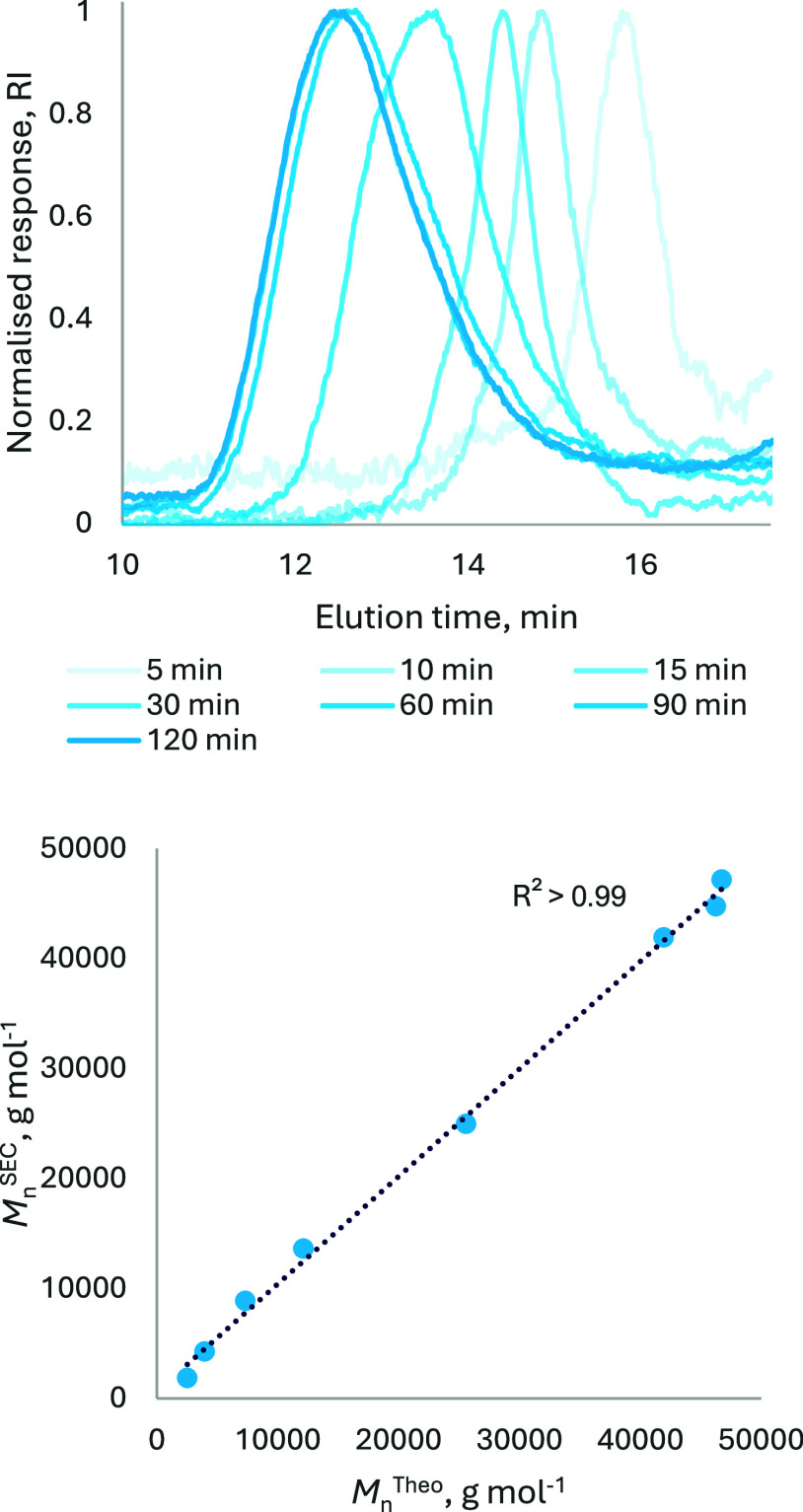
Top: Stacked normalized SEC traces for the products of the ROP of *rac*-LA in the presence of 0.1 mol % **5** and 0.3 mol % 4-MeBnOH after various reaction durations. Bottom: A plot, constructed from the same data, shows a linear correlation between *M*_n_^SEC^ and *M*_n_^Theo^.

In the cases of all M­(III) species, **4**–**7**, the observed rates significantly exceeded those observed for **1**–**3**, with equilibrium conversion being attained after reaction durations of between 0.5 and 4 h, consistent with the larger ionic radii of the M­(III) systems. The presence of 4-MeBnOH always elicited a significant rate enhancement relative to when it was absent, and this effect became more pronounced with increasing ionic radius (Table 4, Figure 5). In the presence of 4-MeBnOH the rates afforded by the M­(III) systems exceeded those of the M­(IV) systems by a factor of between 2.6–3.3 in the case of the least active M­(III) system (see below), **4**, and 20–25 where the most active system, **7**, was used. Nonetheless, values of *Đ*_M_ were generally similar for the polymer products produced in the presence of all of the various M­(III) and M­(IV) species (with the exception of those corresponding to **4** which somewhat higher than those associated with **5**–**7**), suggesting that under industrial conditions (see below), it may be feasible to use protocols based on such systems as **4**, **5**, **6**, or **7** to prepare PLA of similarly high molecular weight and low dispersity as that afforded by the formulation of **1** that we have reported previously,^2^ but with much greater efficiency than is achievable with that system. Indeed, adaptation of such a formulation process to accommodate the current systems is an attractive objective, although the rare-earth-element-based catalysts, **3**–**7**, are also empirically much more soluble in conventional organic solvents than **1** or **2**. In the cases of both **4** and, to a lesser extent, the second-least-active system, **5**, an induction period was readily discernible from the kinetic data. The origin of this effect is unclear, but it may be attributed to slow activation of the catalyst, consistent with the small size of the metal center and presence of short M–O interactions and with the increased dispersity of polymer samples produced in the presence of **4**.

**Table 4:** Kinetic Data, Acquired Using Ex Situ (Parallel Reactions) Methods, for the ROP of *rac*-LA in the Presence, Variously, of Catalysts **1**–**7** and **10**

entry	cat.	metal center	[*rac*-LA]/[Cat.]/[4-MeBnOH]^d^	*T*, °C	*k*_obs_,^e^ min^–1^	*k*_obs_,^e^ h^–1^
1^a^,^b^	**1**	Zr(IV)	1000:1:3	120	7.6 × 10^–3^	0.456
2^a^,^b^	**2**	Hf(IV)	1000:1:3	120	6.5 × 10^–3^	0.390
3^a^,^b^	**3**	Ce(IV)	1000:1:3	120	6.0 × 10^–3^	0.360
4^a^,^b^	**4**	Yb(III)	1000:1:0	120	1.5 × 10^–2^	0.870
5^a^,^b^	**4**	Yb(III)	1000:1:3	120	2.0 × 10^–2^	1.20
6^a^,^b^	**5**	Y(III)	1000:1:0	120	3.1 × 10^–2^	1.87
7^a^,^b^	**5**	Y(III)	1000:1:3	120	3.9 × 10^–2^	2.33
8^a^,^b^	**6**	Pr(III)	1000:1:0	120	5.4 × 10^–2^	3.21
9^a^,^b^	**6**	Pr(III)	1000:1:3	120	0.11	6.31
10^a^,^b^	**7**	La(III)	1000:1:0	120	6.5 × 10^–2^	3.92
11^a^,^b^	**7**	La(III)	1000:1:3	120	0.15	8.90
12^a^,^c^	**10**	Ce(III)	1000:1:3	120	N/A	N/A
13^a^,^c^	**7**	La(III)	1000:2:3	80	1.7 × 10^–2^	1.04
14^a^,^c^	**10**	Ce(III)	1000:2:3	80	8.9 × 10^–2^	5.36

a Conditions: Vessels placed in an aluminum heating block with thermocouple, preheated to temperature *T*, and with magnetic stirring.

b 500 ± 5 mg *rac*-LA in 500 μL anhydrous PhCl dosed as a stock solution of Cat. + 4-MeBnOH where relevant.

c 400 ± 5 mg *rac*-LA in 400 μL anhydrous PhCl dosed as a stock solution of Cat. + 4-MeBnOH.

d Molar ratio.

e Observed rate constant, *k*_obs_, determined via the maximum rate method, wherein *k*_obs_ is equal to the gradient of the linear region of a semilogarithmic plot, ln­([LA]_0_/[LA]_*t*_) versus time, *t*.

Additional polymerization reactions were undertaken using each M­(III) catalyst in the presence of an increased concentration of 4-MeBnOH ([*rac*-LA]:[Cat.]:[4-MeBnOH] = 1000:1:10). A corresponding reduction in molecular weight was observed in each case, without any significant change in *Đ*_M_, confirming the occurrence of nonrate-determining chain transfer activity, characteristic of an immortal polymerization process. The higher concentration of 4-MeBnOH also appeared to enhance the reaction rate, evident in the cases of **4** and **5**, for which reaction duration(s) were used that permitted attainment of subequilibrium conversion, therefore enabling comparison. This is consistent with reports made previously by our group and others of immortal ROP processes deviating from the anticipated zero-order rate dependence with respect to the alcohol co-initiator below a certain threshold value for the concentration of that component.^2,54–56^ The origin of this effect is unclear, but in the current systems, it was initially considered ascribable to either:

1. Greater exposure of the active metal center arising from enhanced lability of the metal-phenolate bonds due to stabilization of the anionic phenolate group by protic alcohol moieties, or other structural changes arising from interaction between the catalyst and alcohol
2. Formation of a transition state in the rate-determining step involving several alcohol molecules, such as a cyclic system, as has been calculated for various processes taking place in protic media.^57,58^

In marked contrast to the tetravalent metal-based catalysts, kinetic studies of the M­(III) series revealed a clear correlation between ionic radius (literature values,^38^ or mean crystallographic M–O distance) and the observed rate constant *k*_obs_ for the ROP of *rac*-LA, both in the presence (*R* ^2^ > 0.99 where the relationship is assumed to be linear) and absence (*R* ^2^ > 0.97) of 4-MeBnOH. Given the minimal variation in *T*_C_ between **4**, **5**, and **7**, as ascertained via VT-NMR experiments, we tentatively suggest that this trend finds its origin in the increasing deviation of the larger M­(III) systems from the highly symmetric conformation observed for the M­(IV) species **1**, **2**, and **3**, characterized by a progressively more acute O1–M–O2 angle in their respective solid phase structures, and thus greater exposure of the opposing face of the metal center (Figure S20 in the Supporting Information). While not readily quantified, the greater preference of the larger systems toward adopting a homoditopic conformation in the solid phase, with correspondingly eclipsed M–O bonds enhancing exposure of the metal center, may also find analogy in their solution-phase behavior, further contributing to the observed trend. Moreover, while **4**, **5**, **7**, and, presumably, **6** exhibit mutually similar *T*_C_ values, these are consistently much lower than those of **1**, **2**, and **3**, making it conceivable that for these systems, spontaneous inversion of conformational chirality can indeed occur independently of the propagation event. This is also reconcilable with the much-reduced stereoselectivity of these systems, in comparison with **1** and **2** in particular. Nonetheless, despite the similar *T*_C_ values of **4**, **5** and **7**, the increasing propensity of the larger-radius systems to adopt a homoditopic ligand conformation in the solid phase is, alongside the observed tendency for **5** and **6** to adopt different conformations dependent upon the solvent of crystallization, illustrative of the subtle differences in inversion behavior across the series. In addition to any role in influencing catalytic activity, this may be relevant to the general reduction in stereoselectivity that is observed with increasing ionic radius (Table 2 and Supporting Information), although this trend may also be attributed simply to the weaker kinetic control exerted by a less constrained active site. The degree of heterotactic enrichment of polymer products also generally decreased in line with reaction progress, with this effect being more pronounced for the largest-ionic-radius systems, **6** and **7**, suggestive of the increased proliferation of side reactions.

Unlike **1** and **2**, the observed activity of **3** conforms to the apparent linear association between the ionic radius and polymerization rate for compounds **4**–**7**. The mutual consistency of the rates exhibited by **1**, **2**, and **3** may support the suggested role of catalyst distortion (described by the O1–M–O2 angle) in determining activity, with all three systems being linear in the solid phase. However, the much lower stereoselectivity of **3** and its apparent adherence to the metal size-activity relationship established for the comparably stereoselective M­(III) species, while conceivably incidental, may alternatively suggest that the Ce­(IV)-based system shares greater mechanistic commonalities with compounds **4**–**7** than with **1** and **2**, beyond simply permitting similar proliferation of side reactions. Moreover, the ionic radii of the Zr­(IV) and Hf­(IV) centers of **1** and **2** are sufficiently small that the identified relationship would predict them to be entirely catalytically inactive under the current reaction conditions. This not being the case indicates that for smaller metals, polymerization may occur by a highly heteroselective pathway (upon which ionic radius may have little influence), with compounds of larger-radius metals able to access a distinct, more favorable, mechanistic regime, albeit affording lower stereoselectivity, to which ionic radius is of clear relevance. More specifically, we propose that under the reaction conditions, each ligand of **3**, like those of compounds **4**–**7**, may be able to independently undergo conformational inversion, whereas for **1** and **2**, inversion can only occur in a concerted process involving both closely interdigitated ligands. As suggested for **4**–**7**, such a difference between compounds **1**–**3** may be sufficiently subtle to preclude detection via VT-NMR spectroscopy while still being of mechanistic significance.

To further assess the relative catalytic profiles of compounds **1**–**7**, each was applied to the ROP of *rac*-LA under milder, more dilute conditions than those hitherto discussed ([*rac*-LA] = 1.16 mol dm^–3^ in PhCl, [*rac*-LA]:[Cat.]:[4-MeBnOH] = 500:1:3, 90 °C for 2880 min where Cat. = **1**–**3**; 60 °C for 2880 min where Cat. = **4**, **5**; 60 °C for 1200 min where Cat. = **6**, **7**; see Table S10 in the Supporting Information). While these experiments did not represent thorough kinetic studies, the reaction conversions attained in each case for the M­(III) systems were broadly consistent with the observed relative activities of those systems at 120 °C. By contrast, under such conditions, **3** exhibited much higher activity than **1** or **2** (85% conversion versus 39 and 31%, respectively), while **1** and **2** retained greater stereoselectivity than **3** (*P*_r_ = 0.81 and *P*_r_ = 0.76 versus *P*_r_ = 0.65, respectively). This apparent difference in the temperature dependence of reactions catalyzed by **1**, **2**, and **3** is further suggestive of the Ce­(IV)-catalyzed protocol being somewhat mechanistically distinct among the M­(IV) congeners.

### Lactide Polymerization under Industrially Relevant Conditions

In the commercial ROP of a stereopure LA feed, stereochemical defects of the otherwise isotactic polymer backbone may arise from epimerization activity occurring in the context of an insufficiently selective catalytic protocol, especially under demanding (high temperature, solvent-free) reaction conditions. Accordingly, given the hitherto discussed inverse relationship between reaction progress and the heterotactic enrichment of the polymer product obtained in the presence of **6** and **7**, these catalysts were each applied to the ROP of l-LA under conditions anticipated to promote side reactions; specifically a long reaction duration, high catalyst loading and absence of exogenous alcohol; [l-LA]:[Cat.]­[4-MeBnOH] = 250:1:0, 20 h, 120 °C. In both cases, significant epimerization was detected, corresponding to inversion of nearly 10% of stereocenters (see Supporting Information).

Nonetheless, on the basis of both the impressive catalytic activity of the rare earth compounds, **4**–**7**, and the proven suitability of **1** for use under industrial conditions,^2^ we applied the most active system discussed thus far, **7**, to the high-temperature solvent-free ROP of both l-LA and *rac*-LA, on a 10 g scale, using very low catalyst loadings and a comparatively high alcohol concentration (20 ppm [La] by weight, [l-LA]:[**7**]:[4-MeBnOH] = 48,250:1:100, and 52 ppm [La] by weight [l-LA]:[**7**]:[4-MeBnOH] or [*rac*-LA]:[**7**]:[4-MeBnOH] = 18,700:1:39, respectively, introduced as a solution in PhCl; 180 °C; see Table S11 in the Supporting Information). Under such conditions, high catalytic activity was observed, with almost total suppression of epimerization activity (for l-LA ROP, *P*_r_ ∼ 0, for *rac*-LA ROP, *P*_r_ ∼ 0.6). Molecular weight control was excellent (Figure S21 in the Supporting Information), indicating that initiation of polymer chains was quantitative with respect to the alcohol, despite the high concentration of that species relative to the catalyst, confirming the immortality of the kinetic regime. However, reminiscent of **1** at similar loadings and under comparable conditions,^2^ each reaction appeared to slow after an initial period of rapid propagation (where 52 ppm La was present the reaction mixture became visibly viscous after approximately 5 min for *rac*-LA ROP and 15 min for l-LA ROP), this being consistent with a stoichiometric catalyst deactivation process, although cessation of magnetic stirring due to rapidly increasing viscosity is likely also implicated. Any such deactivation may be associated with the presence of impurities in the monomer feed, which was purified only via recrystallization to maintain industrial relevance. The empirically faster rate and higher conversion attained in the case of *rac*-LA, relative to l-LA under otherwise comparable conditions, is, accordingly, likely due to small variations in monomer purity and not the modest stereoselectivity of the catalyst. Nevertheless, possible differences in the respective rheological properties of otherwise similar reaction mixtures containing significant concentrations of either isotactic P*L*LA or largely atactic poly­(d,l-lactide) should not be discounted when making empirical assessments of reaction progress based on the observed solution viscosity. While the retention of immortal kinetics, control, and selectivity under demanding conditions is promising for commercial application, optimization of catalyst loading, mixing, or monomer purity is likely necessary to afford a scalable, economic process.

### Insights into the Ligand-Assisted Activated Monomer Mechanism

The ligand-assisted activated monomer mechanism by which we have previously reported **1** to effect the ROP of lactides is predicated initially upon coordination of the co-initiator (or, in propagation, the protonated polymer chain) to the resting catalyst, via hydrogen bond formation between the protic alcohol and a phenolate group.^2^ The ROP of LA in the presence of **2**–**7** presumably proceeds by a similar mechanism to **1**, although there may be some variation in the conformational properties of the various species’ ligand systems, **1** and **2** likely being unable to adopt a highly distorted, homoditopic configuration. Informed by the structural characterization of **4**–**9**, and specifically location of the OH proton in those systems, we now surmise that the formation of such a binary catalyst-alcohol complex as that described for **1** likely involves situation of the alcohol between two phenolate groups, with the resulting interligand linkage similarly giving rise to a degree of structural distortion, and associated exposure of the opposing face of the metal center (Figure 3). Our observations both that the catalytic activity of the various M­(III) systems is directly correlated to the ionic radius of the metal center and that the rate enhancement observed in the presence of 4-MeBnOH, versus when it is absent, is most pronounced for larger metals collectively indicate that the ease of monomer approach and coordination at the metal center, likely facilitated by alcohol-mediated structural distortion, is the principal factor underpinning the relationship between metal size and polymerization activity for these systems. This remains reconcilable with the greater tendency of the largest congeners to present a highly exposed metal center by adopting a homoditopic, eclipsed configuration.

Given the opposing positions, with respect to the metal center, of the coordinated, activating alcohol and the putative lactide coordination site, nucleophilic attack in initiation or propagation presumably involves a second equivalent of the alcohol, activated through interaction with a phenolate moiety more proximate to the monomer (Scheme 4). Consistent with our observations, here and previously,^2^ it is expected that such an activation process, involving several equivalents of the alcohol relative to the catalyst, would correspond to a nonzero-order rate dependence with respect to that species, especially in the case of larger metal centers, and even when the co-initiator loading is very high. Nonetheless, it should be noted that accurately quantifying any such effect is likely to be hindered by a reduction, at or above moderate conversion, in the viscosity of reaction mixtures wherein the alcohol loading is high relative to systems containing lower concentrations of alcohol, with this having associated effects in relation to the reaction kinetics.

**Scheme 4:**
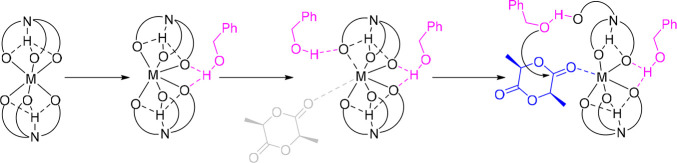
Proposed Modification of the Ligand-Assisted Activated Monomer Mechanism of ROP of LA, Catalyzed by **1**–**3** (Presumably Analogous for **4**–**7** and **10**), Comprising Catalyst Activation (Distortion), Coordination (Activation) of a Second Alcohol Equivalent (with/without the Coordinated Monomer, Shown in Gray, Present), and Nucleophilic Attack Following Phenolate Dissociation^2^

It is unclear whether, under polymerization conditions, the presence of the protonated phenolate group in the M­(III) catalysts serves to ″preactivate” them to some extent, or if that moiety is of little mechanistic consequence beyond providing insight into the likely position of the alcohol during activation. Furthermore, while the activity of **3**, relative to **4**–**7**, is compatible with this alcohol-promoted activation process applying to both M­(III) and M­(IV) systems, the absence of a significant rate difference between **3** and smaller congeners **1** and **2** at 120 °C, along with the greatly enhanced stereoselectivity and higher activation temperature(s) of the latter systems (see Table S10 in the Supporting Information), suggests that, under a given set of conditions, there may indeed be threshold ionic radii below which significant conformational variations, both in response to alcohol coordination and in relation to nonconcerted inversion of ligand chirality, respectively, become inaccessible due to excessive steric congestion. Regardless of the facility of ligand inversion, the loss of stereoselectivity is nevertheless an inevitable consequence of the reduced steric demand of a larger metal center.

### Redox Control of Polymerization Activity

Where a convenient redox couple is available, chemical or electrochemical means can be employed to access distinct forms of a single metal-based catalyst species, distinguished only by the oxidation state of the metal center. Such methods can permit direct investigation of the interaction between electronic properties and catalytic activity and selectivity.^59–61^ Various examples of such so-called ‘switchable’ systems have been reported for the ROP of lactides and other cyclic monomers, particularly based on Fe­(II)/Fe­(III),^62–66^ other transition metals,^67^ and of greater relevance to the current work, Ce­(III)/Ce­(IV) redox processes.^24,25^ Frequently, changing the oxidation state of the metal center is observed to elicit a dramatic change in the catalytic activity or selectivity of the relevant system. For example, the groups of both Okuda and Diaconescu have reported Ce-based systems for the low-temperature (40 °C and ambient temperature, respectively), solution-phase ROP of LA that exhibit greatly enhanced activity in the reduced Ce­(III) form, in comparison to those systems’ low activity and inactivity, respectively, when oxidized.^24,25^ These observations are consistent with Williams, Arnold, and co-workers’ more recent report of a Ce­(III)-based system exhibiting extraordinarily high activity in the ring-expansion polymerization of lactides.^68^ It should also be noted that similar variation in catalytic activity can be indirectly effected through the use of redox-active ligand systems, commonly those containing a ferrocenyl moiety, allowing the electronic properties of the catalyst to be manipulated while the metal center at which monomer coordination and ring-opening occurs formally remains in a single oxidation state.^63,69–73^

Although in the current work, the higher activity of the M­(III) systems, relative to the M­(IV) series, is unambiguous, it has been hitherto unclear whether this discrepancy can be attributed exclusively to the larger ionic radii of the metal centers in the former series. As discussed, the apparent irrelevance of ionic radius in determining the relative activities of the M­(IV) catalysts and the significant, and variable, structural distortions exhibited by the M­(III) systems, along with their differing degrees of catalytic activity, suggest that a more complex relationship may exist between the molecular geometry of the catalyst and the rate of ROP observed under the current conditions. Accordingly, it was desirable to prepare a trivalent system without such distortion as seen for compounds **4**–**9**, but instead resembling the highly symmetric systems **1**, **2**, and **3**. Cyclic voltammetry of Ce­(IV) compound **3** in solution in PhCl (Figure 8) confirmed that reduction of the metal center to Ce­(III) was facile, and that the magnitude of the reduction potential (*E*_1/2_ = −1.46 V, versus Fc^+^/Fc, where *E*_1/2_ = 1/2­(*E*_p,Ox_ + *E*_p,Red_)), while consistent with a highly stabilized Ce­(IV) environment,^74^ was comparable to literature values for the oxidation potential of cobaltocene, [Co­(II)­Cp_2_], in similarly nonpolar media, indicating that reduction of **3** to a Ce­(III) system in the presence of [Co­(II)­Cp_2_] may be accessible under the conditions used in the current work.^75,76^ The magnitude of the peak separation in the cyclic voltammogram (CV) of **3** suggested that, rather than a simple, reversible single-electron reduction, a more complex electrochemical event may have occurred, perhaps, for example, involving some degree of ligand reorganization.^26^ Subsequently, however, treatment of a concentrated solution of **3** with a 2-fold excess of [Co­(II)­Cp_2_] yielded a dark brown, crystalline product which was determined via single crystal X-ray diffraction to be of the cobaltocenium salt, [Co­(III)­Cp_2_]^+^[Ce­(III)­(HL^Me^)_2_]^−^, **10**, consistent with a (reversible) single-electron reduction of **3** (Figure 7). This was confirmed through further electrochemical studies (see the Supporting Information) and indicates that the atypical shape and large peak separation exhibited by the CV of **3** were likely due, in large part, to the effects of high solution resistivity and convection. The anionic Ce­(III)-based component of **10** is geometrically highly reminiscent of **1**, **2,** and **3**, being almost linear with respect to the N1–Ce1–N2 axis (N1–Ce1–N2 = 179°), and the ligands adopting a heteroditopic arrangement. Nonetheless, the mean Ce–O bond distance of **10** is markedly longer than that of **3**, rather, being intermediate between the values for the M-O distances of compounds **5** and **6**, placing it centrally in the series of M­(III) systems applied catalytically (Figure 8).

**Figure 7:**
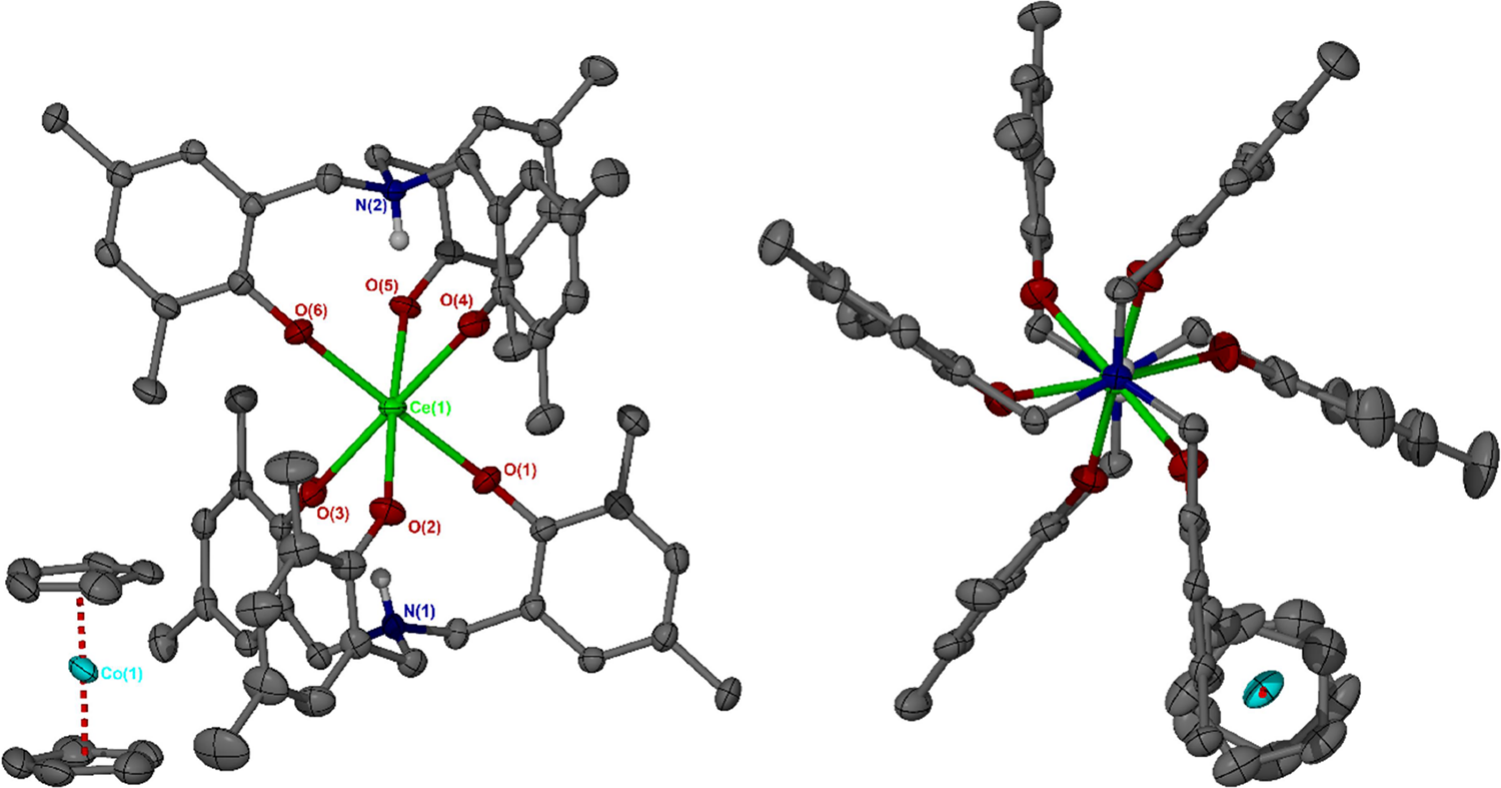
Solid-phase structure of Ce­(III) compound-containing cobaltocenium salt **10**, including the N1–Ce1–N2 axial view. Selected structural parameters are provided in Table 1. Ellipsoids are drawn at the 50% probability level. All solvent molecules, carbon-bonded hydrogen atoms, and intramolecular N–H···O interactions have been omitted for clarity.

**Figure 8:**
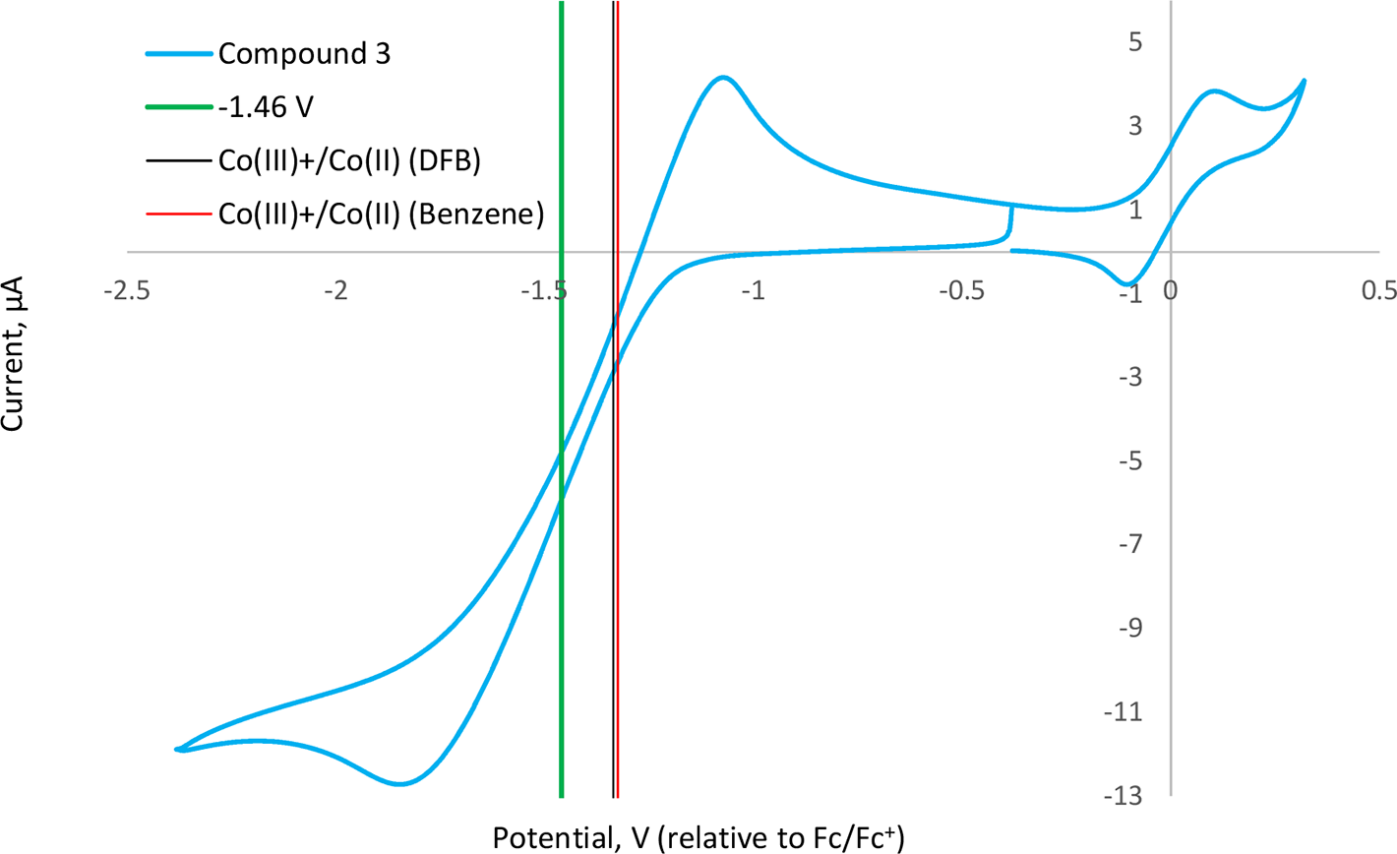
Cyclic voltammogram of **3** (approximately 20 mmol dm^–3^) in chlorobenzene solution containing an ionic liquid electrolyte (see the Supporting Information), referenced against an internal Fc/Fc^+^ standard. Scan rate = 10 mV s^–1^, using a 1.6 mm Pt working electrode. *E*_1/2_ = −1.46 V is indicated by a vertical green line. Literature values for *E*_1/2,Co(III)+/Co(II)_ in similarly nonpolar media to that described in the current work are indicated by vertical red and black lines (DFB = 1,2-difluorobenzene).^75^

Given the anticipated sensitivity of **10** toward oxidation or protonation of the anionic Ce­(III)-containing fragment, and on the basis of the precedent set by Okuda and others,^24,25^ catalytic studies proceeded via the in situ reduction of **3** in the presence of a 5-fold excess of [Co­(II)­Cp_2_]. Control experiments confirmed that neither [Co­(II)­Cp_2_] nor the cobaltocenium cation, [Co­(III)­Cp_2_]^+^ (introduced as the hexafluorophosphate salt, [Co­(III)­Cp_2_]^+^[PF_6_]^−^), was appreciably active in the ROP of *rac*-LA. In an initial attempt to acquire kinetic data for the application of **10** to the ROP of *rac*-LA at 120 °C ([*rac*-LA]:[Cat.]:[4-MeBnOH] = 1000:1:3), 61% conversion was attained in less than 2 min (this being the shortest duration assessed), with no significant increase observed for longer reaction times of up to 60 min (see Table S7 in the Supporting Information). SEC analysis of the polymer products corresponding to nine time points between 2 and 60 min revealed a narrow molecular weight distribution in all cases (*Đ*_M_ = 1.48–1.72), with little overall variation between samples, consistent with the extremely active catalyst having undergone deactivation prior to equilibrium conversion being reached, and corresponding to a TON of 610. It is plausible that in the case of an oxidative deactivation process, such a low TON could be remedied by the addition of a larger excess of [Co­(II)­Cp_2_], but this approach is undesirable due to the high cost and significant toxicity of this additive. Furthermore, the complete deactivation observed is inconsistent with oxidation of the anionic fragment of **10** to return to **3**. Given the high activity of **10**, direct comparison with **1**–**7** at 120 °C was not feasible. Thus, comparison of **10** and highly active La­(III) compound **7** was undertaken at 80 °C (see Table S8 in the Supporting Information), and the catalyst loading was increased such that [*rac*-LA]:[Cat.]:[4-MeBnOH] = 1000:2:3, with [Co­(II)­Cp_2_] remaining in 5-fold excess with respect to Ce, to compensate for any potential oxidative deactivation of the catalyst. Under such conditions, the activity of **10** exceeded that of **7** by a factor of 5 (*k*_obs_ = 1.04 h^–1^ *versus k*_obs_ = 5.36 h^–1^, see Table 3, Figure 9) and the reaction proceeded to equilibrium conversion in less than 1 h, whereas **7** required 3 h to attain comparable conversion. Consistent with all other systems discussed in this work, the molecular weight of the polymer produced in the presence of **10** was well controlled, increasing linearly with conversion.

**Figure 9:**
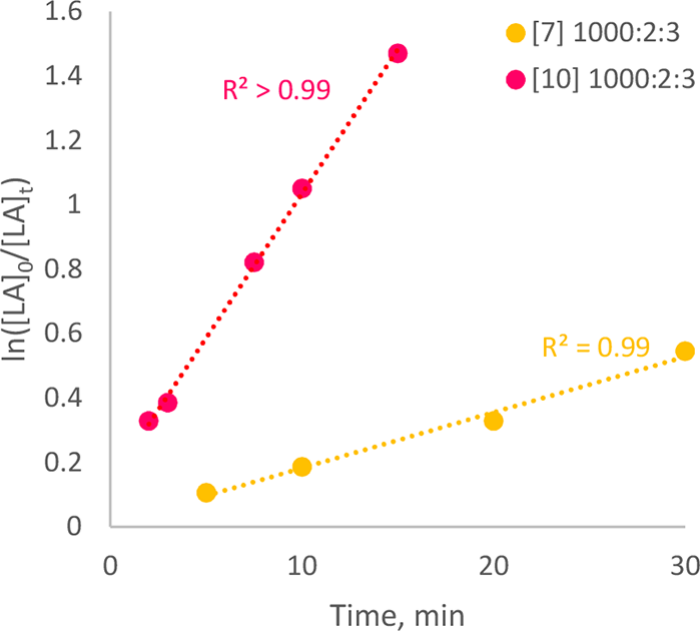
Semilogarithmic initial rate plots for the ROP of *rac*-LA in the presence of 0.2 mol % of catalysts **7** and **10** (corresponding to La­(III) and Ce­(III)) and 0.3 mol % 4-MeBnOH, at 80 °C in anhydrous PhCl.

While not necessarily a precise quantitative method due to possible differences in the temperature dependence of the two systems, multiplication of the 5-fold activity ratio of **10** and **7** at 80 °C with the 24.7-times rate difference between **7** and Ce­(IV) compound **3** at 120 °C (*k*_obs_ = 8.90 h^–1^ versus *k*_obs_ = 0.360 h^–1^, see Table 4) suggests that reduction of **3** to **10** elicits an approximately 125-fold increase in catalytic activity. Additionally, comparison of data corresponding to **3** and **10**, at 120 °C, the activity of the latter being conservatively deduced based on the attainment of 61% conversion in 2 min, provided a lower bound of 89 for the coefficient by which the catalytic activity of **3** is increased on reduction of the Ce­(IV) center. However, this value is likely to be to be a significant underestimate, as it does not account for the increasing significance at very short reaction durations of the time taken for the reaction mixture to be heated to a stable temperature, the possibility that, in the case of **10**, 61% conversion was attained (and the catalyst deactivation complete) in substantially less than 2 min, or that catalyst deactivation may have occurred progressively during the reaction.

In addition to this work revealing a higher activity ratio than has been reported by Okuda and co-workers for Ce­(III)/Ce­(IV) systems,^24^ and demonstrating for the first time redox control of a Ce-based ROP catalyst under challenging, near-melt conditions, **10** is also much more active than would be expected, relative to compounds **4**–**7**, on the basis of ionic radius (mean M–O bond distance) alone. This is in contrast to the apparent agreement between the observed activity of Ce­(IV) compound **3** and the corresponding predicted value. Okuda and co-workers previously attributed the 60-fold rate enhancement they observed on reduction of an OSSO-type bis­(phenolato) Ce­(IV)-based catalyst to the concomitant increase in the ionic radius of the Ce center. Consistent with previous reports,^24,25^ our findings indicate that the high activity of the current Ce­(III) system may be attributable to factors more complex than the size of the metal center alone, and that the polymerization mechanism associated with the large, symmetric systems **3** and (the anion of) **10** may be somewhat distinct from those favored by both the neutral M­(III) and M­(IV) systems herein, exhibiting a more pronounced rate dependency with respect to ionic radius. For example, given the uncertain mechanistic significance of the phenolate proton in the catalytic application of **4**–**7**, its absence in **10** may indeed enhance the overall conformational flexibility of the system, increasing the facility of activation in the presence of alcohol. However, the clear distinction between the electronic properties of this anionic system and those, variously, of **1**–**7** currently precludes a more definitive explanation of its remarkable catalytic activity. Certainly the greater magnitude of the Ce­(III)/Ce­(IV) activity ratio for the current system, in comparison to those reported by others, especially given the near-identical proportional increase in ionic radius associated with reduction of the Ce­(IV) center for our system relative to that reported by Okuda and co-workers (6.5% versus 6.0% increase in mean crystallographic Ce–O bond distance, respectively),^24^ indicates that the magnitude of this effect is dependent upon the specific nature of the catalytic system. Although not attempted in this work, synthesis and catalytic studies of a neutral, protonated Ce­(III) species, analogous to compounds **4**–**7**, derived from a suitable Ce­(III) precursor, may be expected to further illuminate the deviation of **10** from the trend observed for other trivalent systems.

**10** evidently represents a remarkably active catalyst system, with the observed redox-facilitated activity enhancement under demanding conditions feasibly providing opportunities in relation to electrochemically mediated polymer production, reprocessing, and recycling, specifically via the in situ activation of an otherwise indefinitely air- and moisture-stable compound, **3**. However, our observations that the activity of zwitterionic compounds of the type discussed in this work can be enhanced on the addition of further equivalents of exogenous alcohol suggest that even higher absolute rates may be readily attainable without variation of the catalyst loading, albeit with a concurrent reduction in the polymer molecular weight. This may be of greater relevance to the more robust neutral M­(III) compounds, with the combination of very high activity, stability, and absence of discernible color making **7**, in particular, an interesting system in the context of industrial catalysis. Accordingly, future studies with this system should focus further on optimizing its application under industrially relevant, high temperature, solvent-free conditions, with low catalyst loadings and high [alcohol]:[metal] ratio.

## Conclusions

While zwitterionic metal compounds derived from pro-ligand H_3_L^Me^ are of proven industrial relevance for the ROP of lactide(s),^2^ the current work reveals that straightforward adaptation of the simple synthetic method used to prepare the prototypical Zr­(IV) compound, **1**, to alternative metal alkoxide precursors can yield a wide range of closely related, catalytically active species. Kinetic studies have demonstrated that variation of both the ionic radius and the oxidation state of the metal center can dramatically alter the rate and selectivity of polymerization. Significant rate enhancements, relative to **1**, were observed in nearly all cases, while the polymerizations consistently remained well-controlled and the catalysts exhibited reasonable robustness. Compounds of trivalent rare earth elements Yb­(III), Y­(III), Pr­(III), and La­(III) were all significantly more active than **1**, exhibiting an unambiguous relationship between ionic radius and rate in the ROP of *rac*-LA, albeit with a concurrent reduction in stereoselectivity. We suggest that these trends may specifically find their origin in the more distorted molecular geometry arising in the presence of larger M­(III) metal centers relative to the highly symmetric conformation of the various M­(IV)-based species. Reconciling these findings with the previously described ligand-assisted activated monomer mechanism reveals that an analogous distortion may underlie the rate enhancement observed in response to increasing the concentration of alcohol in the ROP of LA mediated by **1**, with similar relevance to the systems reported herein.^2^ Whereas compounds of the tetravalent metals Zr­(IV), Hf­(IV), and Ce­(IV) did not exhibit a clear relationship between ionic radius and catalytic activity, reduction of the latter species by treatment with excess cobaltocene to afford a structurally analogous Ce­(III) compound increased catalytic activity by 2 orders of magnitude. Ongoing foci for research in this area include optimizing application of the most active neutral M­(III)-based catalysts (e.g., lanthanum compound **7**) to the industrially relevant high-temperature, solvent-free ROP of lactides, and developing robust, efficient electrochemical methods for in situ reduction of Ce­(IV) compound **3** to the anionic Ce­(III) congener reported herein as the anionic fragment of cobaltocenium salt **10**, to provide practical, switchable catalytic processes. As such, we aim to advance polymerization and depolymerization technologies for the production of renewable and sustainable materials.

## Supplementary Material



## Notes

Complete synthetic procedures and characterization data for compounds **1**–**10**, including VT-NMR data for compounds **1**–**7** and electrochemical data for compound **3**; polymerization methods and kinetic data, and complete polymer characterization data, including all SEC traces acquired; crystallographic parameters and CSD deposition numbers for all solid-phase structures (compounds **2**–**10**); and additional figures of solid phase structures (PDF)
