# Supplementary material for: Diversity in Zwitterionic Metal Ammonium Tris(phenolate)s for the Controlled Immortal Polymerization of Lactide: Dramatic Activity Enhancement and Mechanistic Insight on Expansion beyond Zirconium
Source: ACS Catal. 2025 May 14;15(11):9130–49. doi: 10.1021/acscatal.5c01857 (PMC12150268; doi:10.1021/acscatal.5c01857)
Supplement: Supplementary file 1 [file cs5c01857_si_001.pdf]

Supporting Information

Diversity in zwitterionic metal ammonium tris(phenolate)s for the controlled immortal polymerization of lactide: dramatic activity enhancement and mechanistic insight on expansion beyond zirconium

Matthew G. Davidson*,[a,b] Catherine J. Frankis,[b] Matthew D. Jones,[a,b] Gabriele Kociok-Köhn,[b,c] Frank Marken,[b] Strachan N. McCormick*,[a,b] James Tipler,[b] and Philip B. Yang.[a,b]

[a] Institute of Sustainability and Climate Change, University of Bath, Bath BA2 7AY, United Kingdom

[b] Department of Chemistry, University of Bath, Bath BA2 7AY, United Kingdom

[c] Core Research Facilities (CRF), University of Bath, Bath BA2 7AY, United Kingdom

E-mail: S.N.McCormick@bath.ac.uk, M.G.Davidson@bath.ac.uk

Contents

S1. General considerations 3

S2. Synthetic procedures and analytical data 6

Synthesis of pro-ligand tris(2-hydroxy-3,5-dimethylbenzyl)amine, H_3L^{Me} 6

Synthesis of $[Zr(HL^{Me})_2]$, **1** 6

Synthesis of $[Hf(HL^{Me})_2]$, **2** 6

Synthesis of $[Ce(HL^{Me})_2]$, **3** 7

Synthesis of $[Yb(HL^{Me})(H_2L^{Me})]$, **4** 8

Synthesis of $[Y(HL^{Me})(H_2L^{Me})]$, **5** 9

Synthesis of $[Pr(HL^{Me})(H_2L^{Me})]$, **6** 10

Synthesis of $[La(HL^{Me})(H_2L^{Me})]$, **7** 11

Synthesis of $[Sc(HL^{Me})(H_2L^{Me})]$, **8** 11

Synthesis of $[Sm(HL^{Me})(H_2L^{Me})]$, **9** 12

Synthesis of $[Co(III)Cp_2]^+[Ce(III)(HL^{Me})_2]^-$, **10** 12

S3. Variable-temperature ^{1}H NMR spectroscopic studies of compounds 1 – 7 13

S4. Electrochemical characterisation of Compound 3 22

S5. Polymerisation methods 24

S6. Polymerisation data tables 26

S7. Kinetic data and associated structural and polymer characterisation data 34

S8. Size Exclusion Chromatograms 39

S9. Sample homonuclear decoupled ^{1}H NMR spectrum of poly(*D*,*L*-LA) 55

S10. Crystallographic parameters 56

S11. Additional crystal structure figures 58

References 60

S1. General considerations

Manipulations, unless otherwise specified, were carried out under an atmosphere of dry argon using standard Schlenk line and glove box techniques. An MBraun glove box was used, with an atmosphere typically maintained with O_2 and H_2O concentrations <0.1 ppm and <0.1 ppm, respectively. Argon was supplied by BOC and used without further purification. Polymerisation reactions were, unless otherwise specified, undertaken under a dry argon atmosphere in 4 ml borosilicate glass vials, with PTFE-lined melamine caps, under magnetic stirring.

All NMR spectra were acquired using a 400 MHz (^{1}H), 101 MHz (^{13}C), or 500 MHz (^{1}H), 126 MHz (^{13}C), Bruker Avance spectrometer. NMR data were processed using MestReNova Version 11.0.1-17801 (2016) from Mestrelab Research S.L. Spectra were referenced and reported relative to residual solvent resonances.

Polymer molecular weight data was acquired using an Agilent 1260 Size Exclusion Chromatography (SEC) system with refractive index detector, calibrated against polystyrene standards of known molecular weight. A PLgel 5 µm MIXED-D 300 x 7.5 mm column was used, with a PLgel 5 µm MIXED Guard 50 x 7.5 mm guard column. The mobile phase was THF, at a flow rate of 1 ml min^{-1}. Columns and detectors were maintained at 35 °C. Data was processed using Agilent's GPC/SEC Software, Revision A.02.01. Samples were typically prepared with an analyte concentration of 2 mg ml^{-1} and filtered through a 0.2 µm PTFE syringe filter prior to analysis.

Crystallographic intensity data for structures of **2**, **3**, **4** (with Chlorobenzene in the unit cell), **5** (with Toluene and Chlorobenzene in the unit cell), **6** (with Toluene in the unit cell), **7** (with Toluene in the unit cell) and **10** were collected at 150(2) K on a Rigaku SuperNova dual-source X-ray diffractometer with EosS2 detector, using Cu-Kα radiation (λ= 1.54184 Å). Intensity data for **4** (with $CDCl_3$ in the unit cell), **5** (with $CDCl_3$ in the unit cell) and **6** (with $CDCl_3$ in the unit cell), were collected using a XtaLAB Synergy, Dualflex diffractometer with HyPix-Arc 100 detector, using a Cu microfocus X-ray source (Cu-Kα, λ = 1.54184 Å). Intensity data for **7** (with $CDCl_3$ in the unit cell) were collected using a Rigaku New Xcalibur diffractometer with EosS2 detector, using graphite monochromated Mo-Kα radiation (λ = 0.71073 Å). Intensity data for **8** and **9** were collected using a Nonius Kappa CCD single crystal diffractometer using graphite monochromated Mo-Kα radiation (λ = 0.71073 Å).

Unit cell determination, data collection, data reduction and a symmetry-related (multi-scan) absorption correction were performed using the CrysAlisPro software (CrysAlisPro 1.171.43.104 (Rigaku OD, 2024)) or the Nonius Software.[1]

Structures were solved with SHELXT and refined by a full-matrix least-squares procedure based on F^2 (Shelxl-2019/3).[2]

All non-hydrogen atoms were refined anisotropically. Hydrogen atoms were placed onto calculated positions and refined using a riding model.

Additional programmes used for analysing data and their graphical manipulation included SHELXle,[3] and Mercury.[4]

Accurate mass spectrometry analyses were conducted using a MaXis HD quadrupole electrospray time-of-flight (ESI-QTOF) mass spectrometer (Bruker Daltonik GmbH, Bremen, Germany), using a glass syringe (Hamilton) and syringe pump (KD Scientific, Model 781100) for infusions at a flow rate of 3 µL/min. Analyses were performed in ESI positive mode with the capillary voltage was set to 4500 V, nebulizing gas at 1 bar, drying gas at 6 L/min at 180°C in each case. The TOF scan range was from 50 – 1000 mass-to-charge ratio (*m/z*). The MS instrument was calibrated using an infusion of sodium

formate calibrant solution. The calibrant solution consisted of 3 parts of 1 M NaOH to 97 parts of 50:50 water:isopropanol with 2% formic acid. Sample solutions were prepared in toluene. Data processing was performed using the Compass Data Analysis software version 4.3 (Bruker Daltonik GmbH, Bremen, Germany).

Elemental (CHN) combustion analyses of compounds **2** – **7** were undertaken by ElementalLab (Elemental Microanalysis Ltd.), UK. Samples were prepared under an inert argon atmosphere before transfer to evacuated (<0.1 mBar), flame-sealed borosilicate glass ampoules before submission. Samples were handled under inert atmosphere and analysed in duplicate using Dumas combustion method, under conditions conforming to ISO 17025. Reported values represent the mean calculated from the two analyses. Elemental (CHN) analyses of **8**, **9** were undertaken by Mr. Stephen Boyer of London Metropolitan University.

Electrochemical studies (cyclic voltammetry) were undertaken in a glove box under an inert argon atmosphere, an electrolyte solution of trihexyl(tetradecyl)phosphonium tris(pentafluoroethyl)trifluorophosphate in anhydrous chlorobenzene, and using the following electrodes: 1.6 mm diameter Pt disk working electrode, Pt wire reference electrode, and glassy carbon counter electrode. Ferrocene was used as an internal standard against which potential values were referenced. For initial determination of the reduction potential of compound **3**, a scan rate of 10 mV s^{-1} was used (see below). Data were collected using an Emstat3 potentiostat with bluetooth control (Palmsense), and processed using the PSTrace4.2 software, and Microsoft Excel.

Zirconium(IV) isopropoxide isopropanol complex and cerium(IV) 2-methoxyethoxide (18-20% wt/wt solution in 2-methoxyethanol) were purchased from ThermoFisher and used without further purification. Ytterbium(III) isopropoxide was purchased from ThermoFisher, and purified before use to remove insoluble impurities, by dissolution in anhydrous dichloromethane and passage through a 0.2 µm PTFE syringe filter. Hafnium(IV) isopropoxide isopropanol complex, yttrium(III) isopropoxide (20 – 25% wt/wt solution in toluene) and cobaltocenium hexafluorophosphate were purchased from Strem Chemicals and used without further purification. Praseodymium(III) isopropoxide was purchased from Strem Chemicals and, where necessary, purified before use to remove insoluble impurities, by dissolution in anhydrous dichloromethane and passage through a 0.2 µm PTFE syringe filter. Samarium(III) isopropoxide, scandium(III) isopropoxide (25% wt/vol solution in isopropanol) and cobaltocene were purchased from Sigma Aldrich and used without further purification. Lanthanum(III) isopropoxide was purchased from Sigma Aldrich and purified before use to remove insoluble impurities, by dissolution in anhydrous dichloromethane and passage through a 0.2 µm PTFE syringe filter. Hexamethylenetetramine and paraformaldehyde were purchased from Sigma Aldrich and used without further purification. 2,4-dimethylphenol was purchased variously from Sigma Aldrich or ThermoFisher and used without further purification.

Chloroform-*d* used for analysis of polymer samples was purchased from Fluorochem (UK) and used without further purification. Anhydrous chloroform-*d* was purchased from Sigma Aldrich and used without further purification, or dried over 4 Å molecular sieves and degassed using the freeze-pump-thaw method (a minimum of four such cycles). Anhydrous dichloromethane-d_2 was purchased from Sigma Aldrich and dried over calcium hydride before being vacuum distilled and stored over 4 Å molecular sieves. Anhydrous chlorobenzene, anhydrous dichloromethane, anhydrous toluene for synthesis and analysis of catalysts **1** – **7**, anhydrous hexanes (mixture of isomers), methanol and ethyl acetate were purchased from Sigma Aldrich and used without further purification. Anhydrous toluene and dichloromethane for synthesis of **8**, **9** were drawn from an MBraun Solvent Purification System. Samples for SEC analysis were prepared in "GPC-grade" tetrahydrofuran, purchased from ThermoFisher. Anhydrous toluene for recrystallisation of *rac*-lactide was drawn (under air) from an

MBraun Solvent Purification System. Anhydrous toluene for recrystallisation of *L*-lactide was purchased from Sigma Aldrich and used without further purification. 4-methylbenzyl alcohol was purchased from Sigma Aldrich and used without further purification. APCI tuning solution, for calibration of the mass spectrometer was purchased from Sigma Aldrich and used without further purification. Trihexyl(tetradecyl)phosphonium tris(pentafluoroethyl)trifluorophosphate (electrolyte) and ferrocene for electrochemical analysis (cyclic voltammetry) were purchased from Sigma Aldrich and used without further purification. *rac*-lactide and *L*-lactide were supplied free of charge by TotalEnergies Corbion.

S2. Synthetic procedures and analytical data

Synthesis of pro-ligand tris(2-hydroxy-3,5-dimethylbenzyl)amine, H_3L^{Me}

H_3L^{Me} was prepared according to the adapted literature procedure we have employed previously.[5,6] ^1H NMR spectroscopic analysis, and comparison with the literature, confirmed the identity and purity of the product. H_3L^{Me} was dried under dynamic vacuum overnight prior to use under an inert argon atmosphere.

*Synthesis of [Zr(HL^{Me})$_2$], **1***

1 was synthesised according to the following adapted literature procedure:[7,8] In a Schlenk tube, in the glove box, were combined [Zr(O^{i}Pr)$_4$·HO^{i}Pr] (2.5 g, 6.45 mmol, 1 equiv.) and H_3L^{Me} (5.4 g, 12.88 mmol, 2 equiv.). ~20 ml of dry toluene was added to the combined solids, yielding a white suspension. After stirring the mixture for 20 hours at ambient temperature, all residual solid material was heated into solution (hot air gun), and allowed to re-precipitate on cooling to ambient temperature. The solution phase was then removed by cannula filtration, and the resulting solid product dried under dynamic vacuum.

Yield: 5.26 g, 88%

A further quantity of **1** was isolated from the supernatant by evaporation of solvent under ambient air, followed by washing with toluene.

Yield: 0.60 g, 10%

^1H and ^{13}C NMR spectroscopic analyses were consistent with the literature.

*Synthesis of [Hf(HL^{Me})$_2$], **2***

2 was synthesised according to the following adapted literature procedure:[7,8] Using identical methodology to that described for **1**, above, 2.5 g [Hf(O^{i}Pr)$_4$·HO^{i}Pr] (2.5 g, 5.26 mmol, 1 equiv.) was reacted with H_3L^{Me} (4.42 g, 10.53 mmol, 2 equiv.).

Yield: 4.80 g, 90%

Crystals suitable for X-ray diffraction were obtained by concentration of the supernatant after isolation of the bulk product *via* cannula filtration.

^1H NMR (500.13 MHz, Chloroform-*d*, 298 K, δ_H, ppm); 11.79 (2H, *t*, *broad*, *J* = 5.0 Hz, N*H*), 6.85 (6H, *d*, *J* = 1.9 Hz, *Ar*), 6.67 (6H, *d*, *J* = 1.9 Hz, *Ar*), 4.79 (6H, *d*, *broad*, *J* = 10.5 Hz, NCH*H*), 3.31 (6H, *t*, *broad*, *J* = 10.5 Hz, NC*H*H), 2.18 (18H, *s*, ArC*H$_3$*), 1.93 (18H, *s*, ArC*H$_3$*). **$^{13}C\{^1H\}$ NMR** (125.77 MHz, Chloroform-*d*, 298 K δ_c, ppm); 159.39 (*Ar*), 132.92 (*Ar*H), 129.81 (*Ar*), 128.10 (*Ar*H), 125.02 (*Ar*), 117.20 (*Ar*), 56.31 (N*C*H$_2$), 20.51 (*C*H$_3$), 16.65 (*C*H$_3$). **Elemental (CHN) Analysis** Calculated, for $C_{54}H_{62}HfN_2O_6$; C: 63.99%, H: 6.17%, N: 2.71%. Experimental; C: 63.71%, H: 6.17%, N: 2.72%.

Synthesis of $[Ce(HL^{Me})_2]$, ***3***

3 was synthesised according to the following adapted literature procedure:[7,8] In the glove box, a Schlenk tube was charged with 2.64 g H_3L^{Me} (6.29 mmol, 2 equiv.), to which 30 ml Toluene was added, to yield a white suspension. Then, ~1.39 g $[Ce(O(CH_2)_2OCH_3)_4]$ (3.15 mmol, 1 equiv.) (as 7.3 g 18-20% wt/wt solution in 2-methoxyethanol, $HO(CH_2)_2OCH_3$) was added dropwise over the course of approximately 30 seconds, upon which the solution was observed immediately to turn dark brown. The solution was stirred for 20 hours, by which time a brown crystalline solid had formed. The solid was isolated by cannula filtration and dried under dynamic vacuum.

Crude yield: 2.20 g, 72%

The crude product was dissolved in anhydrous Chlorobenzene in the glove box, and passed through a 0.2 µm PTFE syringe filter, before removal of volatiles *in vacuo* for 20 hours at 60°C.

Yield: 0.5 g, 16%

Crystals suitable for X-ray diffraction were obtained by slow evaporation, in the glove box, of dichloromethane from a solution of **3** in dichloromethane and toluene.

^{1}H NMR (500.13 MHz, Chloroform-*d*, 298 K, δ_H, ppm); 11.52 (2H, *t*, *broad*, *J* = 5.7 Hz, N*H*), 6.90 (6H, *d*, *J* = 1.9 Hz, *Ar*), 6.67 (6H, *d*, *J* = 1.9 Hz, *Ar*), 4.87 (6H, *broad*, NCH*H*), 3.24 (6H, *broad*, NC*H*H), 2.25 (18H, *s*, ArC*H₃*), 2.19 (18H, *s*, ArC*H₃*). **^{13}C{^{1}H} NMR** (125.77 MHz, Chloroform-*d*, 298 K δ_c, ppm); 166.27 (*Ar*), 132.27 (*Ar*H), 129.48 (*Ar*), 127.77 (*Ar*H), 125.54 (*Ar*), 116.20 (*Ar*), 56.70 (N*C*H_2), 20.45 (*C*H_3), 16.86 (*C*H_3). **Elemental (CHN) Analysis** Calculated, for $C_{54}H_{62}CeN_2O_6$; C: 66.51%, H: 6.41%, N: 2.87%. Experimental; C: 66.76%, H: 6.25%, N: 2.89%.

Synthesis of [Yb(HL^{Me})(H$_2$L^{Me})], ***4***

In the glove box, a 27 ml glass vial was charged with 1 g [Yb(O^{i}Pr)$_3$] (mmol, equiv.), to which ~25 ml anhydrous dichloromethane was added, partially dissolving the [Yb(O^{i}Pr)$_3$]. The dense residual solid material was allowed to settle, and the turbid solution phase removed using a syringe and hypodermic needle, before being passed through a 0.2 µm PTFE syringe filter into a Schlenk flask. The solid was dried under dynamic vacuum (glove box antechamber) and weighed. 350 mg of residue was obtained. A further 400 mg [Yb(O^{i}Pr)$_3$] was dissolved in anhydrous dichloromethane and syringe-filtered into the same Schlenk flask, thus adding approximately 260 mg of soluble material, to afford a combined total of 910 mg [Yb(O^{i}Pr)$_3$] (2.60 mmol, 1 equiv., assuming absence of soluble impurities) in 50 ml anhydrous dichloromethane. To this was added 2.40 g H$_3$L^{Me} (5.72 mmol, 2.2 equiv.), followed by 60 ml anhydrous toluene. After stirring at ambient temperature for 16 hours, the volume of the resulting solution was reduced under dynamic vacuum to approximately 20 ml, which was anticipated to result in a primarily toluene-containing solvent system. A white solid immediately began to precipitate, and this was warmed back into solution (hot air gun). On standing for 20 hours the product precipitated, and was isolated *via* cannula filtration before being dried overnight (20 hours) under dynamic vacuum at 65°C.

Yield: 2.30 g, 88%

Crystals suitable for X-ray diffraction were obtained by slow evaporation, in the glove box, of dichloromethane from a solution of **4** in dichloromethane and chlorobenzene, and by slow evaporation of chloroform-*d* from a solution of **4** in that solvent, respectively.

Paramagnetic sample; ^{1}H NMR (500.13 MHz, Chloroform-*d*, 298 K, δ_H, ppm); 6.86 (6H, *s*, *Ar*), 6.71 (6H, *s*, *Ar*), 3.55 (12H, *broad*, NC*H$_2$*), 2.21 (36H, *s*, ArC*H$_3$*). **^{13}C{^{1}H} NMR** (125.77 MHz, Chloroform-*d*, 298 K δ_c, ppm); 151.19 (*Ar*), 131.42 (*Ar*H), 129.25 (*Ar*H), 129.01 (*Ar*), 124.67 (*Ar*), 121.88 (*Ar*), 56.58 (N*C*H$_2$), 20.54 (*C*H$_3$), 16.03 (*C*H$_3$). **Elemental (CHN) Analysis** Calculated, for $C_{54}H_{63}YbN_2O_6$; C: 64.27%, H: 6.29%, N: 2.78%. Experimental; C: 64.95%, H: 6.39%, N: 2.79%.

ESI-MS (m/z): 1010.4227; calc. for $[C_{54}H_{64}YbN_2O_6]^+$ (***4*** *+ H$^+$*): 1010.4148.

(m/z): 1032.4054; calc. for $[C_{54}H_{63}YbN_2NaO_6]^+$ (***4*** *+ Na$^+$*): 1032.3967.

*Synthesis of [Y(HL^{Me})(H$_2$L^{Me})], **5***

In the glove box, ~2.1 g [Y(O^{i}Pr)$_3$] (7.89 mmol, 1 equiv.) (as 7.4 g of 20 – 25% wt/wt solution in toluene) was added to a Schlenk tube containing a suspension of 6.3 g (15 mmol, 1.9 equiv.) H$_3$L^{Me} in ~50 ml dichloromethane. The resulting mixture was stirred for 24 hours at ambient temperature, by which time an off-white solid had precipitated. The solid product was isolated by cannula filtration and dried under dynamic vacuum for 20 hours.

Yield: 2.20 g, 32%

Crystals suitable for X-ray diffraction were obtained by addition of toluene to a solution of **5** in chlorobenzene, and by slow evaporation of chloroform-*d* from a solution of **5** in that solvent, respectively.

^{1}H NMR (500.13 MHz, Dichloromethane-*d$_2$*, 298 K, δ_H, ppm); 11.50 (2H, *broad*, N*H*), 6.87 (6H, *s*, *Ar*), 6.71 (6H, *s*, *Ar*), 3.99 (12H, *broad*, NC*H$_2$*), 2.18 (18H, *s*, ArC*H$_3$*), 1.98 (18H, *s*, ArC*H$_3$*). **^{13}C{^{1}H} NMR** (125.77 MHz, Chloroform-*d*, 298 K δ_c, ppm); 159.06 (*Ar*), 132.81 (*Ar*H), 129.11 (*Ar*H), 127.82 (*Ar*), 125.06 (*Ar*), 117.06 (*Ar*), 56.84 (N*C*H$_2$), 20.44 (*C*H$_3$), 16.85 (*C*H$_3$). **Elemental (CHN) Analysis** Calculated, for $C_{54}H_{63}YN_2O_6$; C: 70.12%, H: 6.87%, N: 3.03%. Experimental; C: 69.96%, H: 6.78%, N: 3.00%.

ESI-MS (m/z): 925.3914; calc. for $[C_{54}H_{64}YN_2O_6]^+$ (***5** + H$^+$*): 925.3817.

(m/z): 947.3730; calc. for $[C_{54}H_{63}YN_2NaO_6]^+$ (***5** + Na$^+$*): 947.3637.

Synthesis of [Pr(HL^{Me})(H$_2$L^{Me})], ***6***

To a Schlenk tube, in the glove box, was added 0.98 g [Pr(O^{i}Pr)$_3$] (3.08 mmol, 1 equiv.) and ~25 ml anhydrous dichloromethane (initially 1.06 g of [Pr(O^{i}Pr)$_3$] was weighed, but when dissolved in dichloromethane, approximately 80 mg of insoluble residue remained, see synthesis of **4**, above). This was combined with 2.64 g H_3L^{Me} (6.29 mmol, 2 equiv.), and 50 ml anhydrous toluene added. The resulting solution was stirred for 24 hours at ambient temperature, then the volume reduced by ~50% under dynamic vacuum (anticipated to result in primarily a toluene-based solvent system), whereupon a white precipitate formed. The solid material was isolated by cannula filtration, then washed with a further aliquot (~20 ml) of anhydrous toluene, which was removed by cannula filtration. The solid material was then dried under dynamic vacuum before being dissolved, in the glove box, in ~15 ml chlorobenzene, and the resulting solution passed through a 0.2 µm PTFE syringe filter, before removal of volatiles and drying for 20 hours at 75 ˚C under dynamic vacuum. Although the bulk material obtained was soluble in chloroform-*d* and dichloromethane, and despite the thorough purification protocol employed, such solutions were consistently observed to be slightly turbid. Such turbidity consistently remained after passage through a 0.2 µm PTFE syringe filter. Elemental (CHN) analysis data were consistent with the presence of ~1 – 1.5 wt% insoluble Pr_2O_3 (see below).

Yield: 2.45 g, 81%

Crystals suitable for X-ray diffraction were obtained by slow evaporation, in the glove box, of dichloromethane from a solution of **6** in anhydrous dichloromethane and anhydrous toluene, and by slow evaporation of chloroform-*d* from a solution of **6** in that solvent, respectively.

Highly paramagnetic sample. **Elemental (CHN) Analysis** Calculated, for $C_{54}H_{63}PrN_2O_6$; C: 66.39%, H: 6.50%, N: 2.87%. Experimental; C: 65.34%, H: 6.42%, N: 2.84%.

ESI-MS (m/z): 977.3935; calc. for $[C_{54}H_{64}PrN_2O_6]^+$ (***6*** *+ H*$^+$): 977.3835.

(m/z): 999.3759; calc. for $[C_{54}H_{63}PrN_2NaO_6]^+$ (***6*** *+ Na*$^+$): 999.3655.

*Synthesis of [La(HL^{Me})(H$_2$L^{Me})], **7***

To a Schlenk tube, in the glove box, was added 0.75 g [Pr(O^{i}Pr)$_3$] (2.37 mmol, 1 equiv.) and ~25 ml anhydrous dichloromethane (initially 0.75 g of [Pr(O^{i}Pr)$_3$] was weighed, but when dissolved in dichloromethane, approximately 50 mg of insoluble residue remained, see syntheses of **4**, **5**, above. A further 50 mg of [Pr(O^{i}Pr)$_3$] was therefore dissolved, to the maximum possible extent, in anhydrous dichloromethane, passed through a 0.2 µm PTFE syringe filter, and added to the reaction mixture). This was combined with 2.00 g H_3L^{Me} (4.77 mmol, 2 equiv.), and ~30 ml anhydrous toluene added. After stirring for 20 hours at ambient temperature, the volume of the solution was reduced by ~50% under dynamic vacuum (anticipated to result in a primarily toluene-based solvent system), and the product formed, as a colourless crystalline solid, from the solution on standing.

Yield: 2.07 g, 90%

Crystals suitable for X-ray diffraction were taken directly from the reaction vessel, and isolated by slow evaporation in the glove box of chloroform-*d* from a solution of **7** in that solvent, respectively.

Paramagnetic sample; ^{1}H NMR (500.13 MHz, Chloroform-*d*, 298 K, δ_H, ppm); 10.69 (2H, *broad*, N*H*), 6.88 (6H, *s*, *Ar*), 6.71 (6H, *s*, *Ar*), 3.88 (12H, *broad*, NC*H$_2$*), 2.20 (18H, *s*, ArC*H$_3$*), 2.03 (18H, *s*, ArC*H$_3$*). **^{13}C{^{1}H} NMR** (125.77 MHz, Chloroform-*d*, 298 K δ_c, ppm); 159.04 (*Ar*), 132.37 (*Ar*H), 129.11 (*Ar*H), 127.01 (*Ar*), 125.03 (*Ar*), 117.72 (*Ar*), 57.58 (N*C*H$_2$), 20.51 (*C*H$_3$), 17.23 (*C*H$_3$). **Elemental (CHN) Analysis** Calculated, for $C_{54}H_{63}LaN_2O_6$; C: 66.52%, H: 6.51%, N: 2.87%. Experimental; C: 66.31%, H: 6.53%, N: 2.81%.

ESI-MS (m/z): 975.3907; calc. for $[C_{54}H_{64}LaN_2O_6]^+$ (***4** + H$^+$*): 975.3822.

(m/z): 997.3758; calc. for $[C_{54}H_{63}LaN_2NaO_6]^+$ (***4** + Na$^+$*): 997.3642.

*Synthesis of [Sc(HL^{Me})(H$_2$L^{Me})], **8***

In a fritted Schlenk tube, 1.28 g H_3L^{Me} (3.05 mmol, 2 equiv.) was dissolved in anhydrous toluene (20 ml) to which 0.34 g [Sc(OiPr)$_3$] (1.53 mmol, 1 equiv.) was added and allowed to stir at 298 K for 16 hours. The resulting white precipitate was collected on a frit and dried under dynamic vacuum.

Yield: 0.50 g, 37%

Crystals suitable for X-ray diffraction were isolated by addition of anhydrous toluene to a solution of **8** in anhydrous dichloromethane.

^{1}H NMR (400.13 MHz, Chloroform-*d*, 218 K, δ_H, ppm); 10.86 (1H, *s*, *broad*, N*H*), 10.37 (1H, *s*, *broad*, N*H*), 6.86 (6H, *s*, ArH), 6.70 (6H, *s*, Ar*H*), 4.73 (6H, *d*, *J* = 12.9 Hz, NCH*H*), 3.34 (6H, *m*, *J* = 11.6 Hz, NC*H*H), 2.17 (18H, *s*, ArC*H$_3$*), 1.93 (18H, *s*, ArC*H$_3$*). **^{13}C{^{1}H} NMR** (100.62 MHz, Chloroform-*d*, 238 K, δ_H, ppm); 158.19 (*Ar*), 132.41 (*Ar*H), 128.23 (*Ar*), 128.06 (*Ar*H), 124.42 (*Ar*), 116.37 (*Ar*), 56.94 (N*C*H$_2$), 20.36 (*C*H$_3$), 16.65 (*C*H$_3$). **Elemental (CHN) Analysis** Calculated, for $C_{54}H_{63}N_2O_6Sc$; C: 73.5%; H: 7.42%; N: 3.17%. Experimental; C: 72.5%; H: 7.13%; N: 3.05%

Synthesis of [Sm(HL^{Me})(H$_2$L^{Me})], ***9***

In a fritted Schlenk tube, 0.99 g H_3L^{Me} (2.35 mmol, 2 equiv.) was dissolved in anhydrous dichloromethane (20 ml) to which 0.39 g [Sm(OiPr)$_3$] (1.18 mmol, 1 equiv.) was added and allowed to stir at 298 K for 1 hour. The resulting off-white precipitate was collected on a frit and dried under dynamic vacuum.

Yield: 0.66 g, 56%

Crystals suitable for X-ray diffraction were isolated by addition of anhydrous toluene to a solution of **9** in anhydrous dichloromethane.

Paramagnetic sample; ^{1}H NMR (400.13 MHz, Chloroform-*d*, 328 K, δ_H, ppm); 13.11 (2H, *d*, *broad*, N*H*), 7.57 (6H, *s*, *broad*, Ar*H*), 6.63 (12H, *s*, *broad*, NC*H$_2$*), 6.45 (6H, *s*, *broad*, Ar*H*), 2.30 (18H, *s*, *broad*, C*H$_3$*), –0.79 (18H, *s*, *broad*, C*H$_3$*). **^{13}C{^{1}H} NMR** (100.62 MHz, Chloroform-*d*, 328 K, δ_H, ppm); 157.93 (*Ar*), 132.33 (*Ar*H), 129.72 (*Ar*H), 126.09 (*Ar*), 124.88 (*Ar*), 118.96 (*Ar*), 64.71 (N*C*H$_2$), 20.53 (*C*H$_3$), 14.88 (*C*H$_3$). **Elemental (CHN) Analysis** Calculated, for $C_{54}H_{63}N_2O_6Sm$; C: 65.6%; H: 6.63%; N: 2.83%. Experimental; C: 68.3%; H: 6.66%; N: 2.02%.

Synthesis of [Co(III)Cp$_2$]$^+$[Ce(III)(HL^{Me})$_2$]$^-$, ***10***

For catalytic studies, ***10*** *was typically generated in-situ on treatment of* ***3*** *with a fivefold excess of cobaltocene, [Co(II)Cp$_2$]. Ce(III) is also known to be paramagnetic. Accordingly, isolation and full characterisation of* ***10*** *was not undertaken. Nonetheless, for structure confirmation, crystals suitable for X-ray diffraction were prepared by the following method:*

In the glove box, 25 mg [Ce(HL^{Me})], **3**, (0.026 mmol, 1 equiv.), and 10 mg cobaltocene, [Co(II)Cp$_2$], (0.053 mmol, 2 equiv.) were combined and dissolved in chlorobenzene (~1.5 ml). The solution was immediately passed through a 0.2 µm PTFE syringe filter, and allowed to stand in a clean vial for 20 hours, by which time black-brown needles formed on the base of the vial.

The amenability of ***3*** *to reduction was also confirmed via electrochemical methods (cyclic voltammetry).*

S3. Variable-temperature ^{1}H NMR spectroscopic studies of compounds 1 – 7

Compounds **1** – **7** were subject to variable-temperature ^{1}H NMR studies to determine the temperature at which the rate of inversion of ligand chirality for each respective compounds exceeds the NMR timescale sufficiently for coalescence of diastereotopic methylene (NC*H*) resonances to occur. These studies frequently used crude or impure samples, given the need to preserve higher-purity samples for catalytic studies, this being evident in the corresponding spectra.

Studies for all compounds (**1** – **7**) were initially undertaken in chloroform-*d*. However, compounds **1** – **3** did not exhibit coalescence within a temperature range compatible with chloroform-*d* remaining in the liquid phase. Accordingly, higher-temperature studies for these species were undertaken in *protio*-chlorobenzene. Coalescence temperatures for **1** – **3** were consistently ~353 K, and for **5** and **7** were ~273 K and ~263 K, respectively. A coalescence temperature could not be conclusively determined for **6**, which we attribute to the paramagnetic character of that system. Although a coalescence temperature was determined for **4**, that data was also somewhat ambiguous.

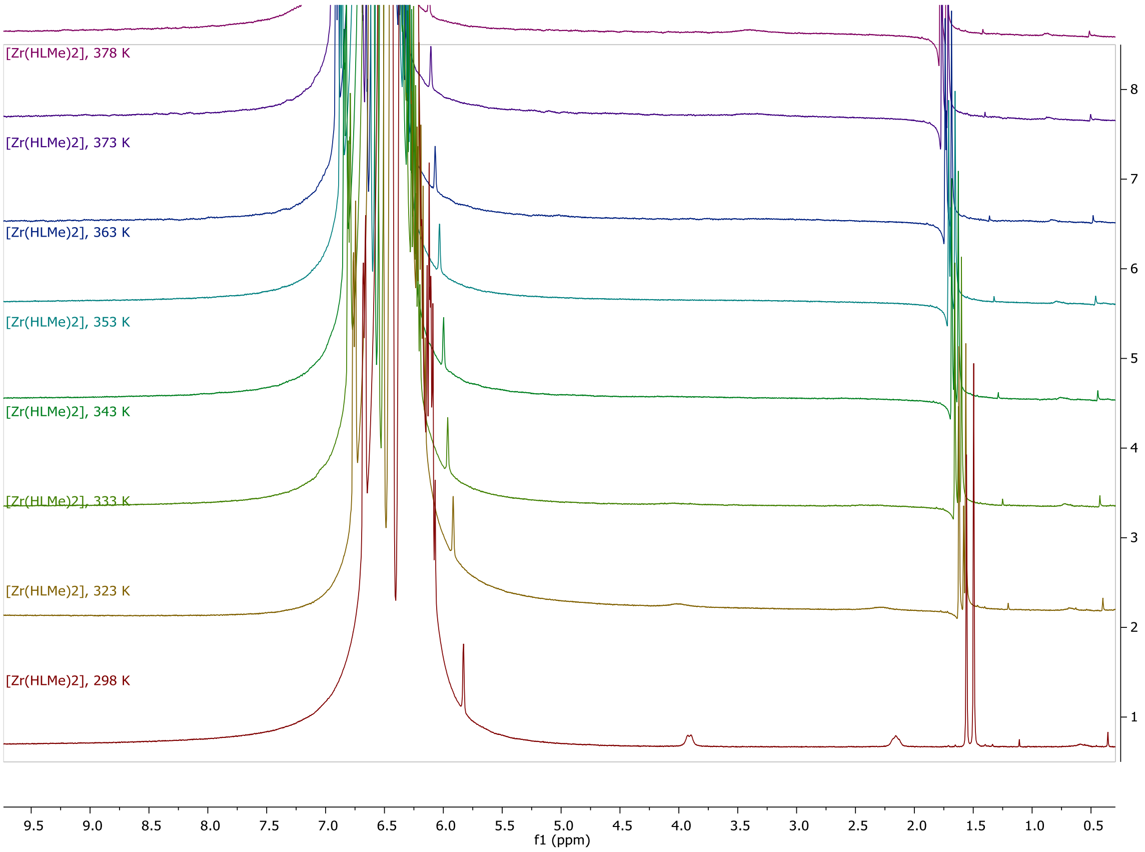

Figure S1. Stacked spectra from variable-temperature ^{1}H NMR studies of **1** in *protio*-chlorobenzene. Visible in the methylene region, two distinct (diastereotopic) resonances between δ = 2 ppm and δ = 4 ppm in the spectrum acquired at 298 K coalesce into a single signal at temperatures exceeding 353 K. Intense signals at δ ~ 6.5 ppm arise from the solvent.

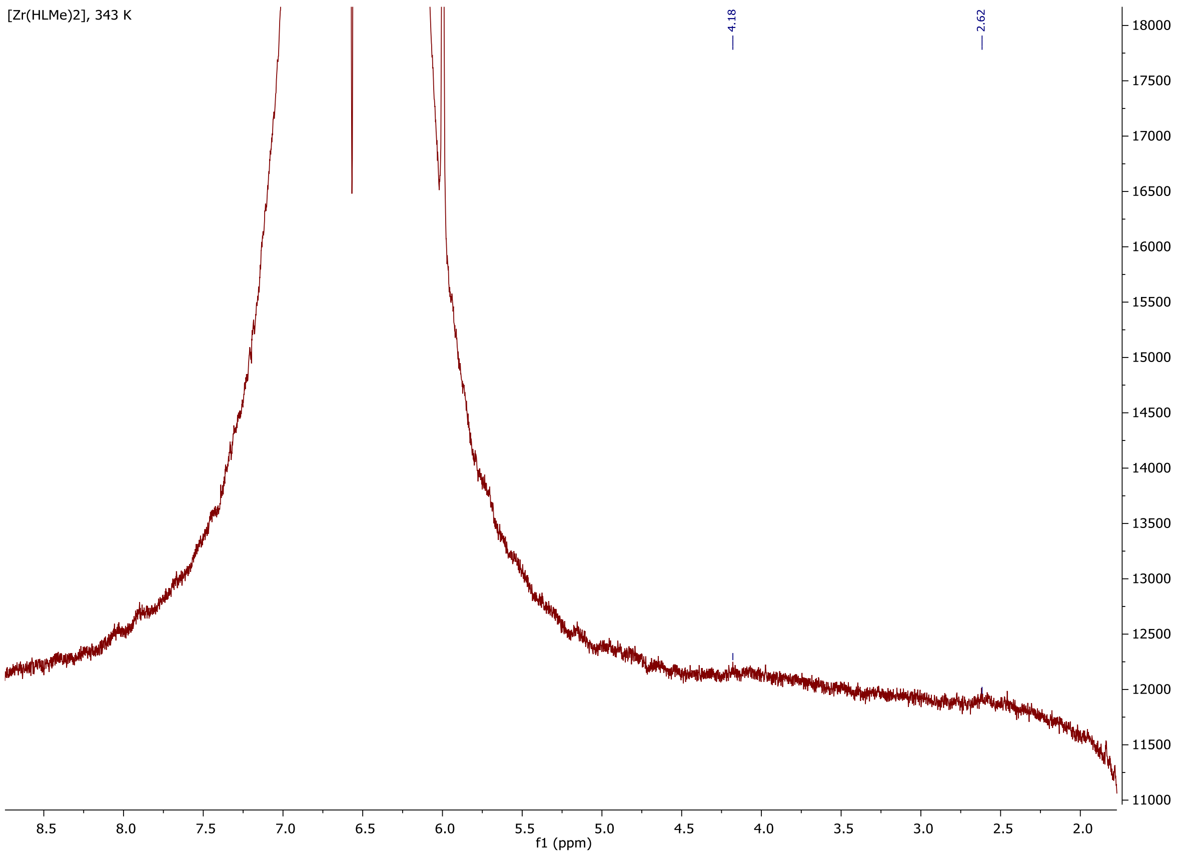

Figure S2. Spectrum acquired at 343 K during variable-temperature ^{1}H NMR studies of **1** in *protio*-chlorobenzene. Visible in the methylene region, two very broad residual diastereotopic signals, showing that the acquisition temperature is approaching the coalescence temperature, T_C.

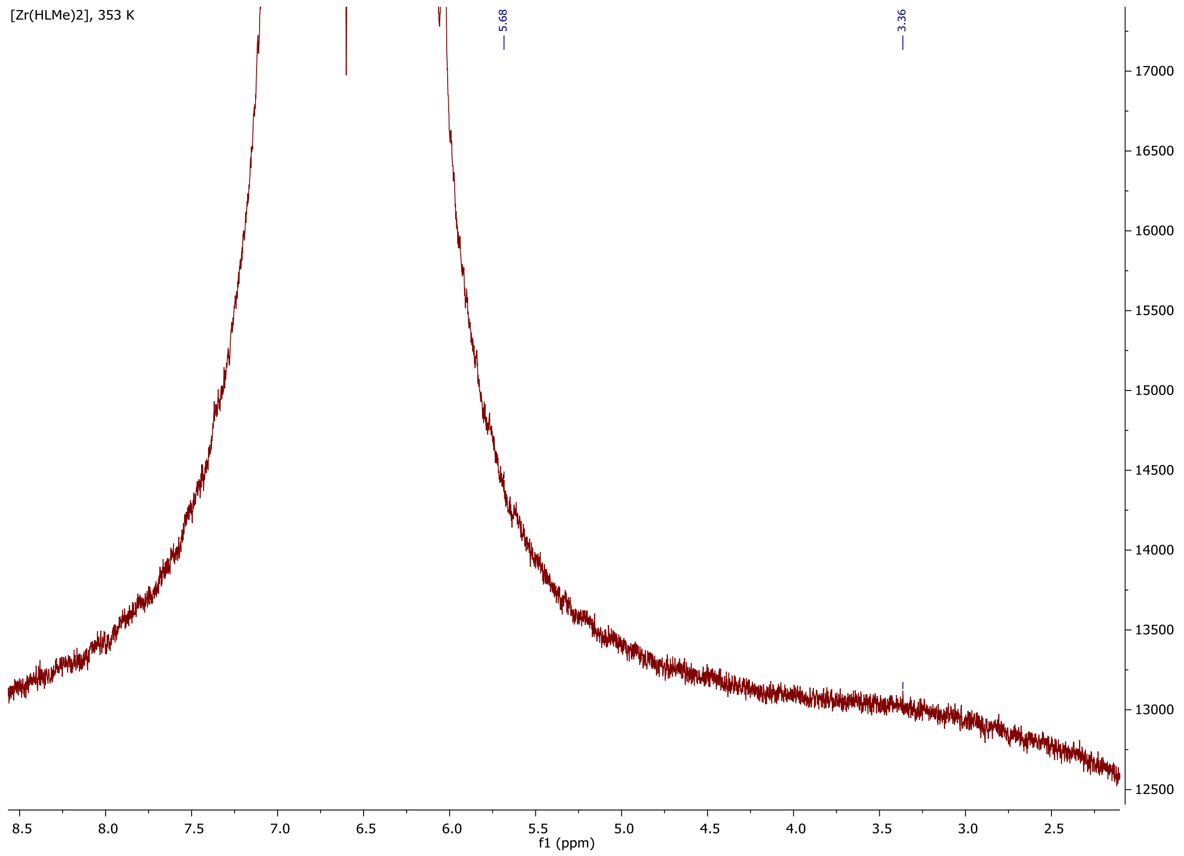

Figure S3. Spectrum acquired at 353 K during variable-temperature ^1H NMR studies of **1** in *protio*-chlorobenzene. Visible in the methylene region, a single very broad signal, almost indiscernible from the baseline, showing that the acquisition temperature is approximately equal to the coalescence temperature, T_C.

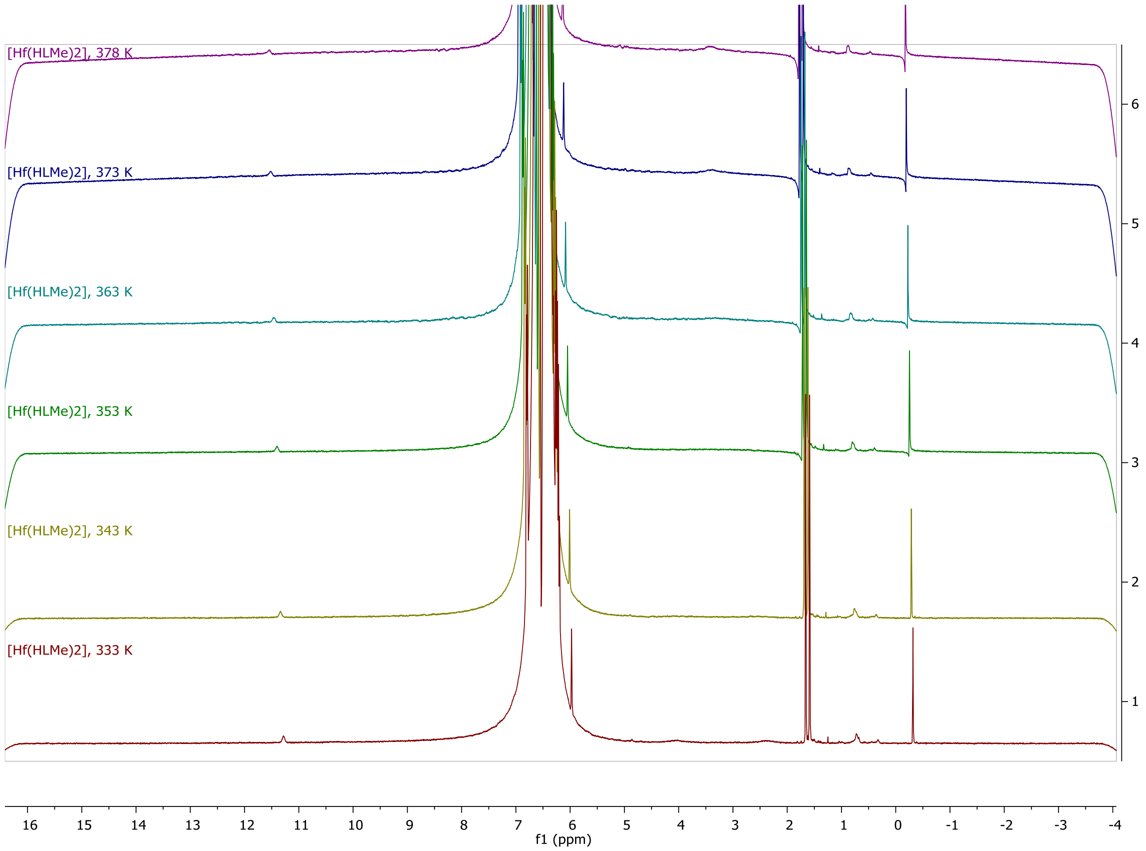

Figure S4. Stacked spectra from variable-temperature ^{1}H NMR studies of **2** in *protio*-chlorobenzene. Visible in the methylene region, two distinct (diastereotopic) resonances between δ = 2 ppm and δ = 4.5 ppm in the spectrum acquired at 333 K coalesce into a single signal at temperatures exceeding 353 K. Intense signals at δ ~ 6.5 ppm arise from the solvent.

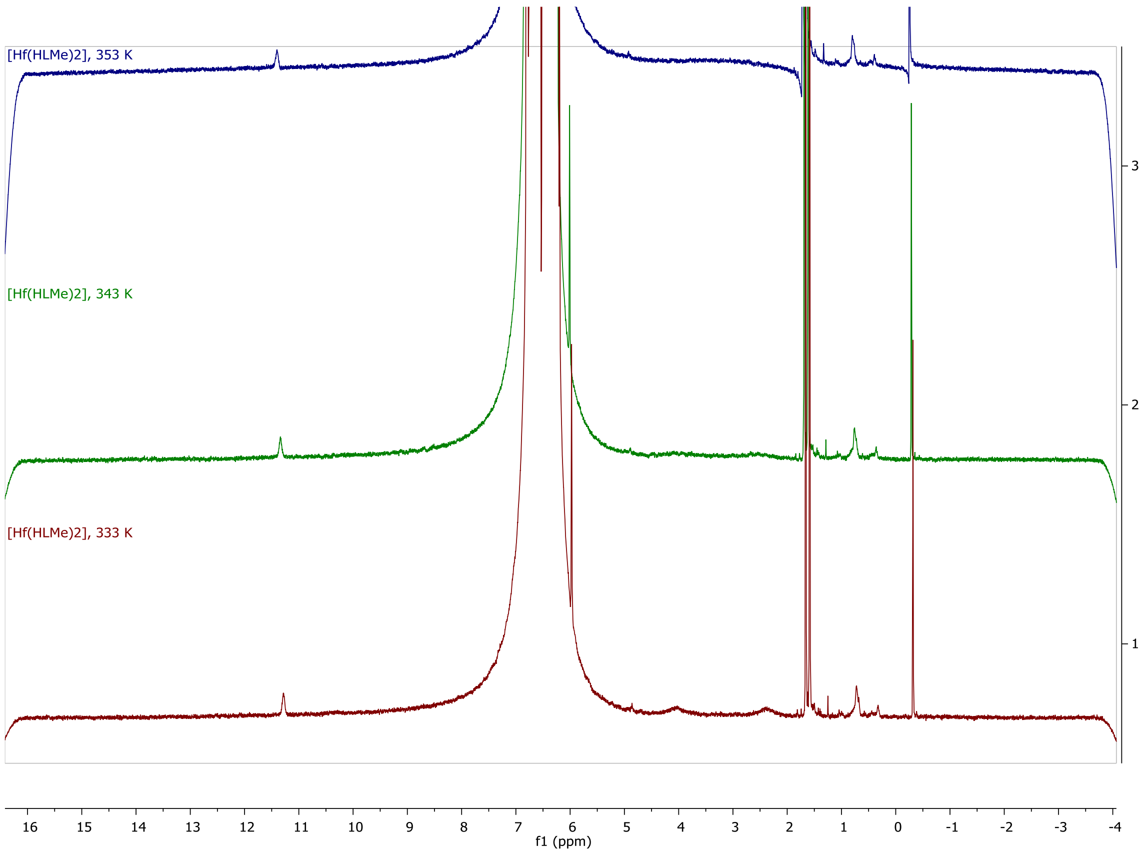

Figure S5. Stacked spectra acquired at 333 K, 343 K and 353 K, respectively, from variable-temperature ^{1}H NMR studies of **2** in *protio*-chlorobenzene. Visible in the methylene region, two distinct (diastereotopic) resonances between δ = 2 ppm and δ = 4.5 ppm in the spectrum acquired at 333 K coalesce into a single broad signal at 353 K. Intense signals at δ ~ 6.5 ppm arise from the solvent.

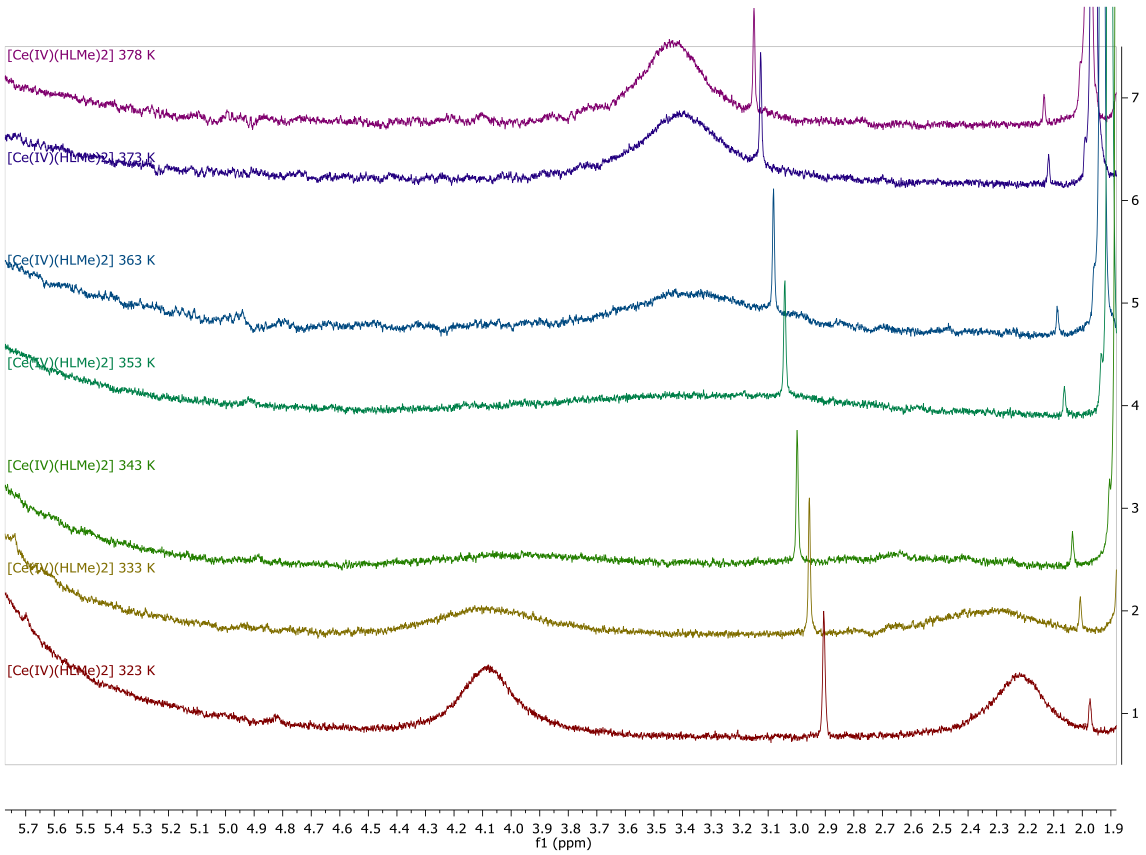

Figure S6. Stacked spectra from variable-temperature ^{1}H NMR studies of **3** in *protio*-chlorobenzene. Visible in the methylene region, two distinct (diastereotopic) resonances between δ = 2 ppm and δ = 4.5 ppm in the spectrum acquired at 333 K coalesce into a single signal at temperatures exceeding 353 K.

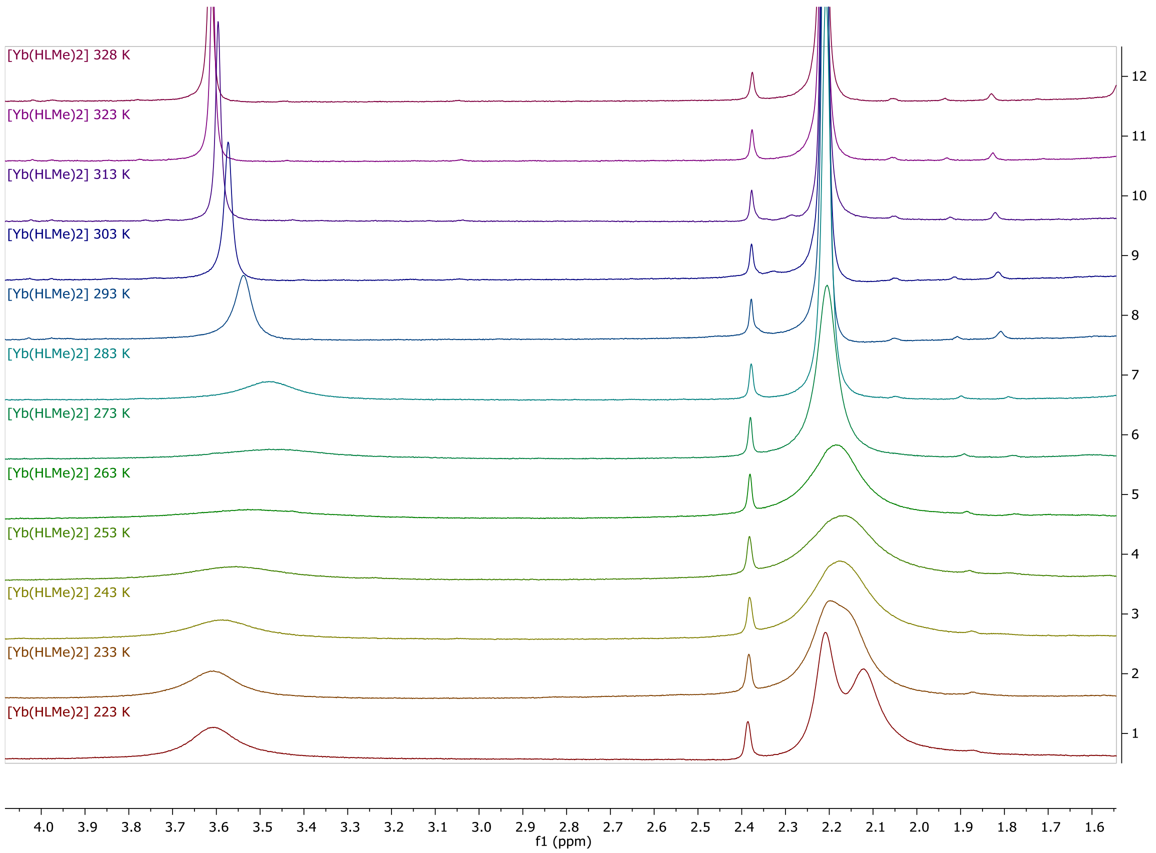

Figure S7. Stacked spectra from variable-temperature ^{1}H NMR studies of paramagnetic compound **4** in chloroform-*d*. Visible in the methylene region, two distinct (diastereotopic) resonances at δ ~ 2.1 ppm and δ ~ 3.6 ppm in the spectrum acquired at 223 K coalesce into a single signal at δ ~ 3.6 ppm at temperatures exceeding 273 K.

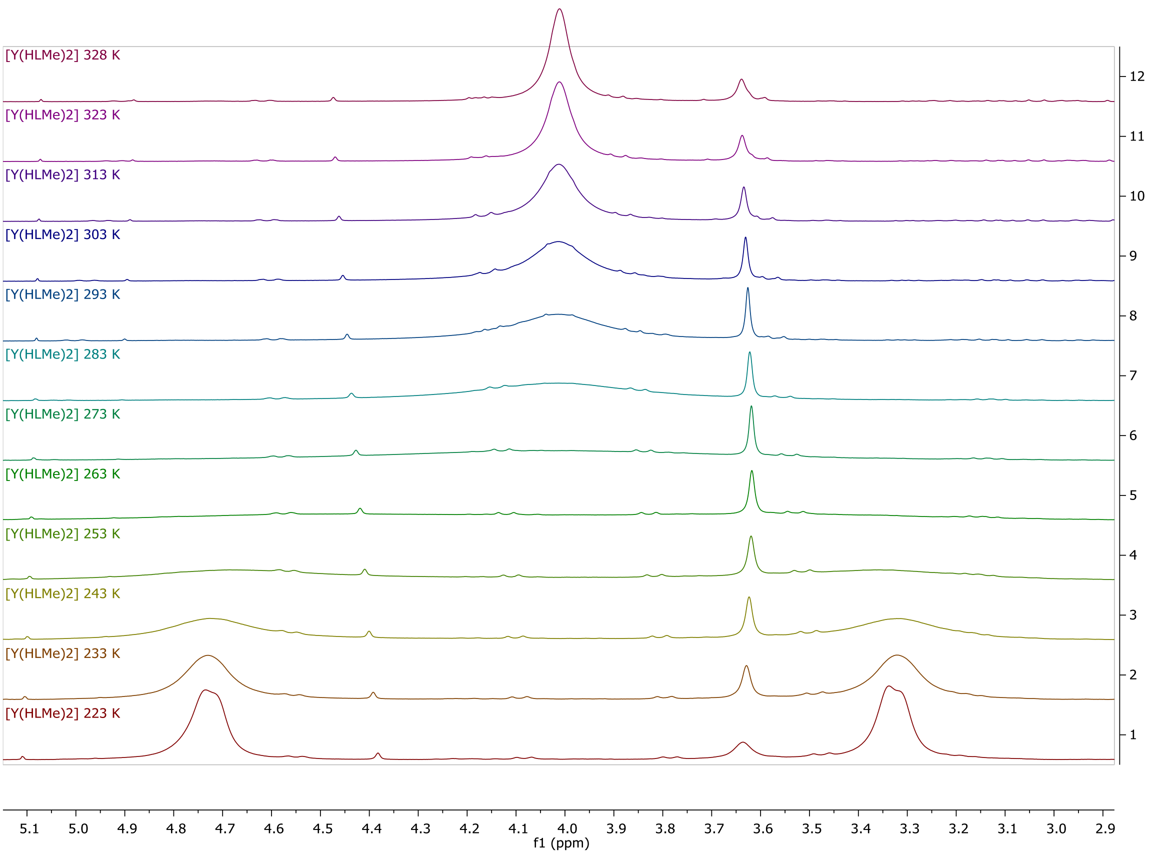

Figure S8. Stacked spectra from variable-temperature ^{1}H NMR studies of **5** in chloroform-*d*. Visible in the methylene region, two distinct (diastereotopic) resonances at δ ~ 3.3 ppm and δ ~ 4.7 ppm in the spectrum acquired at 223 K coalesce into a single signal at δ ~ 3.6 ppm at temperatures exceeding 273 K.

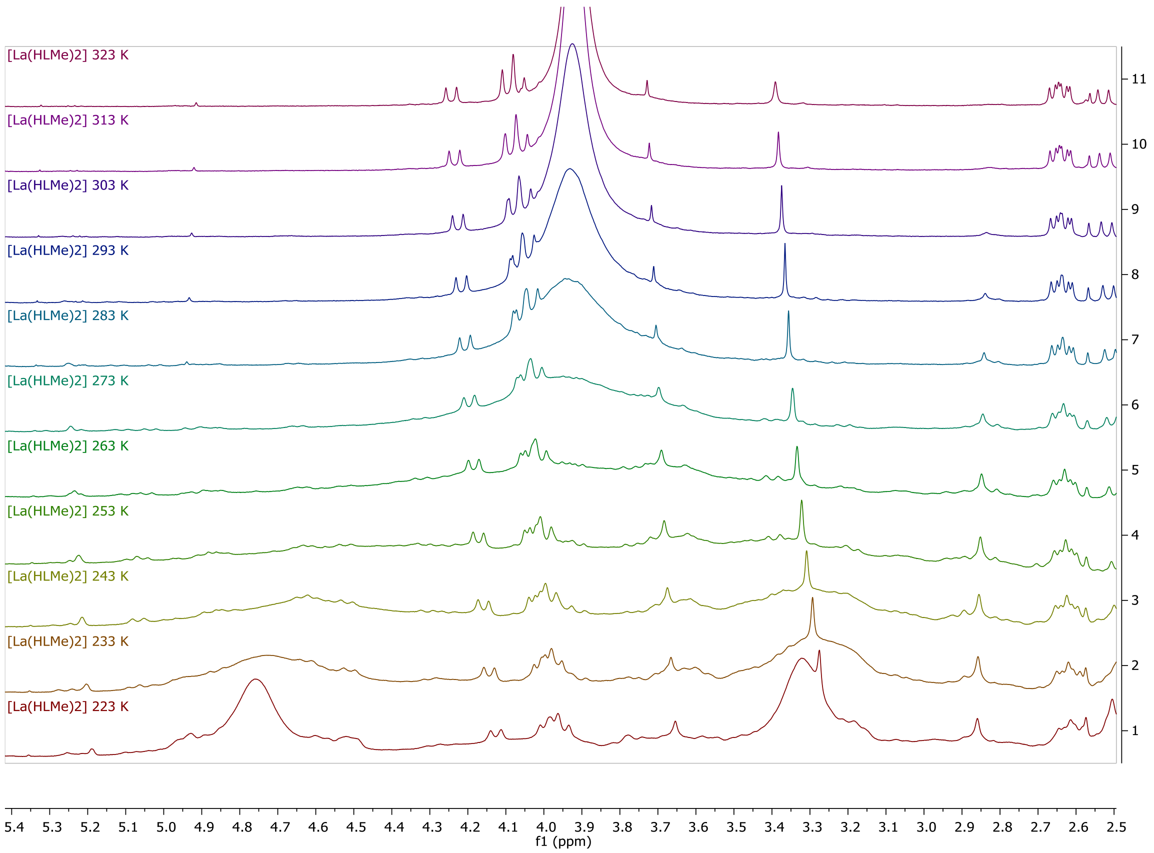

Figure S9. Stacked spectra from variable-temperature ^{1}H NMR studies of **7** in chloroform-*d*. Visible in the methylene region, two distinct (diastereotopic) resonances at δ ~ 3.3 ppm and δ ~ 4.8 ppm in the spectrum acquired at 223 K coalesce into a single signal at δ ~ 3.9 ppm at temperatures exceeding 263 K.

S4. Electrochemical characterisation of Compound 3

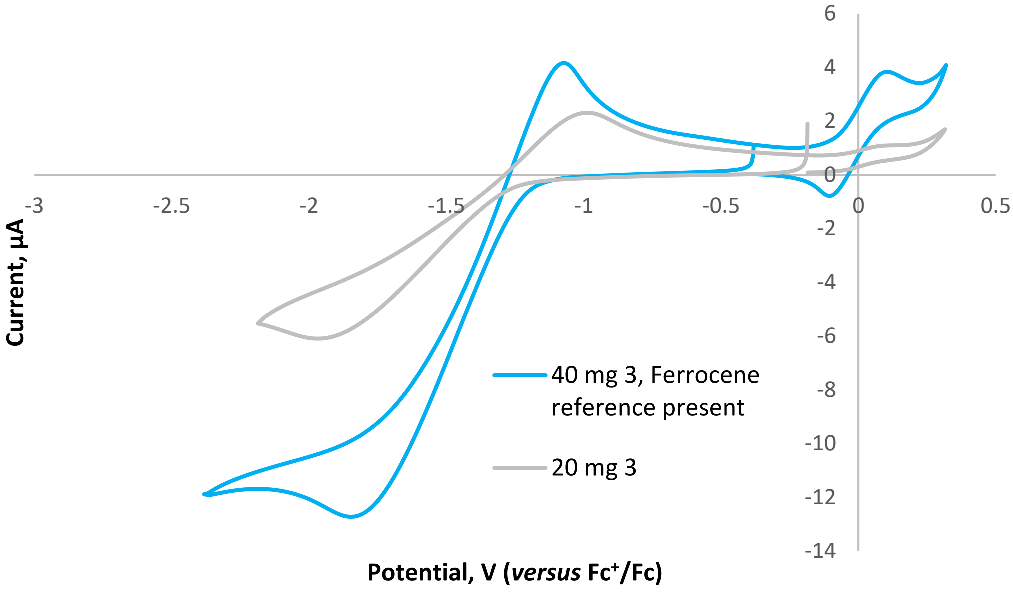

Figure S10. Cyclic voltammograms (CV plots) of a solution of **3** in chlorobenzene in the presence of an electrolyte, showing two concentrations of **3** (with no change to electrolyte concentration), with both CV plots referenced against a ferrocene (Fc^{+}/Fc) internal standard present in the higher-concentration solution of **3**.

Cyclic voltammetry was used to determine the reduction potential of Ce(IV) compound **3**,[9] and thus determine the feasibility of chemical reduction to yield a Ce(III) system. The initial cyclic voltammetry experiment used 20 mg of **3** in approximately 2 ml of chlorobenzene, in the presence of approximately 200 µL of an ionic liquid electrolyte, (trihexyl(tetradecyl)phosphonium tris(pentafluoroethyl)trifluorophosphate), using a scan rate of 10 mV s^{-1}. Clear oxidation and reduction peaks were observed in the resulting cyclic voltammogram (CV). Subsequently, the concentration of **3** was increased twofold, which had the effect of doubling the amplitude (current) of the oxidation and reduction peaks, confirming **3** to be the redox-active species. Finally, a small quantity of ferrocene was added, against which the reduction potential of **3** could be referenced. From the latter dataset, referenced against the ferrocene redox couple (E^0 = 0 V), the reduction potential of **3** under the current conditions was determined to be $E_{1/2}$ = –1.46 V. The CV dataset corresponding to the lower concentration of **3** has, for convenience, been superimposed on the same axes as the plot from which $E_{1/2}$ was determined.

Further cyclic voltammetric studies were undertaken to determine whether the reduction of **3** is a one-electron process. CV data were acquired using several scan rates, ranging from 3.5 mV s^{-1} to 50 mV s^{-1} (Figure S11). The experimental set-up was identical to that described above, except the analyte solution contained a concentration of 19.4 mM **3**, in a solvent phase comprising ~11 vol% (trihexyl(tetradecyl)phosphonium tris(pentafluoroethyl)trifluorophosphate) in anhydrous chlorobenzene. These CV data were referenced against $E_{1/2}$ = –1.46 V, determined in the previous experiment

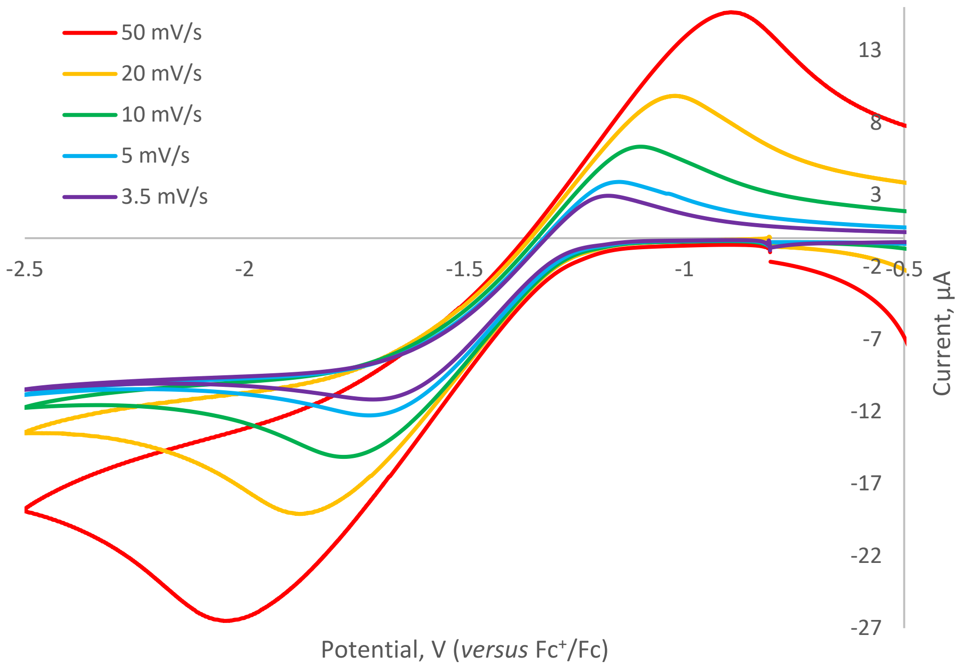

Figure S11. Cyclic voltammograms (CV plots) of a 19.4 mM solution of **3** in chlorobenzene containing 11 vol% ionic liquid electrolyte, at various scan rates. Referenced against $E_{1/2}$, determined *versus* Fc^+/Fc as described above.

Table S1. Data corresponding to variable scan-rate cyclic voltammetry of a 19.4 mM solution of **3** in chlorobenzene containing 11 vol% ionic liquid electrolyte, for application of the Randles-Sevcik equation.

Scan rate, V s^{-1}	sqr(scan rate), V s^{-1}	red peak current, A
0.05	0.2236	-0.0000265
0.02	0.1414	-0.0000191
0.01	0.1000	-0.0000151
0.005	0.0707	-0.0000123
0.0035	0.0592	-0.0000112

Application of the Randles-Sevcik equation to these data (Table S1, Figure S12), on the basis of a one-electron process, determined the diffusion coefficient of **3** under the analytical conditions to be 6.71×10^{-11} m^2 s^{-1}.[9] This value was validated by comparison with a diffusion coefficient calculated using the Stokes-Einstein-Sutherland equation,[10] for which **3** was considered to be a spherical species with a radius of 1.6 nm (from solid phase structure), and the viscosity of the solvent phase was assumed to be equal to that of water. The calculated value was 2.7×10^{-10} m^2 s^{-1}, significantly exceeding that determined electrochemically, and therefore incompatible with a multi-electron process. Closer agreement between experimental and calculated diffusion coefficients may be anticipated if the viscosity of the solvent system were to be more accurately reflected in application of the Stokes-Einstein-Sutherland equation. Nonetheless, the significant peak (potential) separation observed in the CV plots obtained for **3** is consistent with a large, slowly-diffusing species undergoing a (reversible) single-electron process.

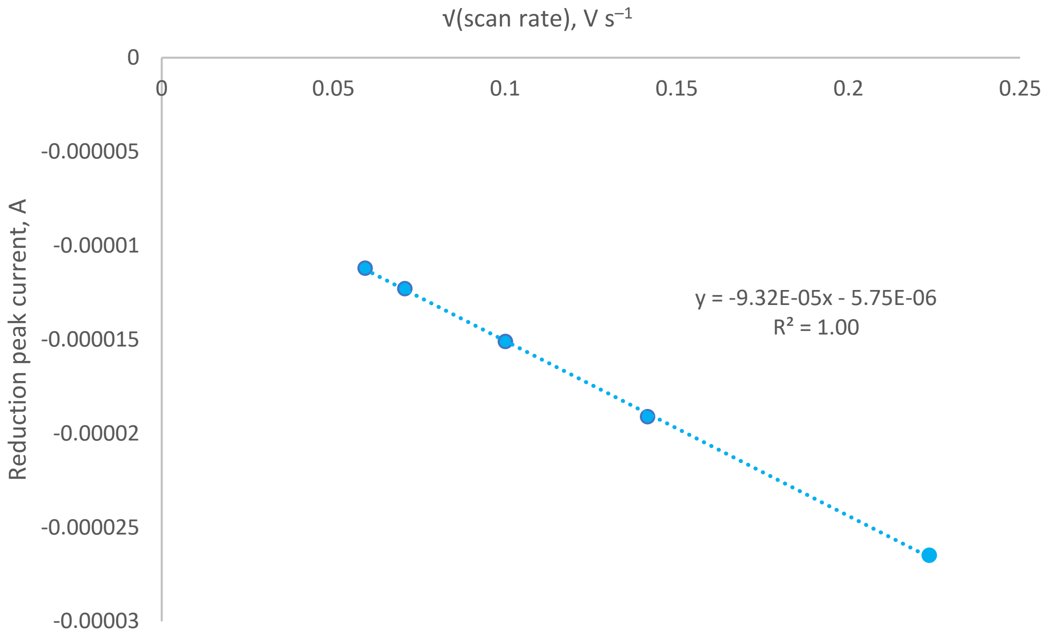

Figure S12. Plot of reduction peak current *versus* the square root of CV scan rate, showing a linear response, for application of the Randles-Sevcik equation.

S5. Polymerisation methods

Polymerisation reactions in the current work were typically carried out under an inert argon atmosphere in 4 ml vials, fitted with PTFE-lined melamine resin screw caps. Generally, reactions were undertaken for the purpose of collecting kinetic data, and were accordingly carried out in parallel, with cessation of heating after various reaction durations. Each vial, unless specified otherwise, was charged, in the glove box, with 500±5 mg of *rac*-lactide, and a magnetic stirrer bar (6 mm). A stock solution (of between 2.5 – 5 ml, dependent upon the number of reactions being undertaken) containing the relevant catalyst and, where appropriate, 4-methylbenzyl alcohol, was prepared in anhydrous chlorobenzene, such that the desired loadings of each species would be dosed on introducing 500 µL of the solution to each vial containing the monomer. After dosing 500 µL of the stock solution, each vial was sealed and removed from the glove box, before being placed into a pre-heated DrySyn aluminium heating block, with the desired temperature being maintained *via* a thermocouple placed into a drilled hole in the block. The monomer consistently and rapidly became fully dissolved upon heating ([LA] = 3.47 mol dm^{-3}). After each reaction vial had been heated for the required duration, it was immediately removed from the heating block and placed in an ice-filled beaker before analysis of the product mixture. Sample preparation for ^{1}H NMR analyses was undertaken by addition of the crude reaction mixture to chloroform-*d* under ambient conditions. Similarly, SEC analysis was carried out by preparing a sample solution containing 4 mg ml^{-1} of the crude reaction mixture in tetrahydrofuran, corresponding to a concentration of lactyl species of 2 mg ml^{-1}.

Reactions carried out under dilute conditions were undertaken in anhydrous chlorobenzene, under an inert argon atmosphere, with magnetic stirring, in sealed J Young ampoules. On completion of the reaction, NMR samples were prepared by removal of the solvent from an aliquot of the product mixture by exposure to dynamic vacuum for 20 hours in a vacuum oven (set to 30℃), before dissolving the residue in chloroform-*d*. Where appropriate, polymer samples were isolated for SEC analysis by

precipitation from excess methanol, before drying under dynamic vacuum and dissolving the product in tetrahydrofuran at a concentration of 2 mg ml^{-1}.

High-temperature, solvent-free reactions were undertaken under an inert argon atmosphere, with magnetic stirring, in sealed J Young ampoules. In a glove box, the ampoule was charged with 10 g of *L*-lactide, and the catalyst, **7**, and co-initiator (4-methylbenzyl alcohol) introduced as a 280 µL aliquot of a solution in anhydrous chlorobenzene, wherein [4-MeBnOH] = 12.5% wt/vol, and [**7**] varied according to the required loading. The ampoule was then partially submerged (to above the level of the lactide within) in a silicone oil bath that had been pre-heated to, and maintained at, 180±3˚C (thermocouple). After the desired reaction duration had elapsed, the ampoule was removed from the oil bath and immediately submerged in cold water (<20˚C). The solidified reaction mixture was then dissolved in dichloromethane and transferred to a round-bottomed flask, before removal of the solvent *in vacuo* (rotary evaporator), and analysis of the products *via* SEC and NMR spectroscopy.

Determination of P_r in the ROP of *rac*-LA was *via* application of Bernoullian statistics to the homonuclear decoupled methine region of the ^{1}H NMR spectrum of the polymer product, as described by Coates and co-workers.[11]

*Note: this method is generally not applicable to any scenario in which epimerisation has occurred. However, analysis of such data pertaining to the ROP of L-LA under conditions anticipated to promote proliferation of side reactions, including epimerisation, in this work revealed that the signal corresponding to the [sis] tetrad, describing a sequence of alternating syndio, iso and syndio ester linkages in the polymer backbone, accounted for 7% and 8% of methine protons in the cases of **6** and **7**, respectively. Given that, for a high-molecular weight polymer sample, the molar quantity of tetrads present is approximately equal to the total number of stereocenters, and that a low concentration of statistically distributed stereochemical defects will result in negligibly few occurring in adjacent positions, each defect therefore giving rise to approximately one [sis] tetrad, this data corresponds to epimerisation having occurred at less than 10% of all stereocenters.*

S6. Polymerisation data tables

Data for all polymerisation reactions undertaken in the course of the current work are detailed in the following tables.

Table S2. Polymerisation data corresponding to application of catalysts **1** – **3** to the ROP of *rac*-LA at 120˚C in solution in chlorobenzene, including *ex-situ* kinetic studies and other polymerisation experiments.

Entry	Cat.	[b][*rac*-LA]:[Cat.]:[4-MeBnOH]	Duration, min	[c]Conversion, %	[d]P_r	[e]M_n^{Theo}, g mol^{-1}	[f]M_n^{SEC}, g mol^{-1}	[f]M_w^{SEC}, g mol^{-1}	[f]$Đ_M$
[a]1	**1**	1000:1:3	120	56	0.81	27026	38150	39800	1.04
[a]2	**1**	1000:1:3	180	80	0.80	38556	56950	66400	1.17
[a]3	**1**	1000:1:3	240	86	0.80	41438	60850	75150	1.24
[a]4	**1**	1000:1:3	360	93	0.79	44801	69350	103950	1.50
[a]5	**1**	1000:1:3	480	96	0.79	46242	71900	120600	1.68
[a]6	**1**	1000:1:3	960	96	0.77	46242	66200	113000	1.71
[a]7	**1**	1000:1:10	960	96	0.77	13958	23500	39550	1.68
[a]8	**2**	1000:1:3	120	40	0.80	19339	25250	26200	1.04
[a]9	**2**	1000:1:3	180	58	0.80	27986	38850	40750	1.05
[a]10	**2**	1000:1:3	240	79	0.80	38075	49600	57050	1.15
[a]11	**2**	1000:1:3	360	89	0.79	42879	59800	82550	1.38
[a]12	**2**	1000:1:3	480	95	0.80	45762	61950	107950	1.74
[a]13	**2**	1000:1:3	960	95	0.80	45762	61650	102500	1.66
[a]14	**2**	1000:1:10	960	96	0.76	13958	23200	39750	1.71
[a]15	**3**	1000:1:3	60	15	0.65	7328	7150	16450	2.30
[a]16	**3**	1000:1:3	120	42	0.62	20300	23150	37650	1.63
[a]17	**3**	1000:1:3	180	62	0.60	29908	38250	61900	1.62
[a]18	**3**	1000:1:3	240	75	0.60	36154	46800	76300	1.63
[a]19	**3**	1000:1:3	360	87	0.58	41919	53550	84750	1.58
[a]20	**3**	1000:1:3	480	90	0.57	43360	56350	91350	1.62
[a]21	**3**	1000:1:3	960	95	0.55	45762	60450	98350	1.63
[a]22	**3**	1000:1:10	240	82	0.57	11952	18050	28700	1.59
[a]23	**3**	1000:1:10	960	95	0.57	13827	22750	37500	1.65

[a] Conditions: 500±5 mg *rac*-LA in 500 µL anhydrous PhCl dosed as stock solution of Cat. + 4-MeBnOH, 120˚C (aluminium heating block with thermocouple), magnetic stirring. [b] Molar ratio. [c] Conversion determined *via* ^{1}H NMR spectroscopy, by integration of LA and PLA methine resonances. [d] P_r calculated *via* polymer microstructure analysis (^{1}H{^{1}H} NMR); $P_r = \sqrt{(2-[sis])}$.[11] [e] M_n^{Theo} calculated from conversion and alcohol concentration: $M_n^{Theo} = \left\{\left(M_{r,LA} \times \frac{\%_{conv}}{100} \times \frac{[LA]}{[4-MeBnOH]}\right) + M_{r,4-MeBnOH}\right\}$. [f] Determined *via* size-exclusion chromatography (SEC) in THF, using a refractive index detector calibrated against polystyrene standards of known molecular weight.

Table S3. Polymerisation data corresponding to application of **4** to the ROP of *rac*-LA at 120˚C in solution in chlorobenzene, including *ex-situ* kinetic studies, both in the presence and absence, respectively, of exogenous 4-methybenzyl alcohol, and other polymerisation experiments.

Entry	Cat.	[b][*rac*-LA]:[Cat.]: [4-MeBnOH]	Duration, min	[c]Conversion, %	[d]P_r	[e]M_n^{Theo}, g mol^{-1}	[f]M_n^{SEC}, g mol^{-1}	[f]M_w^{SEC}, g mol^{-1}	[f]$Đ_M$
[a]1	**4**	1000:1:0	2	0	N/A	N/A	N/A	N/A	N/A
[a]2	**4**	1000:1:0	5	0	N/A	N/A	N/A	N/A	N/A
[a]3	**4**	1000:1:0	10	0	N/A	N/A	N/A	N/A	N/A
[a]4	**4**	1000:1:0	15	0	N/A	N/A	N/A	N/A	N/A
[a]5	**4**	1000:1:0	30	9	0.62	N/A	12800	15400	1.20
[a]6	**4**	1000:1:0	60	16	0.62	NA	18450	24950	1.35
[a]7	**4**	1000:1:0	120	43	0.60	N/A	37850	62600	1.65
[a]8	**4**	1000:1:0	180	75	0.57	N/A	55750	86800	1.56
[a]9	**4**	1000:1:0	240	90	0.57	N/A	59550	102150	1.72
[a]10	**4**	1000:1:3	2	0	N/A	122	N/A	N/A	N/A
[a]11	**4**	1000:1:3	5	0	N/A	122	N/A	N/A	N/A
[a]12	**4**	1000:1:3	10	6	0.57	3005	1800	1950	1.09
[a]13	**4**	1000:1:3	15	7	0.57	3485	3000	3250	1.09
[a]14	**4**	1000:1:3	30	14	0.60	6848	5650	6250	1.10
[a]15	**4**	1000:1:3	60	34	0.60	16456	14700	18700	1.27
[a]16	**4**	1000:1:3	120	81	0.57	39036	32550	53050	1.63
[a]17	**4**	1000:1:3	180	94	0.55	45281	36500	65100	1.78
[a]18	**4**	1000:1:3	240	96	0.55	46242	38400	69000	1.80
[a]19	**4**	500:1:0	60	32	0.60	N/A	21000	32200	1.53
[a]20	**4**	500:1:0	240	96	0.55	N/A	49150	83000	1.69
[a]21	**4**	250:1:0	60	47	0.58	N/A	17700	27550	1.56
[a]22	**4**	250:1:0	240	96	0.55	N/A	33100	65700	1.99
[a]23	**4**	1000:1:10	60	71	0.58	10355	14050	18600	1.33
[a]24	**4**	1000:1:10	240	96	0.53	13958	20200	35900	1.78

[a] Conditions: 500±5 mg *rac*-LA in 500 µL anhydrous PhCl dosed as stock solution of Cat. + 4-MeBnOH where relevant, 120˚C (aluminium heating block with thermocouple), magnetic stirring. [b] Molar ratio. [c] Conversion determined *via* ^{1}H NMR spectroscopy, by integration of LA and PLA methine resonances. [d] P_r calculated *via* polymer microstructure analysis (^{1}H{^{1}H} NMR); P_r = √(2-[*sis*]).[11] [e] M_n^{Theo} assuming an immortal kinetic regime with a catalyst bearing no functional initiating group, calculated from conversion and alcohol concentration: $M_n^{Theo} = \left\{\left(M_{r,LA} \times \frac{\%_{conv}}{100} \times \frac{[LA]}{[4-MeBnOH]}\right) + M_{r,4-MeBnOH}\right\}$. [f] Determined *via* size-exclusion chromatography (SEC) in THF, using a refractive index detector calibrated against polystyrene standards of known molecular weight.

Table S4. Polymerisation data corresponding to application of **5** to the ROP of *rac*-LA at 120˚C in solution in chlorobenzene, including *ex-situ* kinetic studies, both in the presence and absence, respectively, of exogenous 4-methybenzyl alcohol, and other polymerisation experiments.

Entry	Cat.	^{b}[*rac*-LA]:[Cat.]: [4-MeBnOH]	Duration, min	^{c}Conversion, %	$^{d}P_r$	$^{e}M_n^{Theo}$, g mol^{-1}	$^{f}M_n^{SEC}$, g mol^{-1}	$^{f}M_w^{SEC}$, g mol^{-1}	$^{f}Đ_M$
^{a}1	**5**	1000:1:0	2	0	N/A	N/A	N/A	N/A	N/A
^{a}2	**5**	1000:1:0	5	2	N/A	N/A	6100	6900	1.14
^{a}3	**5**	1000:1:0	10	7	0.58	N/A	9800	11450	1.17
^{a}4	**5**	1000:1:0	15	12	0.63	N/A	17650	22850	1.29
^{a}5	**5**	1000:1:0	30	27	0.62	N/A	28350	43800	1.55
^{a}6	**5**	1000:1:0	60	63	0.58	N/A	49000	81050	1.65
^{a}7	**5**	1000:1:0	90	90	0.57	N/A	63200	98650	1.56
^{a}8	**5**	1000:1:0	120	95	0.57	N/A	62700	103850	1.66
^{a}9	**5**	1000:1:3	2	5	N/A	2524	1850	2000	1.08
^{a}10	**5**	1000:1:3	5	8	0.55	3965	4250	4600	1.08
^{a}11	**5**	1000:1:3	10	15	0.58	7328	8850	10350	1.17
^{a}12	**5**	1000:1:3	15	25	0.60	12133	13600	16400	1.20
^{a}13	**5**	1000:1:3	30	53	0.60	25584	24950	37100	1.49
^{a}14	**5**	1000:1:3	60	87	0.55	41919	41900	69450	1.66
^{a}15	**5**	1000:1:3	90	96	0.55	46242	44750	77850	1.74
^{a}16	**5**	1000:1:3	120	97	0.57	46723	47150	77900	1.65
^{a}17	**5**	500:1:0	60	86	0.57	N/A	49050	79650	1.62
^{a}18	**5**	500:1:0	120	97	0.55	N/A	51350	84700	1.65
^{a}19	**5**	250:1:0	60	96	0.57	N/A	37350	63550	1.70
^{a}20	**5**	250:1:0	120	97	0.53	N/A	37700	61300	1.63
^{a}21	**5**	1000:1:10	60	97	0.53	14102	22150	35450	1.60

^{a} Conditions: 500±5 mg *rac*-LA in 500 µL anhydrous PhCl dosed as stock solution of Cat. + 4-MeBnOH where relevant, 120˚C (aluminium heating block with thermocouple), magnetic stirring. ^{b} Molar ratio. ^{c} Conversion determined *via* ^{1}H NMR spectroscopy, by integration of LA and PLA methine resonances. ^{d} P_r calculated *via* polymer microstructure analysis (^{1}H{^{1}H} NMR); P_r = √(2-[*sis*]).[11] ^{e} M_n^{Theo} assuming an immortal kinetic regime with a catalyst bearing no functional initiating group, calculated from conversion and alcohol concentration: $M_n{}^{Theo} = \left\{\left(M_{r,LA} \times \frac{\%_{conv}}{100} \times \frac{[LA]}{[4-MeBnOH]}\right) + M_{r,4-MeBnOH}\right\}$. ^{f} Determined *via* size-exclusion chromatography (SEC) in THF, using a refractive index detector calibrated against polystyrene standards of known molecular weight.

Table S5. Polymerisation data corresponding to application of **6** to the ROP, variously, of *rac*-LA and *L*-LA at 120° C in solution in chlorobenzene, including *ex-situ* kinetic studies, both in the presence and absence, respectively, of exogenous 4-methybenzyl alcohol, and other polymerisation experiments.

Entry	Cat.	LA	[b][*rac*-LA]:[Cat.]:[4-MeBnOH]	Duration, min	[c]Conversion, %	[d]P_r	[e]M_n^{Theo}, g mol^{-1}	[f]M_n^{SEC}, g mol^{-1}	[f]M_w^{SEC}, g mol^{-1}	[f]$Đ_M$
[a]1	**6**	*rac*	1000:1:0	2	0	N/A	N/A	N/A	N/A	N/A
[a]2	**6**	*rac*	1000:1:0	5	13	N/A	N/A	13100	23100	1.76
[a]3	**6**	*rac*	1000:1:0	10	32	0.63	N/A	45050	66100	1.47
[a]4	**6**	*rac*	1000:1:0	15	54	0.58	N/A	63950	98200	1.54
[a]5	**6**	*rac*	1000:1:0	30	85	0.53	N/A	85500	134300	1.57
[a]6	**6**	*rac*	1000:1:0	60	95	0.53	N/A	73200	132500	1.81
[a]7	**6**	*rac*	1000:1:3	2	11	0.60	5407	6950	7850	1.13
[a]8	**6**	*rac*	1000:1:3	5	34	0.58	16456	12900	22950	1.78
[a]9	**6**	*rac*	1000:1:3	10	60	0.60	28947	24800	47400	1.91
[a]10	**6**	*rac*	1000:1:3	15	86	0.57	41438	25200	57050	2.26
[a]11	**6**	*rac*	1000:1:3	30	95	0.51	45762	53250	83950	1.58
[a]12	**6**	*rac*	1000:1:3	60	97	0.51	46723	50400	80650	1.60
[a]13	**6**	*rac*	500:1:0	60	96	0.51	N/A	69400	108600	1.56
[a]14	**6**	*rac*	250:1:0	60	97	0.55	N/A	45900	73000	1.59
[a]15	**6**	*rac*	1000:1:10	60	97	0.51	14102	21650	35300	1.63
[a]16	**6**	*L*	250:1:0	120	96	N/A	N/A	33850	53050	1.57

[a] Conditions: 500±5 mg LA in 500 µL anhydrous PhCl dosed as stock solution of Cat. + 4-MeBnOH where relevant, 120°C (aluminium heating block with thermocouple), magnetic stirring. [b] Molar ratio. [c] Conversion determined *via* ^{1}H NMR spectroscopy, by integration of LA and PLA methine resonances. [d] P_r calculated *via* polymer microstructure analysis (^{1}H{^{1}H} NMR); $P_r = \sqrt{(2-[sis])}$.[11] [e] M_n^{Theo} assuming an immortal kinetic regime with a catalyst bearing no functional initiating group, calculated from conversion and alcohol concentration: $M_n^{Theo} = \left\{\left(M_{r,LA} \times \frac{\%_{conv}}{100} \times \frac{[LA]}{[4-MeBnOH]}\right) + M_{r,4-MeBnOH}\right\}$. [f] Determined *via* size-exclusion chromatography (SEC) in THF, using a refractive index detector calibrated against polystyrene standards of known molecular weight.

Table S6. Polymerisation data corresponding to application of **7** to the ROP, variously, of *rac*-LA and *L*-LA at 120° C in solution in chlorobenzene, including *ex-situ* kinetic studies, both in the presence and absence, respectively, of exogenous 4-methybenzyl alcohol, and other polymerisation experiments.

**Entry**	**Cat.**	**LA**	**[b][*rac*-LA]:[Cat.]: [4-MeBnOH]**	**Duration, min**	**[c]Conversion, %**	**[d]P_r**	**[e]M_n^{Theo}, g mol^{-1}**	**[f]M_n^{SEC}, g mol^{-1}**	**[f]M_w^{SEC}, g mol^{-1}**	**[f]$Đ_M$**
[a]1	**7**	*rac*	1000:1:0	2	0	N/A	N/A	N/A	N/A	N/A
[a]2	**7**	*rac*	1000:1:0	5	17	0.51	N/A	25350	37050	1.46
[a]3	**7**	*rac*	1000:1:0	10	48	0.55	N/A	24950	60250	2.42
[a]4	**7**	*rac*	1000:1:0	15	61	0.55	N/A	25250	75200	2.98
[a]5	**7**	*rac*	1000:1:0	30	84	0.57	N/A	81850	124350	1.52
[a]6	**7**	*rac*	1000:1:0	60	92	0.55	N/A	85800	135300	1.58
[a]7	**7**	*rac*	1000:1:3	2	15	0.49	7328	8500	10400	1.22
[a]8	**7**	*rac*	1000:1:3	5	45	0.57	21741	26900	39500	1.47
[a]9	**7**	*rac*	1000:1:3	10	78	0.53	37595	42650	67700	1.59
[a]10	**7**	*rac*	1000:1:3	15	88	0.51	42399	45900	73250	1.60
[a]11	**7**	*rac*	1000:1:3	30	94	0.55	45281	51450	81650	1.59
[a]12	**7**	*rac*	1000:1:3	60	96	0.49	46242	26800	64800	2.42
[a]13	**7**	*rac*	500:1:0	60	96	0.53	N/A	68700	108600	1.58
[a]14	**7**	*rac*	250:1:0	60	97	0.49	N/A	44300	70750	1.60
[a]15	**7**	*rac*	1000:1:10	60	97	0.49	14102	19150	32900	1.72
[a]16	**7**	*L*	250:1:0	120	96	N/A	N/A	36900	58100	1.58

[a] Conditions: 500±5 mg LA in 500 µL anhydrous PhCl dosed as stock solution of Cat. + 4-MeBnOH where relevant, 120°C (aluminium heating block with thermocouple), magnetic stirring. [b] Molar ratio. [c] Conversion determined *via* ^1H NMR spectroscopy, by integration of LA and PLA methine resonances. [d] P_r calculated *via* polymer microstructure analysis ($^1H\{^1H\}$ NMR); $P_r = \sqrt{(2\text{-}[sis])}$.[11] [e] M_n^{Theo} assuming an immortal kinetic regime with a catalyst bearing no functional initiating group, calculated from conversion and alcohol concentration: $M_n^{Theo} = \left\{\left(M_{r,LA} \times \frac{\%_{conv}}{100} \times \frac{[LA]}{[4-MeBnOH]}\right) + M_{r,4-MeBnOH}\right\}$. [f] Determined *via* size-exclusion chromatography (SEC) in THF, using a refractive index detector calibrated against polystyrene standards of known molecular weight.

Table S7. Polymerisation data corresponding to application of **10** (generated *in-situ via* treatment of **3** with excess cobaltocene) to the ROP, variously, of *rac*-LA and *L*-LA at 120˚C in solution in chlorobenzene, including *ex-situ* kinetic studies and other polymerisation experiments.

Entry	Cat.	LA	[b][*rac*-LA]:[Cat.]:[4-MeBnOH]	Duration, min	[c]Conversion, %	[d]P_r	[e]M_n^{SEC}, g mol^{-1}	[e]M_w^{SEC}, g mol^{-1}	[e]M_p^{SEC}, g mol^{-1}	[e]$Đ_M$
[a]1	**10**	*rac*	1000:1:3	2	61	0.62	21250	31400	34650	1.48
[a]2	**10**	*rac*	1000:1:3	5	64	0.58	19250	30300	33150	1.57
[a]3	**10**	*rac*	1000:1:3	10	62	0.62	17350	27000	30350	1.56
[a]4	**10**	*rac*	1000:1:3	12.5	62	0.60	17650	28350	31250	1.61
[a]5	**10**	*rac*	1000:1:3	15	60	0.62	15300	23850	27350	1.56
[a]6	**10**	*rac*	1000:1:3	20	60	0.62	14750	23550	27350	1.60
[a]7	**10**	*rac*	1000:1:3	30	68	0.62	15900	27300	32200	1.72
[a]8	**10**	*rac*	1000:1:3	45	65	0.60	14400	22750	26950	1.58
[a]9	**10**	*rac*	1000:1:3	60	72	0.62	19450	30000	34150	1.54
[a]10	**10**	*L*	1000:2:3	30	87	0.00	12000	29600	42500	2.47

[a] Conditions: 400±5 mg LA in 400 µL anhydrous PhCl dosed as stock solution of Cat. + 4-MeBnOH, 120˚C (aluminium heating block with thermocouple), magnetic stirring. **10** generated *in-situ via* treatment of **3** with a fivefold excess of cobaltocene. [b] Molar ratio. [c] Conversion determined *via* ^1H NMR spectroscopy, by integration of LA and PLA methine resonances. [d] P_r calculated *via* polymer microstructure analysis ($^1H\{^1H\}$ NMR); $P_r = \sqrt{(2-[sis])}$.[11] [e] M_n^{Theo} calculated from conversion and alcohol concentration: $M_n^{Theo} = \left\{\left(M_{r,LA} \times \frac{\%_{conv}}{100} \times \frac{[LA]}{[4-MeBnOH]}\right) + M_{r,4-MeBnOH}\right\}$. [f] Determined *via* size-exclusion chromatography (SEC) in THF, using a refractive index detector calibrated against polystyrene standards of known molecular weight.

Table S8. Polymerisation data corresponding to application, variously, of **7** and **10** (**10** generated *in-situ via* treatment of **3** with excess cobaltocene) to the ROP of *rac*-LA at 80°C in solution in chlorobenzene, including *ex-situ* kinetic studies.

Entry	Cat.	[b][*rac*-LA]:[Cat.]:[4-MeBnOH]	Duration, min	[c]Conversion, %	[d]P_r	[e]M_n^{Theo}, g mol^{-1}	[f]M_n^{SEC}, g mol^{-1}	[f]M_w^{SEC}, g mol^{-1}	[f]M_P^{SEC}, g mol^{-1}	[f]$Đ_M$
[a]1	**7**	1000:2:3	5	10	0.53	4926	N/A	N/A	N/A	N/A
[a]2	**7**	1000:2:3	10	17	0.51	8289	9200	13000	11150	1.41
[a]3	**7**	1000:2:3	20	28	0.53	13574	15350	23500	23050	1.53
[a]4	**7**	1000:2:3	30	42	0.51	20300	22800	34150	32250	1.50
[a]5	**7**	1000:2:3	45	63	0.51	30388	34900	53350	55150	1.53
[a]6	**7**	1000:2:3	90	80	0.47	38556	40300	66250	67600	1.64
[a]7	**7**	1000:2:3	120	87	0.45	41919	44350	71950	74850	1.62
[a]8	**7**	1000:2:3	185	95	0.47	45762	35850	71600	78200	2.00
[a]9	**7**	1000:2:3	240	98	0.49	47203	47350	78100	77050	1.65
[a]10	**10**	1000:2:3	2	28	0.57	13574	13300	14400	13500	1.08
[a]11	**10**	1000:2:3	3	32	0.62	15495	18200	19850	18550	1.09
[a]12	**10**	1000:2:3	7.5	56	0.60	27026	26350	32000	29950	1.21
[a]13	**10**	1000:2:3	10	65	0.60	31349	24000	32650	33200	1.36
[a]14	**10**	1000:2:3	15	77	0.60	37114	31550	43100	40100	1.37
[a]15	**10**	1000:2:3	20	86	0.62	41438	31500	45100	43100	1.43
[a]16	**10**	1000:2:3	30	91	0.60	43840	33700	50700	47700	1.50
[a]17	**10**	1000:2:3	45	94	0.62	45281	32150	50000	47000	1.55
[a]18	**10**	1000:2:3	60	95	0.62	45762	30450	48950	47000	1.61

[a] Conditions: 400±5 mg *rac*-LA in 400 µL anhydrous PhCl dosed as stock solution of Cat. + 4-MeBnOH, 80°C (aluminium heating block with thermocouple), magnetic stirring. **10** generated *in-situ via* treatment of **3** with a fivefold excess of cobaltocene. [b] Molar ratio. [c] Conversion determined *via* ^1H NMR spectroscopy, by integration of LA and PLA methine resonances. [d] P_r calculated *via* polymer microstructure analysis ($^1H\{^1H\}$ NMR); P_r = √(2-[*sis*]).[11] [e] M_n^{Theo} assuming an immortal kinetic regime with a catalyst bearing no functional initiating group, calculated from conversion and alcohol concentration: $M_n{}^{Theo} = \left\{\left(M_{r,LA} \times \frac{\%_{conv}}{100} \times \frac{[LA]}{[4-MeBnOH]}\right) + M_{r,4-MeBnOH}\right\}$. [f] Determined *via* size-exclusion chromatography (SEC) in THF, using a refractive index detector calibrated against polystyrene standards of known molecular weight.

Table S9. Polymerisation data corresponding to control reactions, wherein cobaltocene and cobaltocenium hexafluorophosphate, respectively, were applied to the ROP of *rac*-LA for the purposes of confirming the activity of anion $[Ce(III)(HL^{Me})_2]^-$ in catalytic use of cobaltocenium salt **10**, generated in the presence of excess cobaltocene.

Entry	Cat.	[b][*rac*-LA]:[Cat.]:[4-MeBnOH]	T, °C	Duration, min	[c]Conversion, %
[a]1	$[CoCp_2]$	1000:5:3	120	30	10
[a]2	$[CoCp_2]$	1000:5:3	120	60	12
[a]3	$[CoCp_2]$	1000:10:3	80	30	5
[a]4	$[CoCp_2]$	1000:10:3	120	35	18
[a]5	$[CoCp_2]^+[PF_6]^-$	1000:2:3	80	30	0
[a]6	$[CoCp_2]^+[PF_6]^-$	1000:2:3	120	35	0

[a] Conditions: 500±5 mg *rac*-LA in 500 µL anhydrous PhCl dosed as stock solution of Cat. + 4-MeBnOH, vessel placed in aluminium heating block with thermocouple pre-heated to temperature T, magnetic stirring. [b] Molar ratio. [c] Conversion determined *via* ^1H NMR spectroscopy, by integration of LA and PLA methine resonances.

Table S10. Polymerisation data corresponding to application, variously, of **1** – **7** to the ROP of *rac*-LA under dilute solution phase conditions in chlorobenzene, variously at 60˚C and 90˚C, according to the catalyst present.

Entry	Cat.	[LA], mol dm^{-3}	[*rac*-LA]:[Cat.]:[4-MeBnOH]	T, ˚C	Duration, min	[b]Conversion, %	[c]P_r	[d]M_n^{Theo}, g mol^{-1}	[e]M_n^{SEC}, g mol^{-1}	[e]M_w^{SEC}, g mol^{-1}	[e]$Đ_M$
[a]1	**1**	1.16	500:1:3	90	2880	39	0.81	9490	N/A	N/A	N/A
[a]2	**2**	1.16	500:1:3	90	2880	31	0.76	7569	N/A	N/A	N/A
[a]3	**3**	1.16	500:1:3	90	2880	85	0.65	20540	23750	37850	1.59
[a]4	**4**	1.16	500:1:3	60	2880	25	0.57	6127	N/A	N/A	N/A
[a]5	**5**	1.16	500:1:3	60	2880	82	0.62	19819	15250	21350	1.40
[a]6	**6**	1.16	500:1:3	60	1200	97	0.55	23422	29700	46350	1.56
[a]7	**7**	1.16	500:1:3	60	1200	95	0.53	22942	28750	43600	1.52
[a]8	**4**	1.63	500:1:3	60	2760	75	0.62	18138	23800	26900	1.13
[a]9	**5**	1.63	500:1:3	60	2760	98	0.55	23663	24050	48150	2.00

[a] Conditions: 2000±5 mg *rac*-LA in anhydrous PhCl dosed as stock solution of Cat. + 4-MeBnOH, in a sealed J Young ampoule, placed in a pre-heated, stirred silicone oil bath (thermocouple), with magnetic stirring. [b] Conversion determined *via* ^{1}H NMR spectroscopy, by integration of LA and PLA methine resonances. [c] P_r calculated *via* polymer microstructure analysis (^{1}H{^{1}H} NMR); P_r = √(2-[*sis*]).[11] [d] M_n^{Theo} assuming an immortal kinetic regime with a catalyst bearing no functional initiating group, calculated from conversion and alcohol concentration: $M_n^{Theo} = \left\{\left(M_{r,LA} \times \frac{\%_{conv}}{100} \times \frac{[LA]}{[4-MeBnOH]}\right) + M_{r,4-MeBnOH}\right\}$. [e] Determined *via* size-exclusion chromatography (SEC) in THF, using a refractive index detector calibrated against polystyrene standards of known molecular weight.

Table S11. Polymerisation data corresponding to application of **7** to the ROP of *L*-LA under high-temperature, solvent-free conditions.

Entry	LA	[*rac*-LA]:[Cat.]:[4-MeBnOH]	T, ˚C	Duration, min	[b]Conversion, %	[c]P_r	[d]M_n^{Theo}, g mol^{-1}	[e]M_n^{SEC}, g mol^{-1}	[e]M_w^{SEC}, g mol^{-1}	[e]$Đ_M$
[a]1	*L*	48250:1:100	180	60	44	~0	30709	41250	57650	1.40
[a]2	*L*	48250:1:100	180	120	50	~0	34880	44650	65600	1.47
[a]3	*L*	18700:1:39	180	30	54	~0	37661	46350	69700	1.50
[a]4	*L*	18700:1:39	180	60	57	~0	39746	49850	74750	1.50
[a]5	*rac*	18700:1:39	180	15	62	0.62	43222	43750	70650	1.61
[a]6	*rac*	18700:1:39	180	30	69	0.60	48088	49850	79350	1.59
[a]7	*rac*	18700:1:39	180	60	73	0.62	50869	51600	86250	1.67
[a]8	*rac*	18700:1:39	180	120	80	0.60	55735	58000	94100	1.62

[a] Conditions: 10 g LA, **7** and 4-MeBnOH introduced as a solution (280 µL, 12.5% wt/vol 4-MeBnOH) in PhCl, otherwise solvent-free, in a sealed J Young ampoule. The ampoule was placed in a pre-heated, stirred silicone oil bath (thermocouple set temperature 180˚C), with magnetic stirring. Time taken to attain a constant temperature is assumed to be negligible. [b] Conversion determined *via* ^{1}H NMR spectroscopy, by integration of LA and PLA methine resonances. [c] P_r calculated *via* polymer microstructure analysis (^{1}H{^{1}H} NMR); P_r = √(2-[*sis*]).[11] [d] M_n^{Theo} assuming an immortal kinetic regime with a catalyst bearing no functional initiating group, calculated from conversion and alcohol concentration: $M_n^{Theo} = \left\{\left(M_{r,LA} \times \frac{\%_{conv}}{100} \times \frac{[LA]}{[4-MeBnOH]}\right) + M_{r,4-MeBnOH}\right\}$. [e] Determined *via* size-exclusion chromatography (SEC) in THF, using a refractive index detector calibrated against polystyrene standards of known molecular weight.

S7. Kinetic data and associated structural and polymer characterisation data

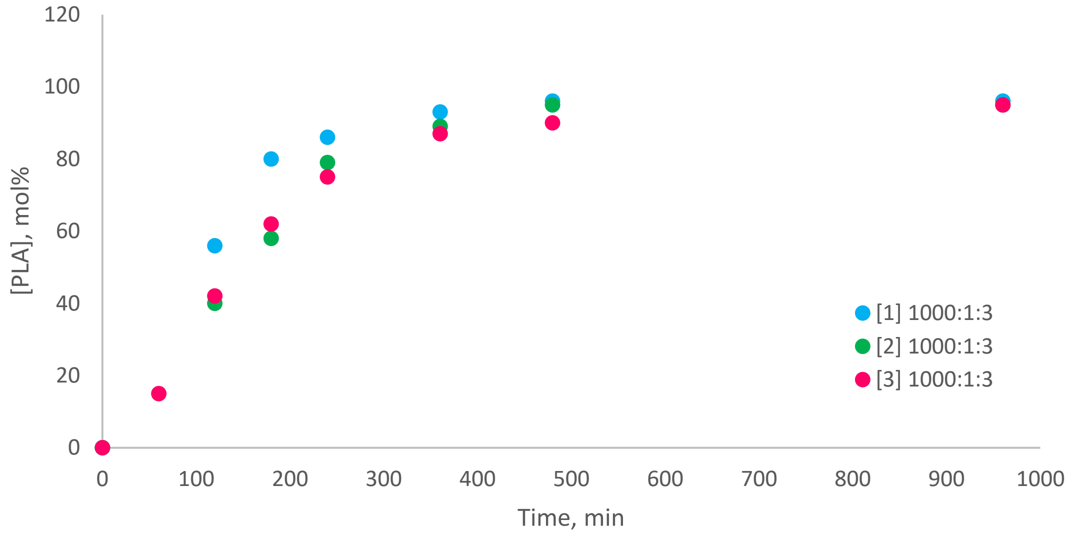

Figure S13. Plots of conversion *versus* time for the ROP of *rac*-LA at 120°C in PhCl (50% wt/vol), in the presence of 0.1 mol% catalysts **1**, **2** and **3**, respectively, and 0.3 mol% 4-MeBnOH.

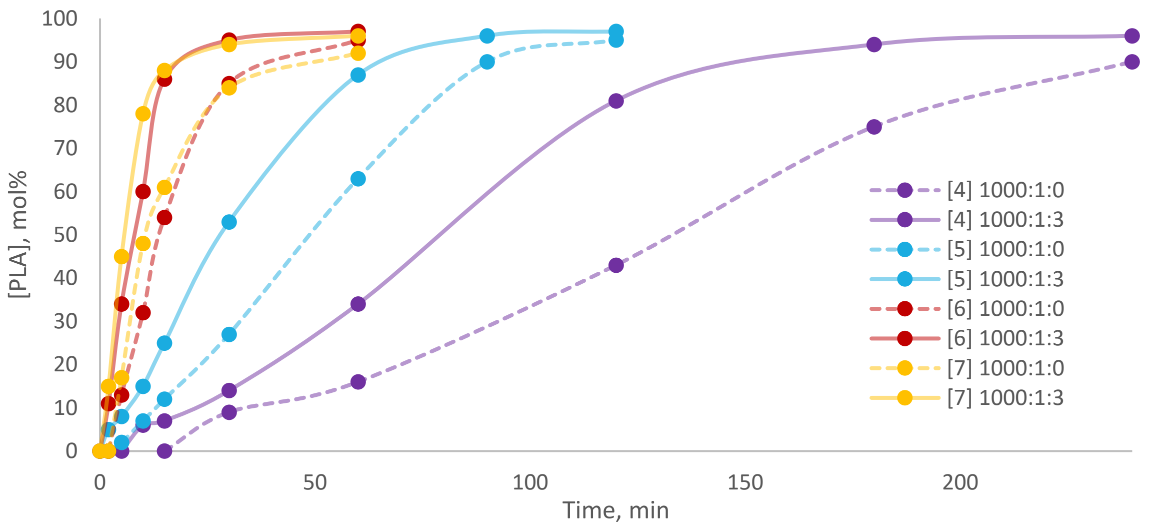

Figure S14. Plots of conversion *versus* time for the ROP of *rac*-LA at 120°C in PhCl (50% wt/vol), in the presence of 0.1 mol% catalysts **4**, **5**, **6** and **7**, respectively, and in the presence and absence, respectively, of 0.3 mol% 4-MeBnOH.

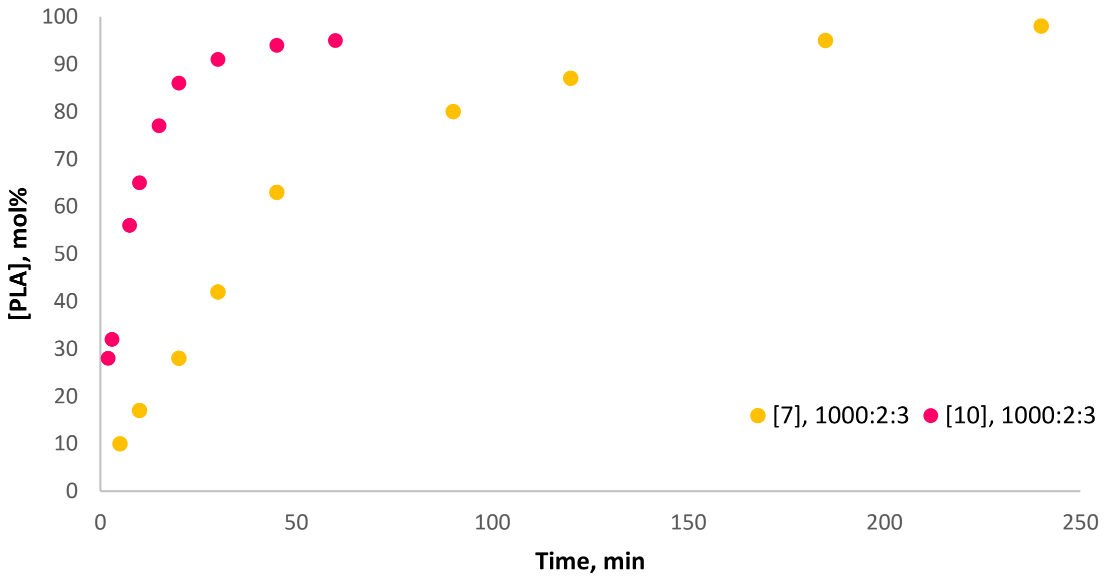

Figure S15. Plots of conversion *versus* time for the ROP of *rac*-LA at 80˚C in PhCl (50% wt/vol), in the presence of 0.1 mol% catalysts **7** and **10** , respectively, and in the presence of 0.3 mol% 4-MeBnOH. **10** was generated *in-situ via* the reduction of **3** with cobaltocene.

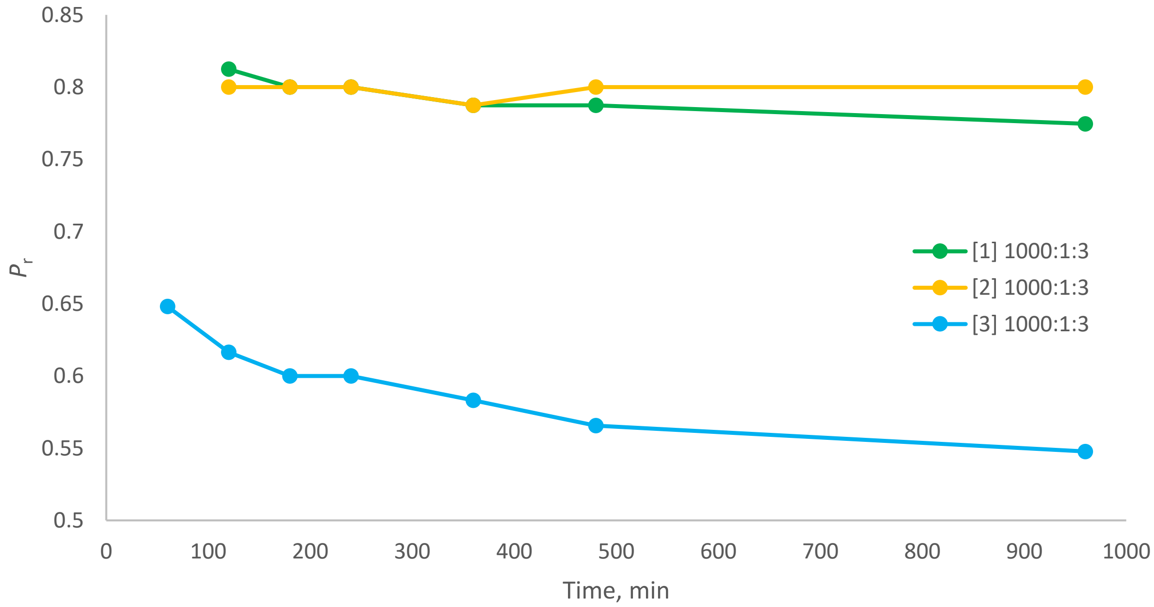

Figure S16. Plots of P_r *versus* time for the ROP of *rac*-LA at 120˚C in PhCl (50% wt/vol), in the presence of 0.1 mol% catalysts **1**, **2** and **3**, respectively, and 0.3 mol% 4-MeBnOH.

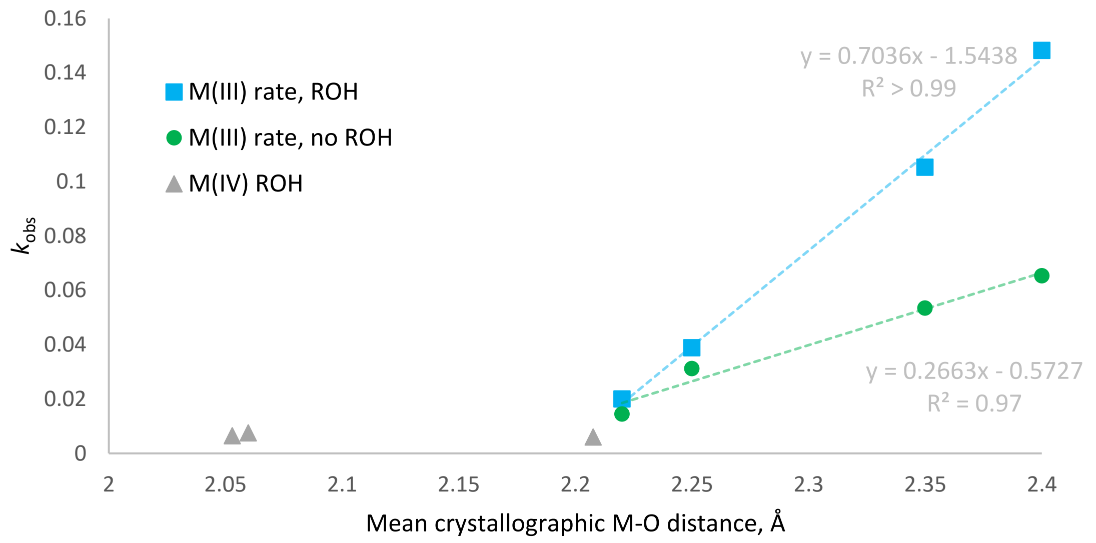

Figure S17. Plot of observed rate constant, k_{obs}, for the ROP of *rac*-LA *versus* mean crystallographic metal-oxygen bond distance for catalysts **1** – **7** in the presence of exogenous 4-MeBnOH at 120˚C in PhCl, and catalysts **4** – **7** in the absence of 4-MeBnOH at 120˚C in PhCl. Data reproduced from main text.

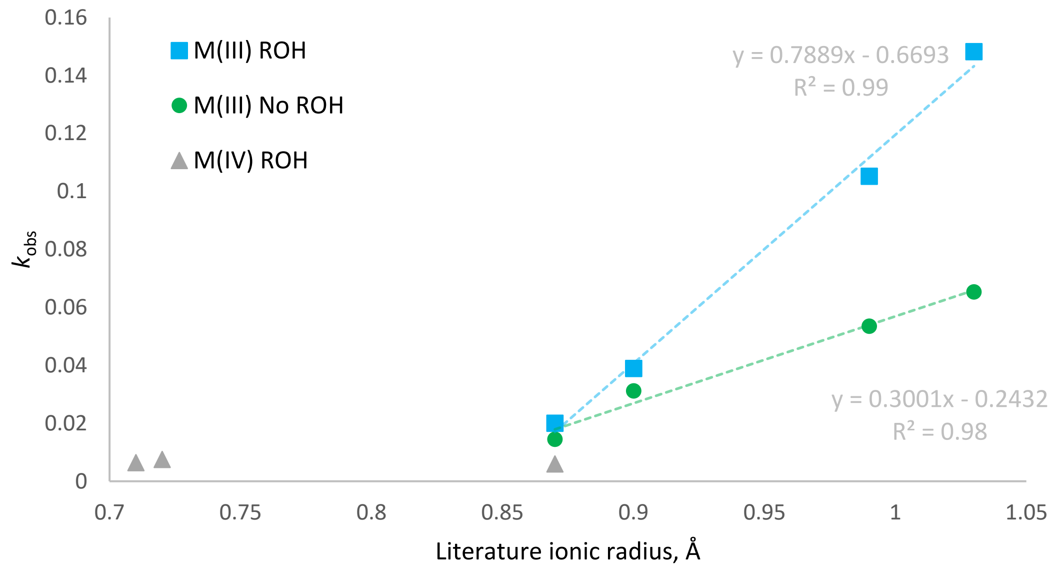

Figure S18. Plot of observed rate constant, k_{obs}, for the ROP of *rac*-LA *versus* literature values for the ionic radius of the relevant metal centre for catalysts **1** – **7** in the presence of exogenous 4-MeBnOH at 120˚C in PhCl, and catalysts **4** – **7** in the absence of 4-MeBnOH at 120˚C in PhCl.[12]

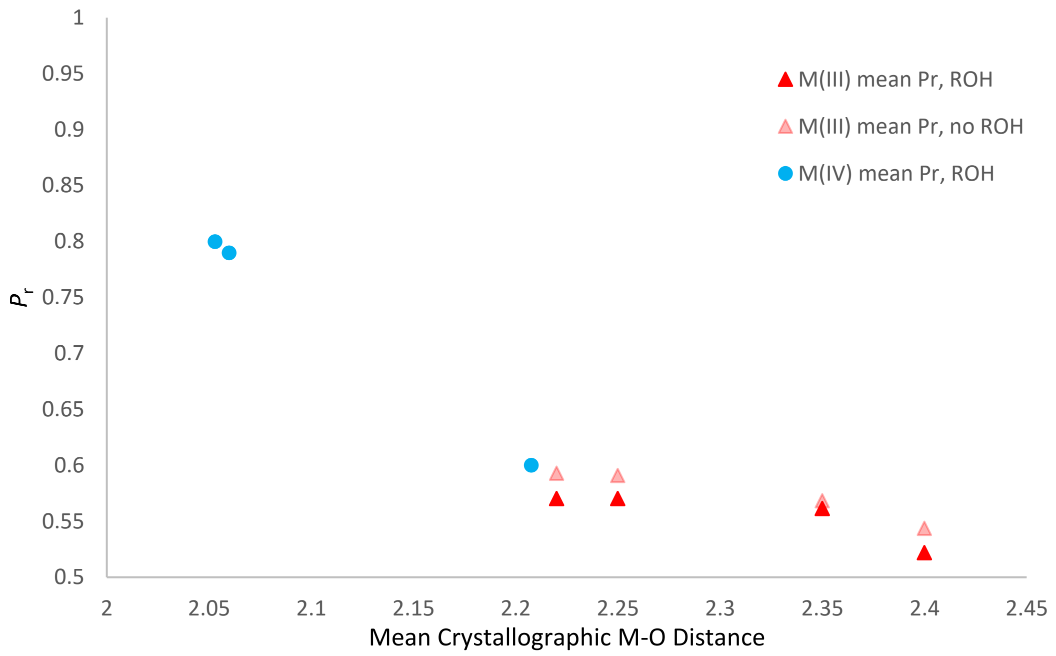

Figure S19. Plot of mean P_r of polymer samples produced in the course of *ex-situ* kinetic studies of the ROP of *rac*-LA *versus* mean crystallographic metal-oxygen bond distance for catalysts **1** – **7** in the presence of exogenous 4-MeBnOH at 120˚C in PhCl, and catalysts **4** – **7** in the absence of 4-MeBnOH at 120˚C in PhCl.

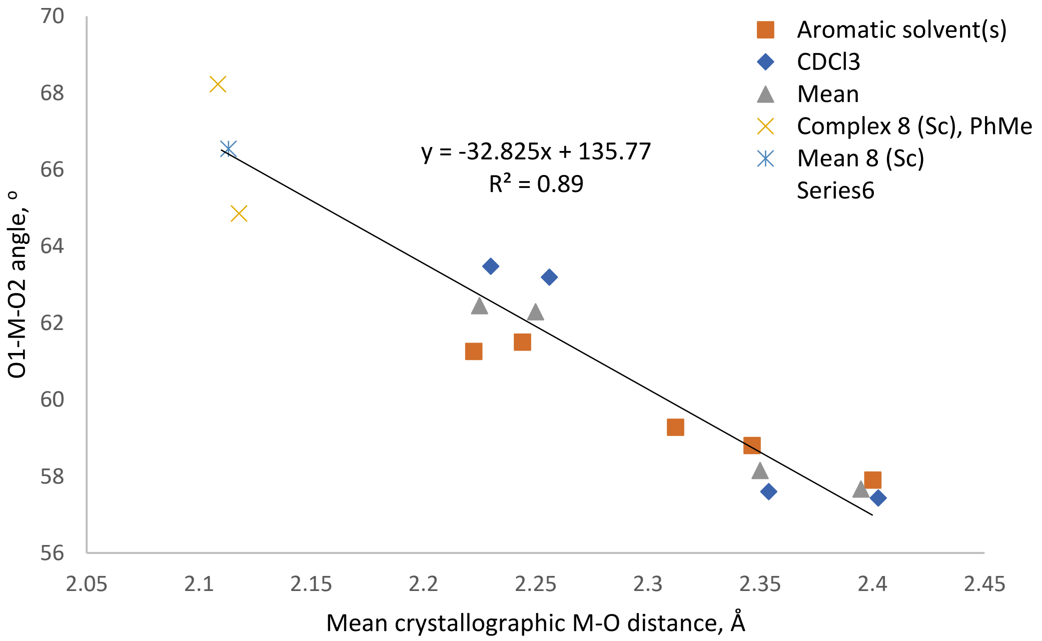

Figure S20. Plots of O1-M-O2 angle *versus* mean crystallographic metal-oxygen bond distance for trivalent metal compounds **4** – **9**. Where two distinct solid-state structures of a compound have been obtained (**4**, **5**, **6**, **7**), containing different solvents in the unit cell, there is associated variation in the structural parameters. Both sets of values are represented, grouped into distinct series according to the type of solvent present. In such cases, the mean value for the two distinct polymorphs is also represented. Similarly, the solid-state structure of **8** contained two molecules of the compound within the unit cell, with distinct geometries. Parameters for both molecules have been included here, and a mean value is shown for clarity. The trend line has been constructed using all experimental values (compounds **4** – **9**, all polymorphs) with exclusion of mean values.

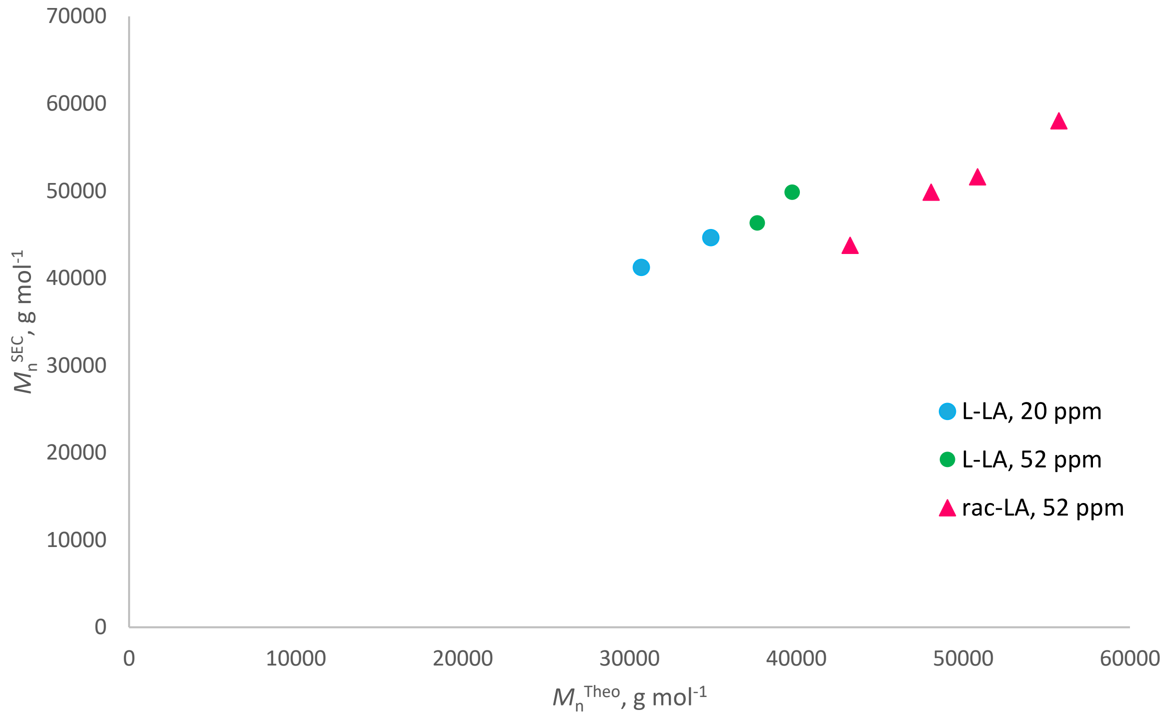

Figure S21. Plots of M_n^{SEC} *versus* M_n^{Theo} for the ROP of *L*-LA and *rac*-LA, respectively, in the presence of 20 – 52 ppm (metal by weight, 0.0021 – 0.0053 mol%) **7** at 180˚C, solvent-free in the presence of a large excess of 4-MeBnOH, wherein M_n^{Theo} is calculated assuming quantitative initiation of polymer chains by 4-MeBnOH.

S8. Size Exclusion Chromatograms

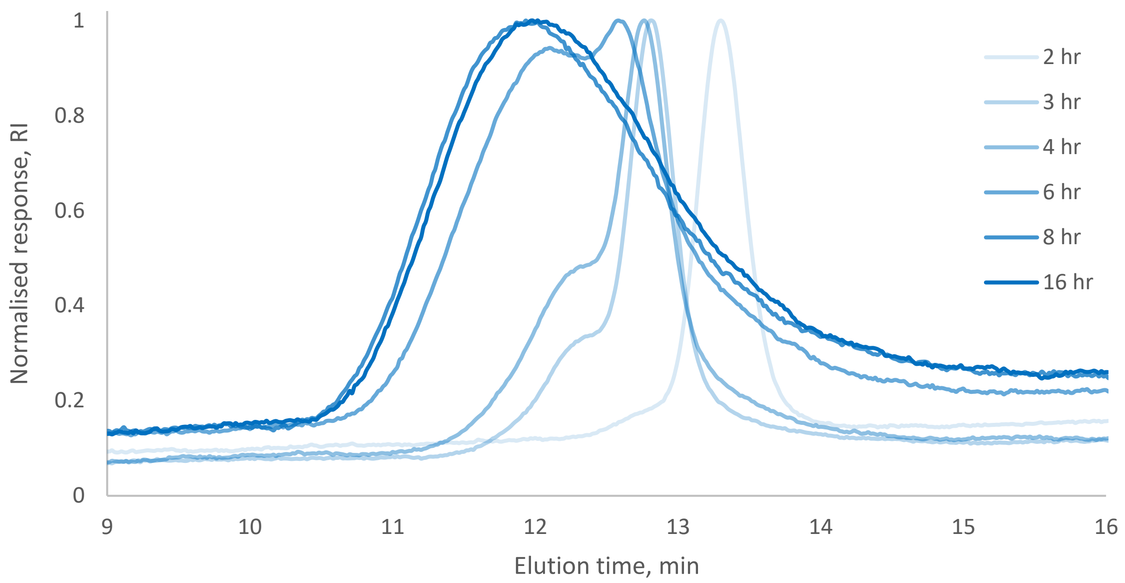

Figure S22. Stacked size exclusion chromatograms of polymer samples produced in the *ex-situ* kinetic study of the ROP of *rac*-LA at 120˚C in PhCl (50% wt/vol), in the presence of 0.1 mol% **1** and 0.3 mol% 4-MeBnOH.

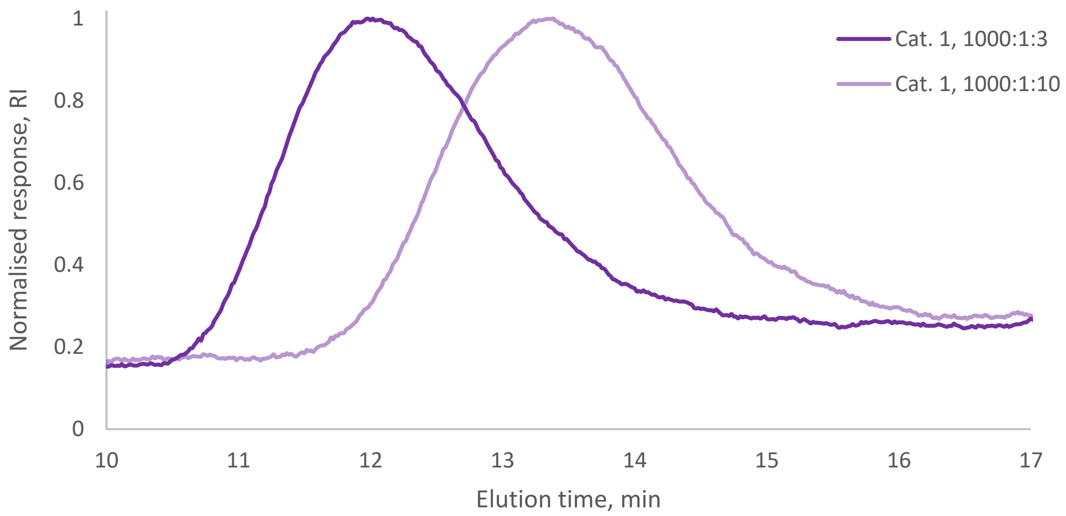

Figure S23. Stacked size exclusion chromatograms of polymer samples produced in the ROP of *rac*-LA at 120˚C in PhCl (50% wt/vol), in the presence of 0.1 mol% **1**, and 0.3 mol% and 1.0 mol% 4-MeBnOH, respectively.

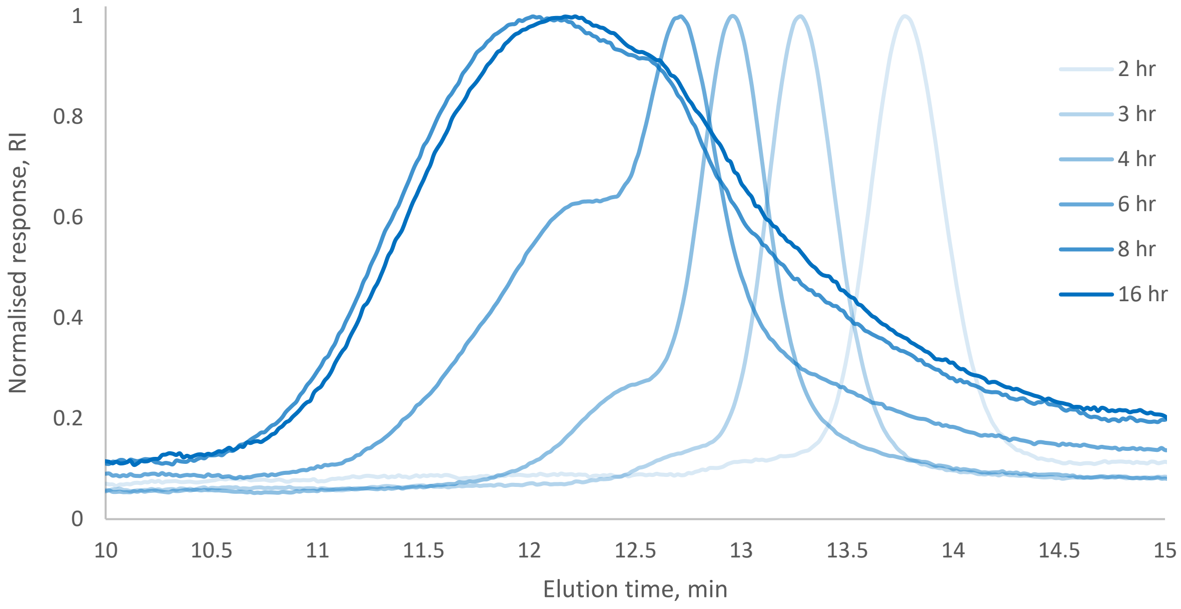

Figure S24. Stacked size exclusion chromatograms of polymer samples produced in the *ex-situ* kinetic study of the ROP of *rac*-LA at 120˚C in PhCl (50% wt/vol), in the presence of 0.1 mol% **2** and 0.3 mol% 4-MeBnOH.

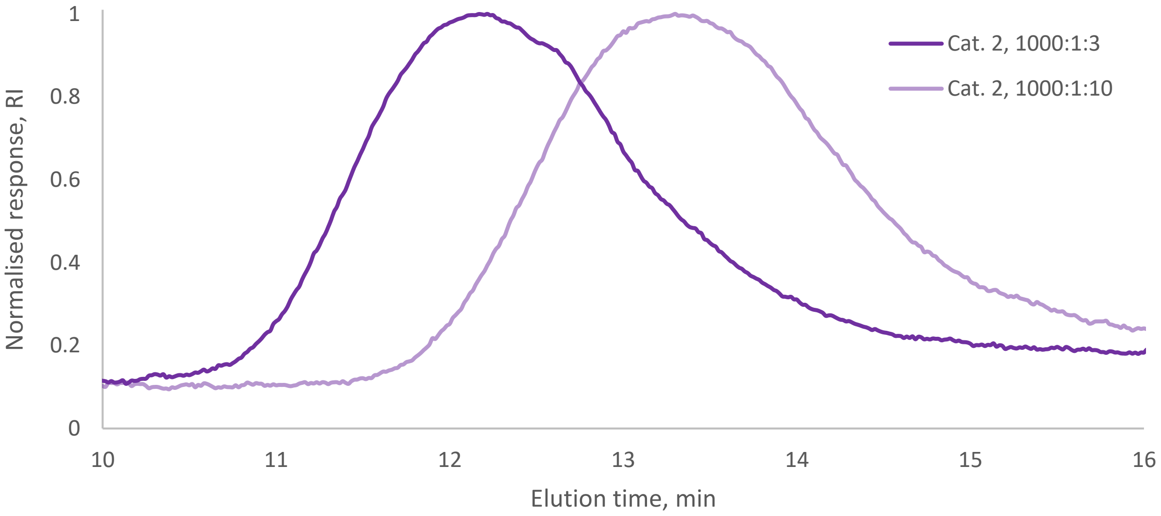

Figure S25. Stacked size exclusion chromatograms of polymer samples produced in the ROP of *rac*-LA at 120˚C in PhCl (50% wt/vol), in the presence of 0.1 mol% **2**, and 0.3 mol% and 1.0 mol% 4-MeBnOH, respectively.

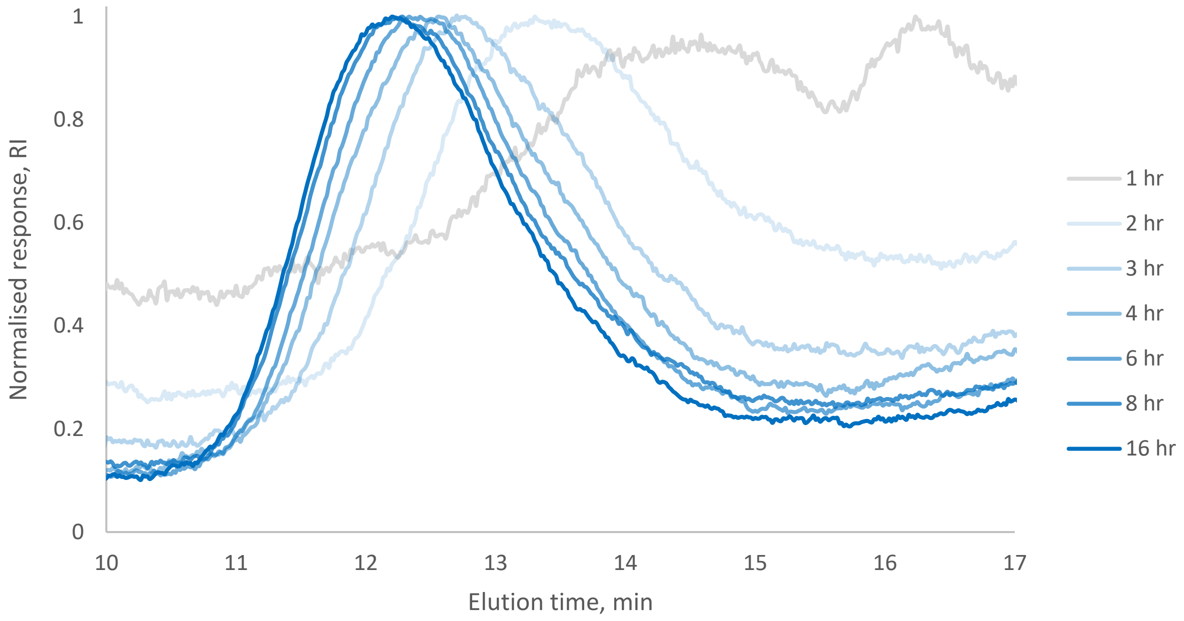

Figure S26. Stacked size exclusion chromatograms of polymer samples produced in the *ex-situ* kinetic study of the ROP of *rac*-LA at 120˚C in PhCl (50% wt/vol), in the presence of 0.1 mol% **3** and 0.3 mol% 4-MeBnOH.

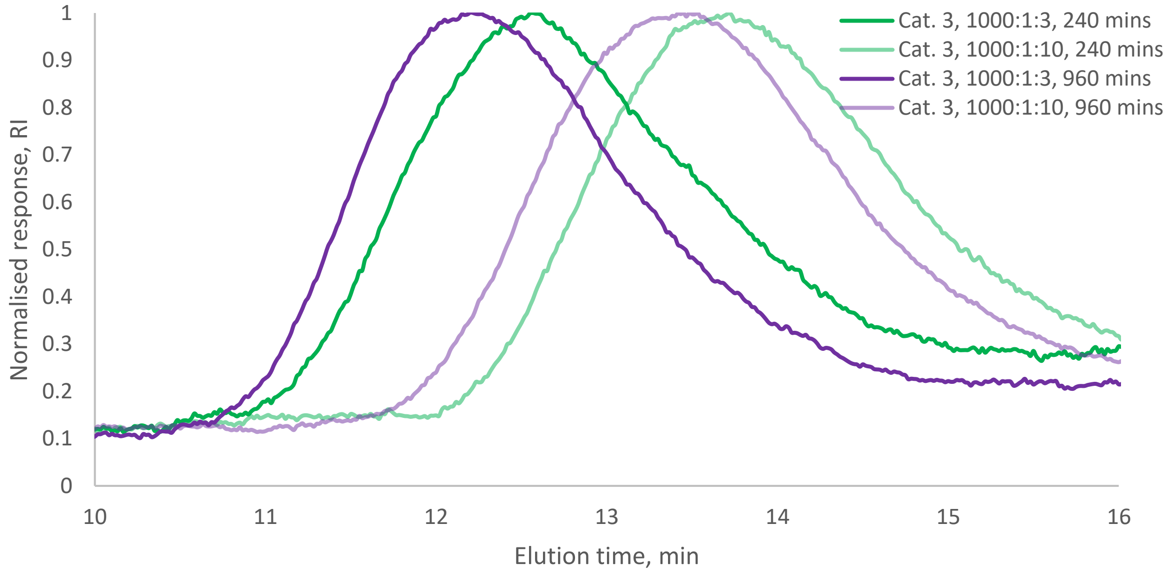

Figure S27. Stacked size exclusion chromatograms of polymer samples produced in the ROP of *rac*-LA at 120˚C in PhCl (50% wt/vol), in the presence of 0.1 mol% **3**, and 0.3 mol% and 1.0 mol% 4-MeBnOH, respectively, after reaction durations of 240 min and 960 min.

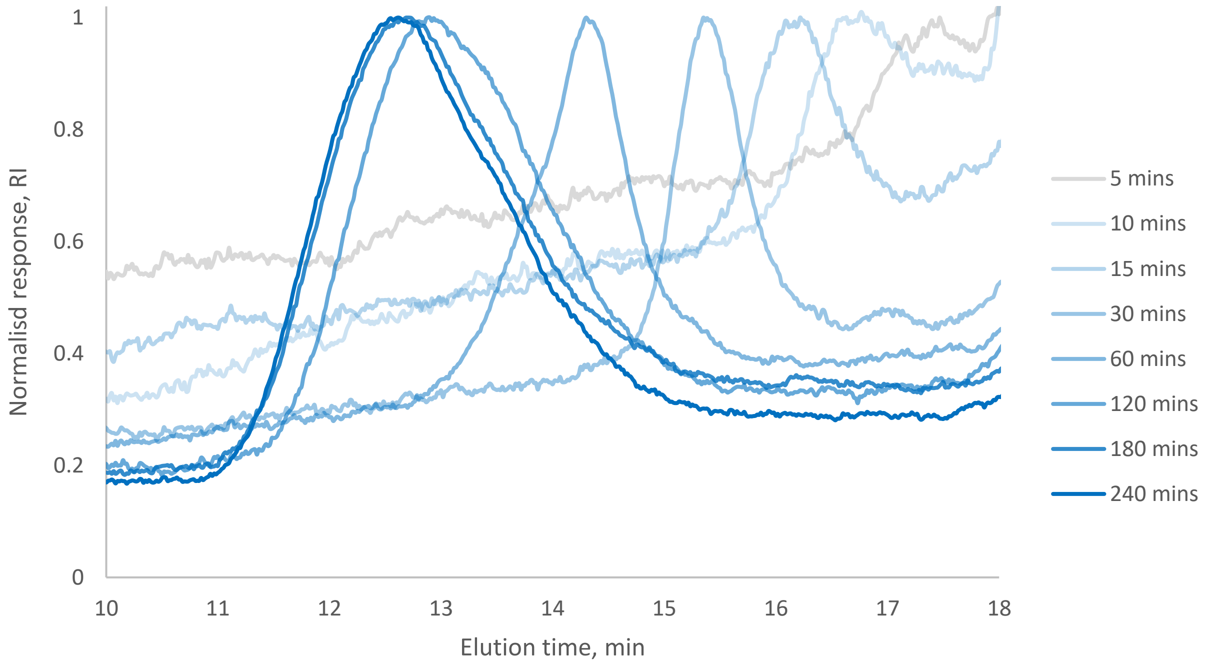

Figure S28. Stacked size exclusion chromatograms of polymer samples produced in the *ex-situ* kinetic study of the ROP of *rac*-LA at 120˚C in PhCl (50% wt/vol), in the presence of 0.1 mol% **4** and 0.3 mol% 4-MeBnOH.

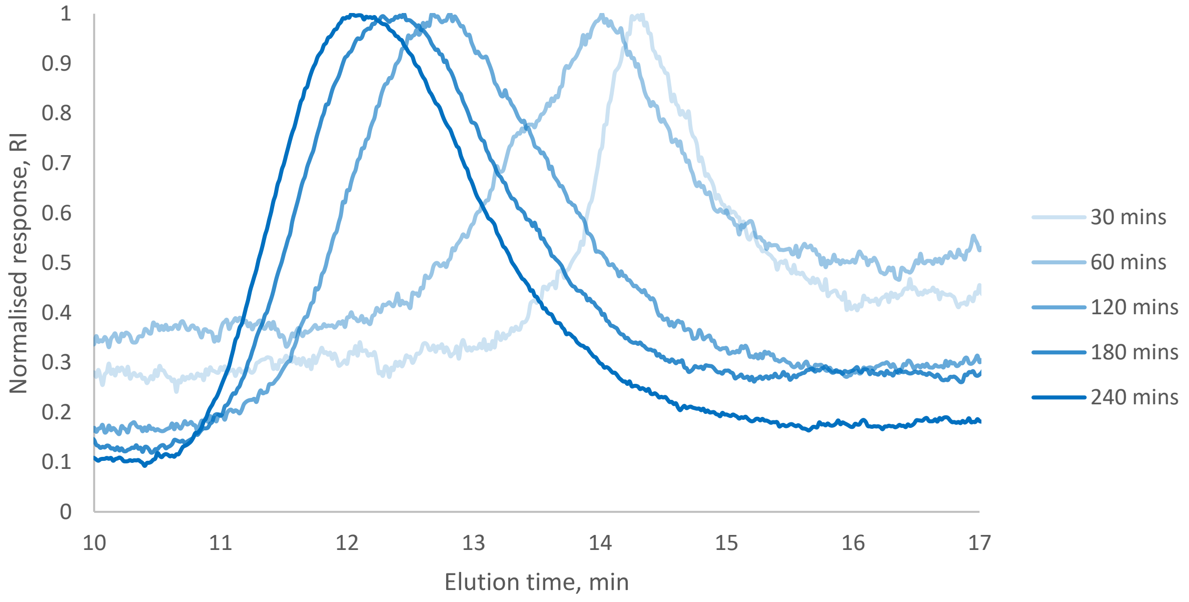

Figure S29. Stacked size exclusion chromatograms of polymer samples produced in the *ex-situ* kinetic study of the ROP of *rac*-LA at 120˚C in PhCl (50% wt/vol), in the presence of 0.1 mol% **4** and in the absence of 4-MeBnOH.

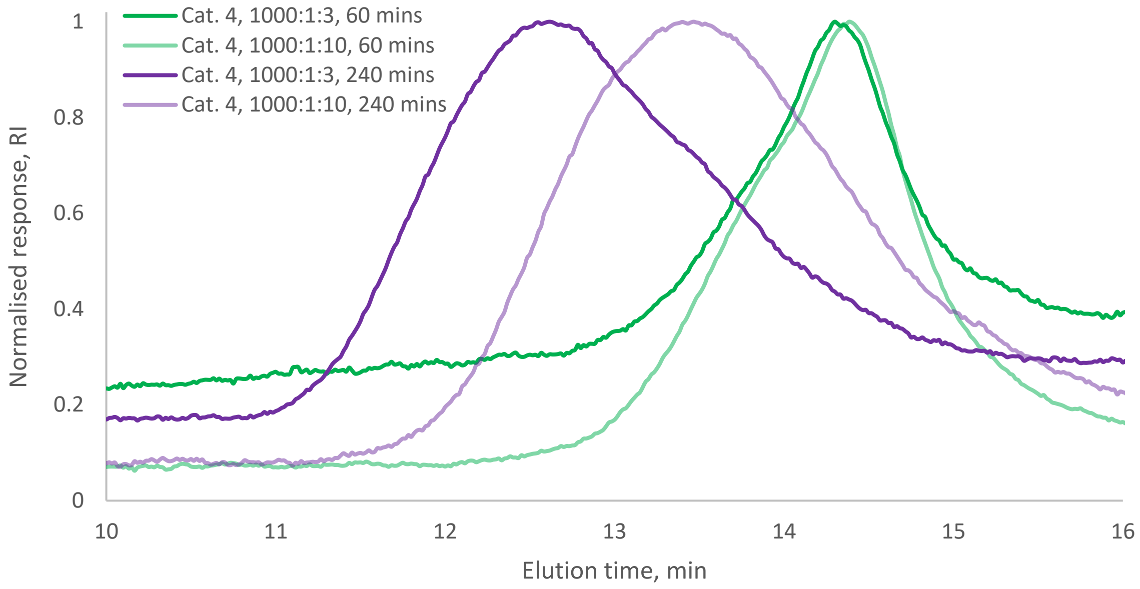

Figure S30. Stacked size exclusion chromatograms of polymer samples produced in the ROP of *rac*-LA at 120°C in PhCl (50% wt/vol), in the presence of 0.1 mol% **4**, and 0.3 mol% and 1.0 mol% 4-MeBnOH, respectively, after reaction durations of 60 min and 240 min.

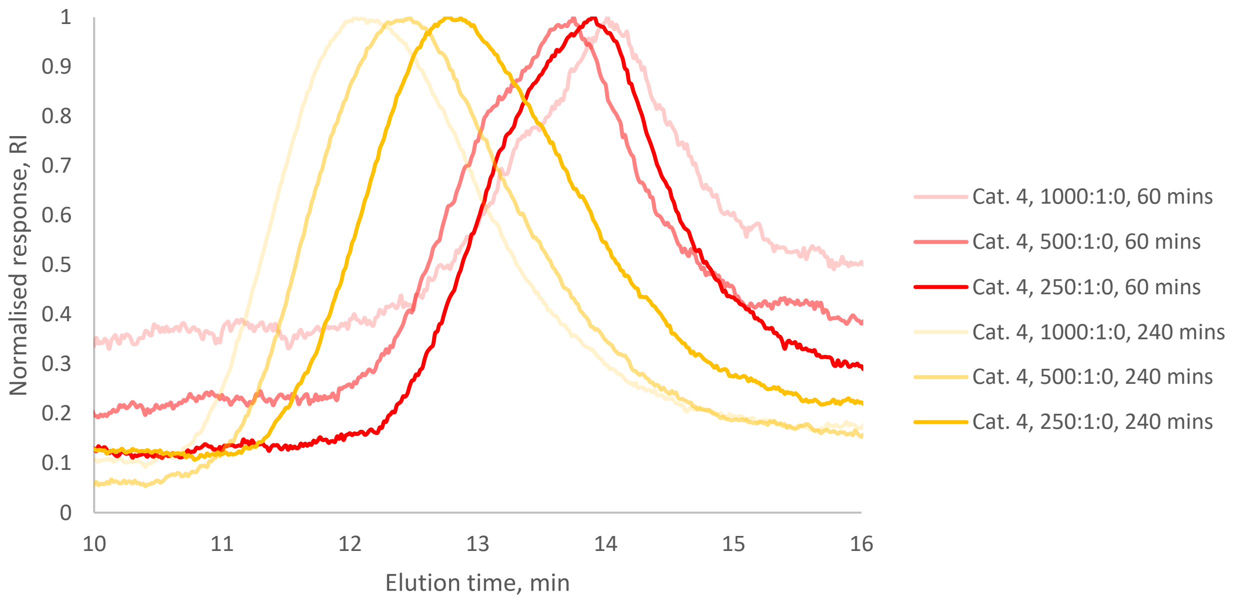

Figure S31. Stacked size exclusion chromatograms of polymer samples produced in the ROP of *rac*-LA at 120°C in PhCl (50% wt/vol), in the presence of various loadings of **4** (0.1 – 0.4 mol%), and in the absence of 4-MeBnOH, after reaction durations of 60 min and 240 min.

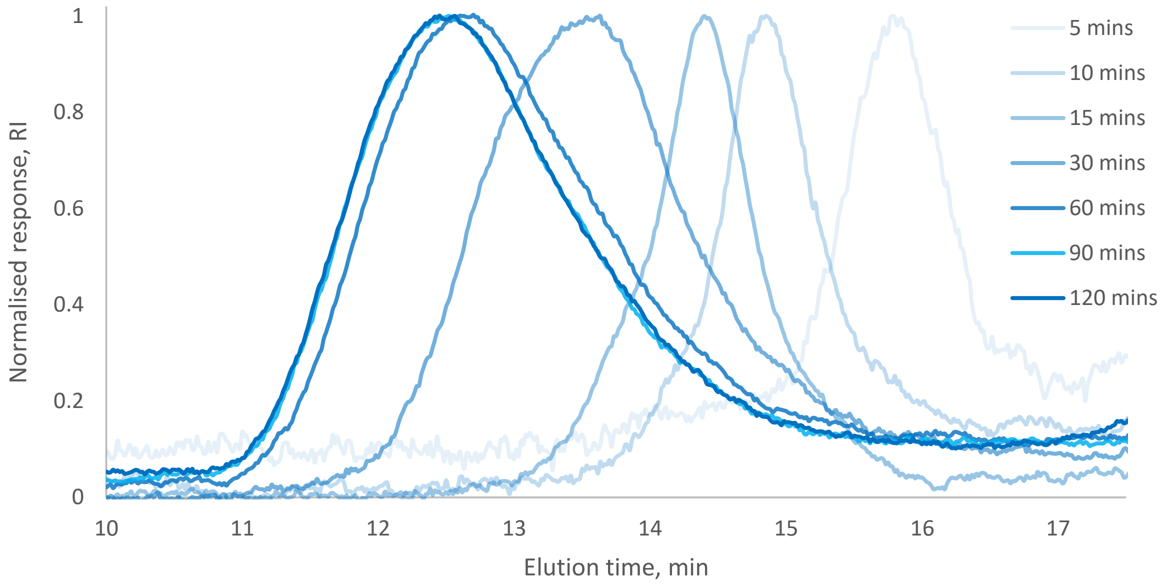

Figure S32. Stacked size exclusion chromatograms of polymer samples produced in the *ex-situ* kinetic study of the ROP of *rac*-LA at 120˚C in PhCl (50% wt/vol), in the presence of 0.1 mol% **5** and 0.3 mol% 4-MeBnOH.

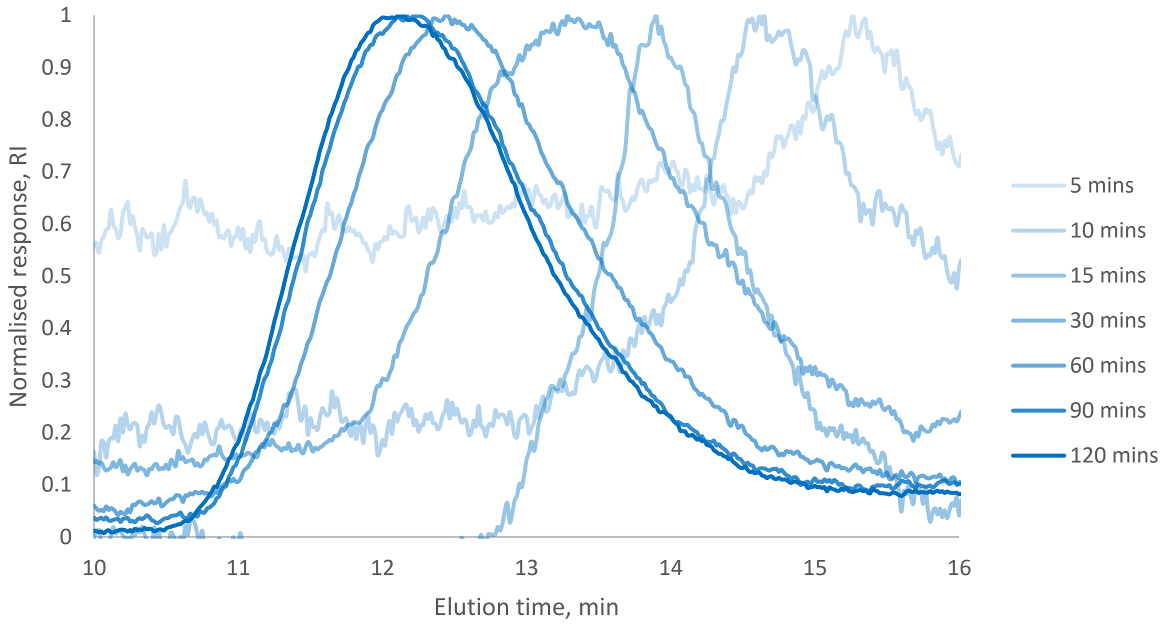

Figure S33. Stacked size exclusion chromatograms of polymer samples produced in the *ex-situ* kinetic study of the ROP of *rac*-LA at 120˚C in PhCl (50% wt/vol), in the presence of 0.1 mol% **5** and in the absence of 4-MeBnOH.

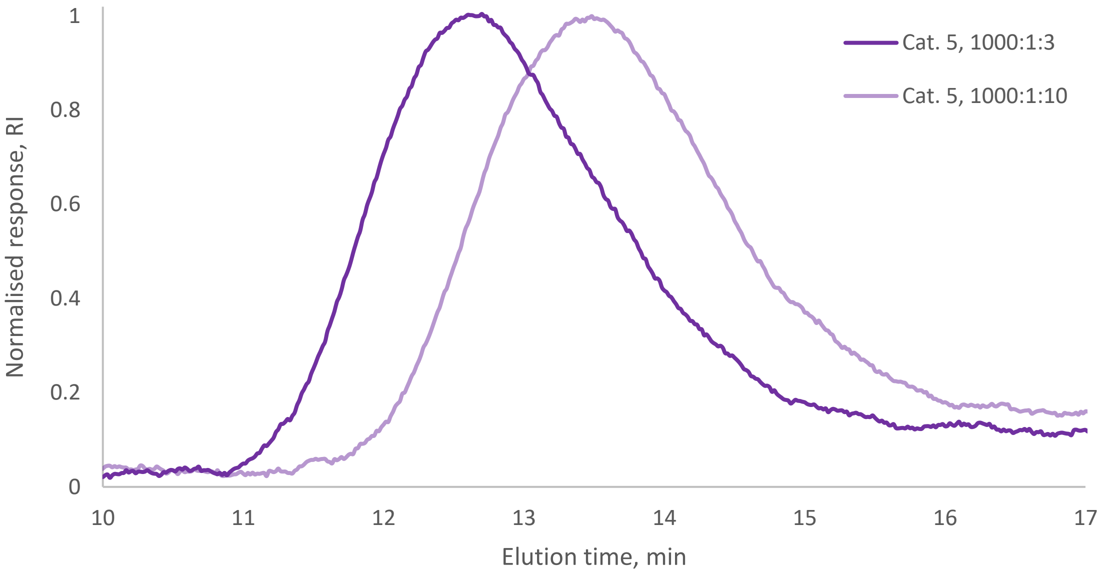

Figure S34. Stacked size exclusion chromatograms of polymer samples produced in the ROP of *rac*-LA at 120°C in PhCl (50% wt/vol), in the presence of 0.1 mol% **4**, and 0.3 mol% and 1.0 mol% 4-MeBnOH, respectively.

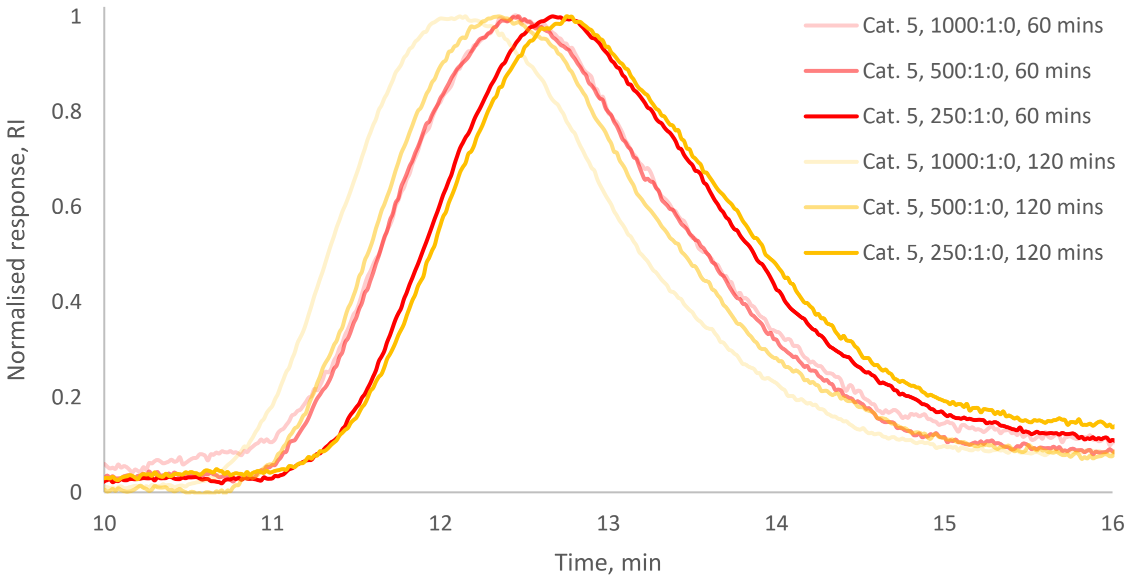

Figure S35. Stacked size exclusion chromatograms of polymer samples produced in the ROP of *rac*-LA at 120°C in PhCl (50% wt/vol), in the presence of various loadings of **5** (0.1 – 0.4 mol%), and in the absence of 4-MeBnOH, after reaction durations of 60 min and 120 min.

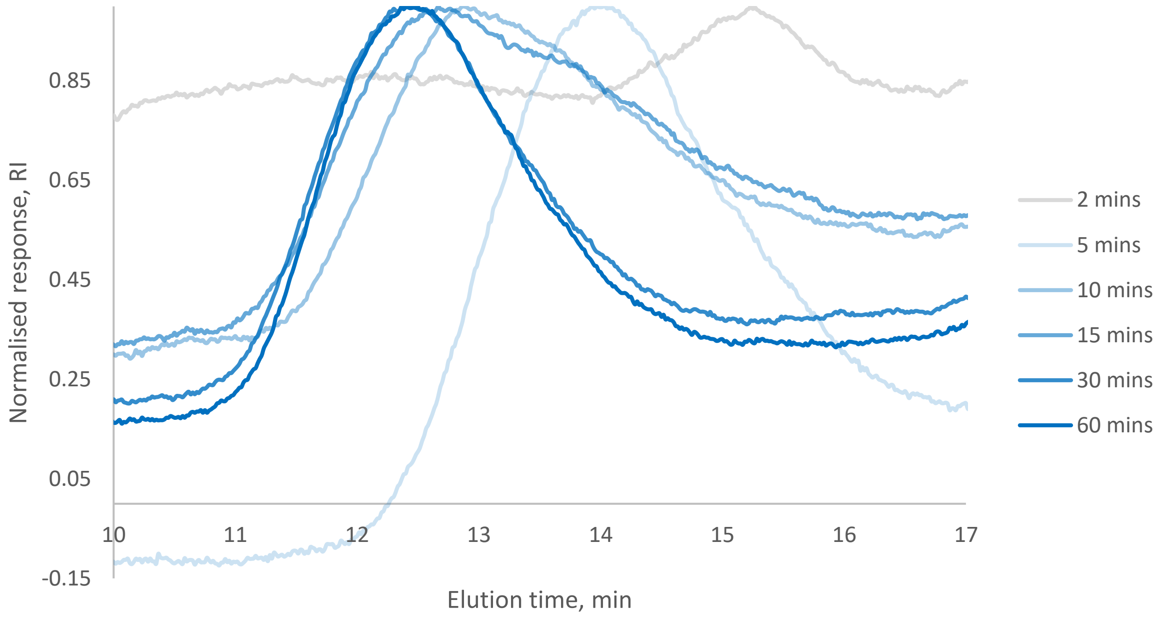

Figure S36. Stacked size exclusion chromatograms of polymer samples produced in the *ex-situ* kinetic study of the ROP of *rac*-LA at 120˚C in PhCl (50% wt/vol), in the presence of 0.1 mol% **6** and 0.3 mol% 4-MeBnOH.

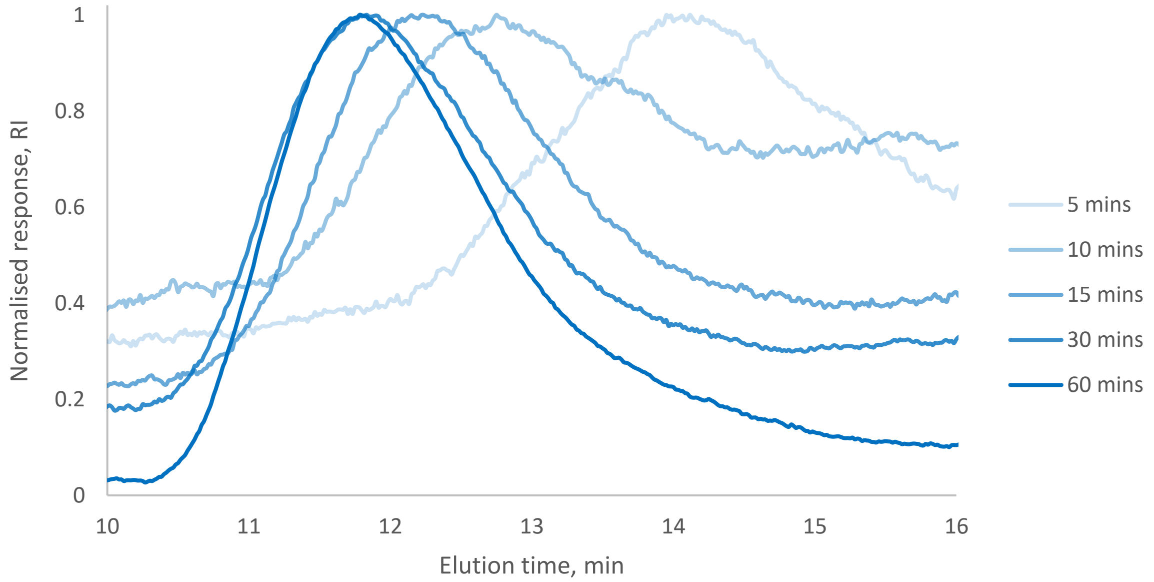

Figure S37. Stacked size exclusion chromatograms of polymer samples produced in the *ex-situ* kinetic study of the ROP of *rac*-LA at 120˚C in PhCl (50% wt/vol), in the presence of 0.1 mol% **6** and in the absence of 4-MeBnOH.

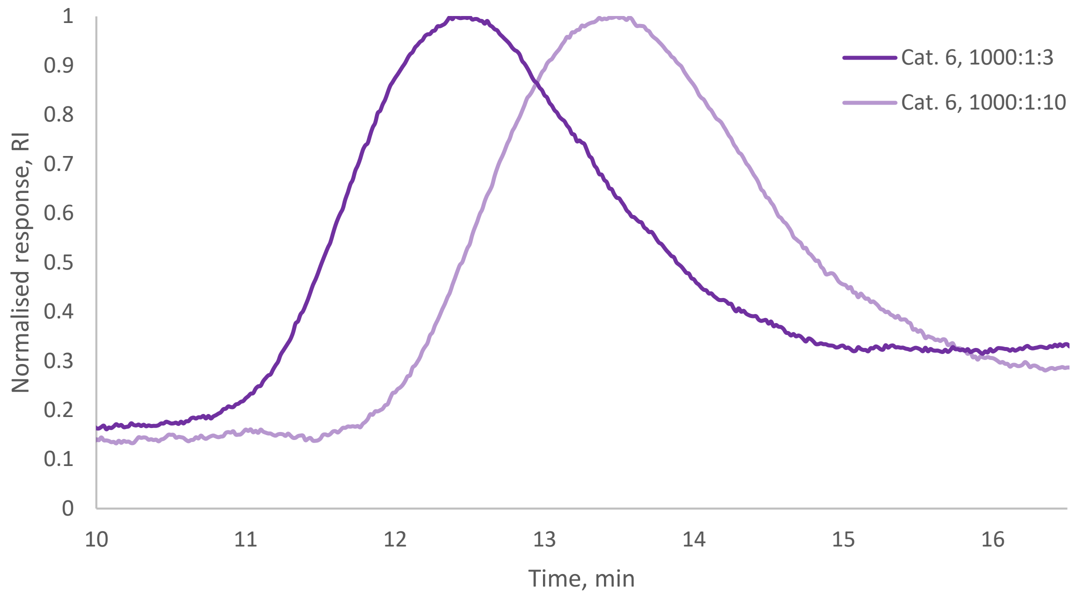

Figure S38. Stacked size exclusion chromatograms of polymer samples produced in the ROP of *rac*-LA at 120°C in PhCl (50% wt/vol), in the presence of 0.1 mol% **6**, and 0.3 mol% and 1.0 mol% 4-MeBnOH, respectively.

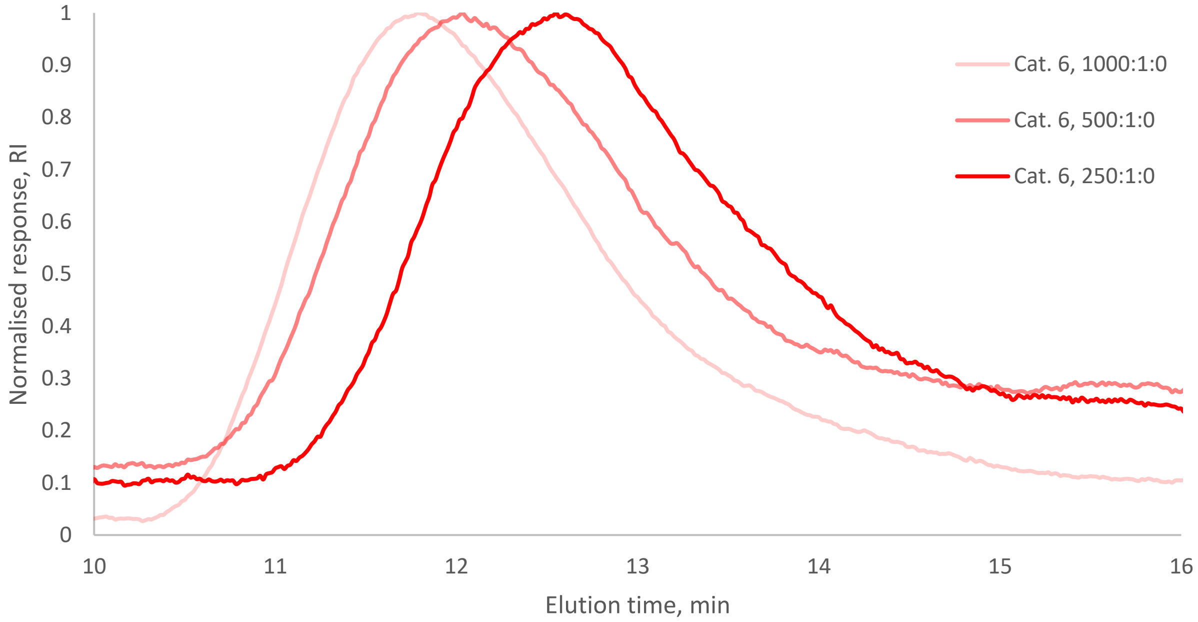

Figure S39. Stacked size exclusion chromatograms of polymer samples produced in the ROP of *rac*-LA at 120°C in PhCl (50% wt/vol), in the presence of various loadings of **6** (0.1 – 0.4 mol%), and in the absence of 4-MeBnOH.

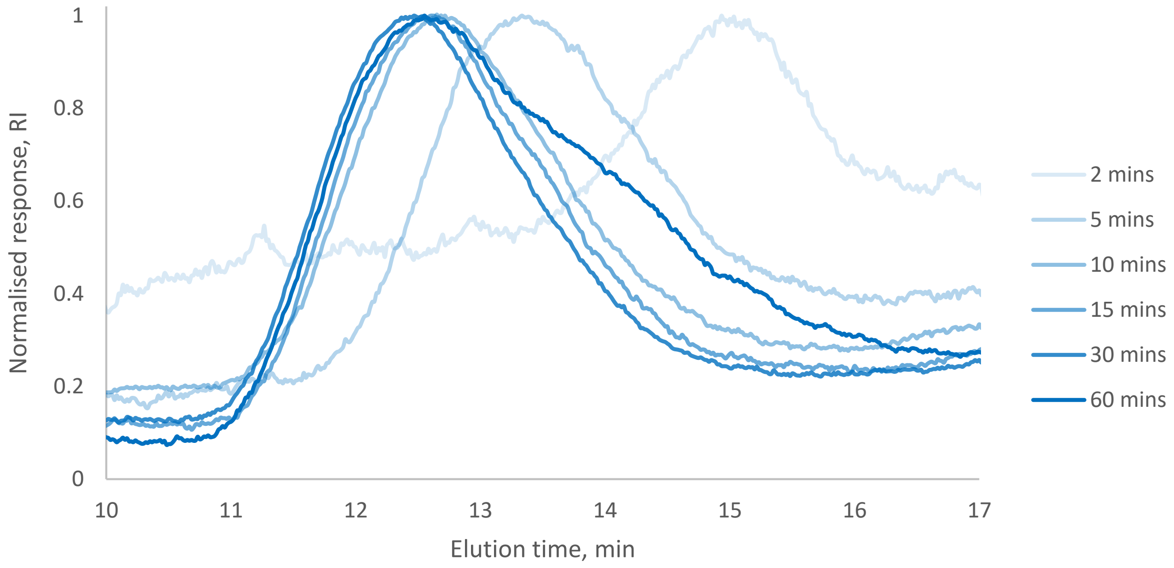

Figure S40. Stacked size exclusion chromatograms of polymer samples produced in the *ex-situ* kinetic study of the ROP of *rac*-LA at 120˚C in PhCl (50% wt/vol), in the presence of 0.1 mol% **7** and 0.3 mol% 4-MeBnOH.

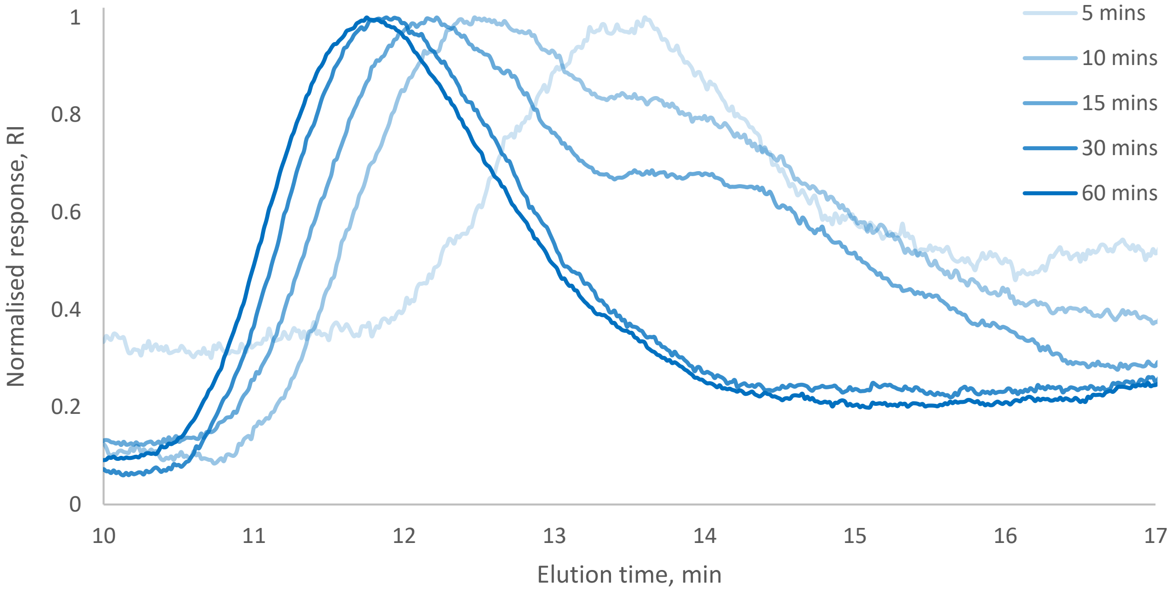

Figure S41. Stacked size exclusion chromatograms of polymer samples produced in the *ex-situ* kinetic study of the ROP of *rac*-LA at 120˚C in PhCl (50% wt/vol), in the presence of 0.1 mol% **7** and in the absence of 4-MeBnOH.

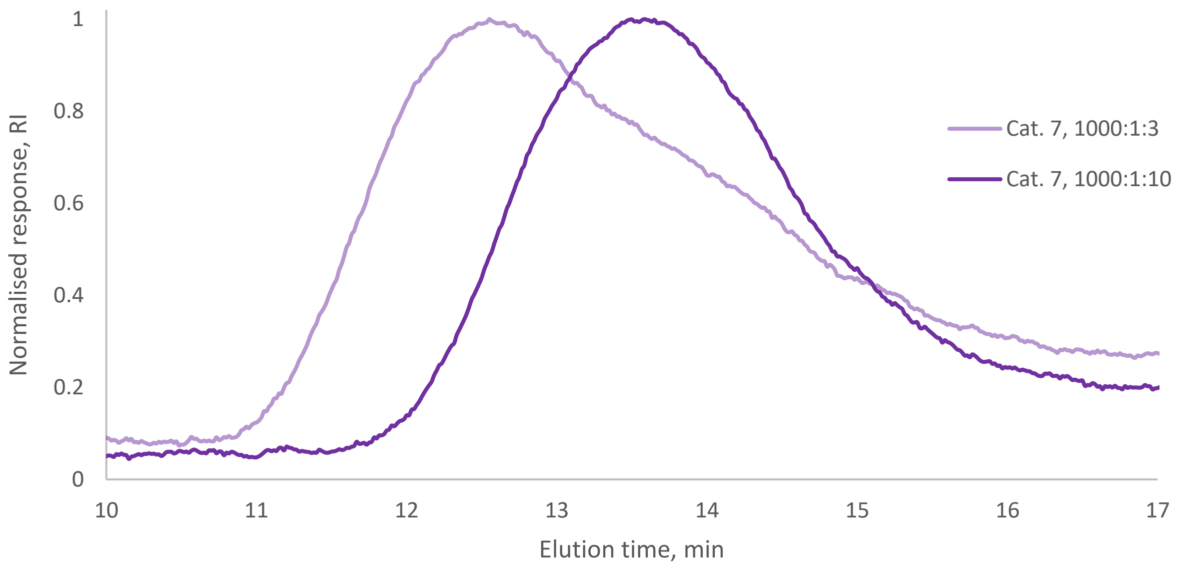

Figure S42. Stacked size exclusion chromatograms of polymer samples produced in the ROP of *rac*-LA at 120˚C in PhCl (50% wt/vol), in the presence of 0.1 mol% **7**, and 0.3 mol% and 1.0 mol% 4-MeBnOH, respectively.

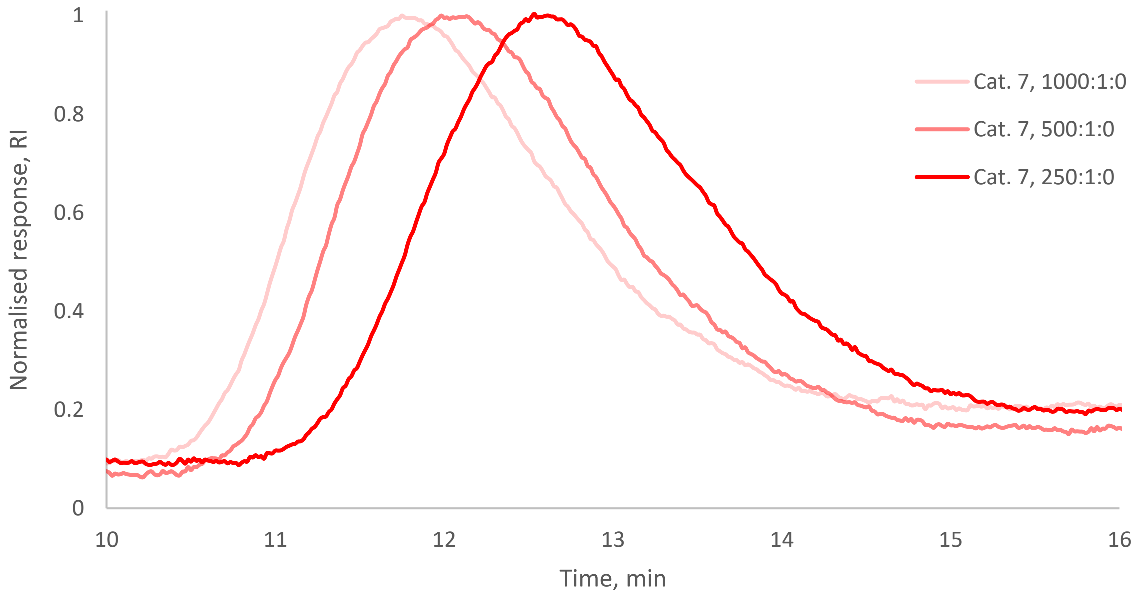

Figure S43. Stacked size exclusion chromatograms of polymer samples produced in the ROP of *rac*-LA at 120˚C in PhCl (50% wt/vol), in the presence of various loadings of **7** (0.1 – 0.4 mol%), and in the absence of 4-MeBnOH.

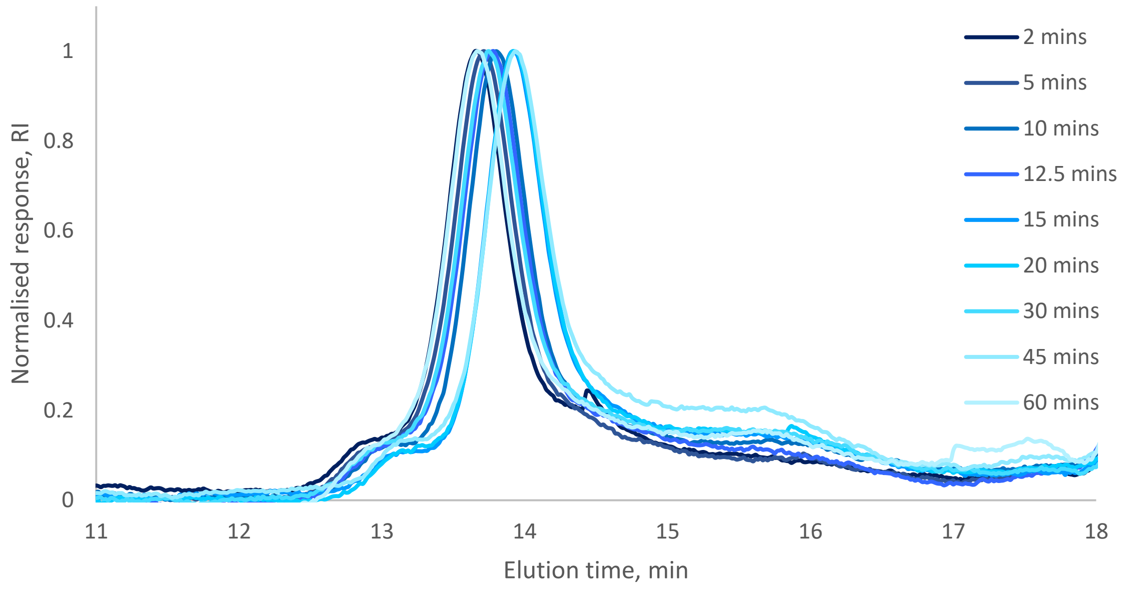

Figure S44. Stacked size exclusion chromatograms of polymer samples produced in the *ex-situ* kinetic study of the ROP of *rac*-LA at 120°C in PhCl (50% wt/vol), in the presence of 0.1 mol% **10** (generated *in situ via* the reduction of **3** in the presence of excess cobaltocene) and 0.3 mol% 4-MeBnOH.

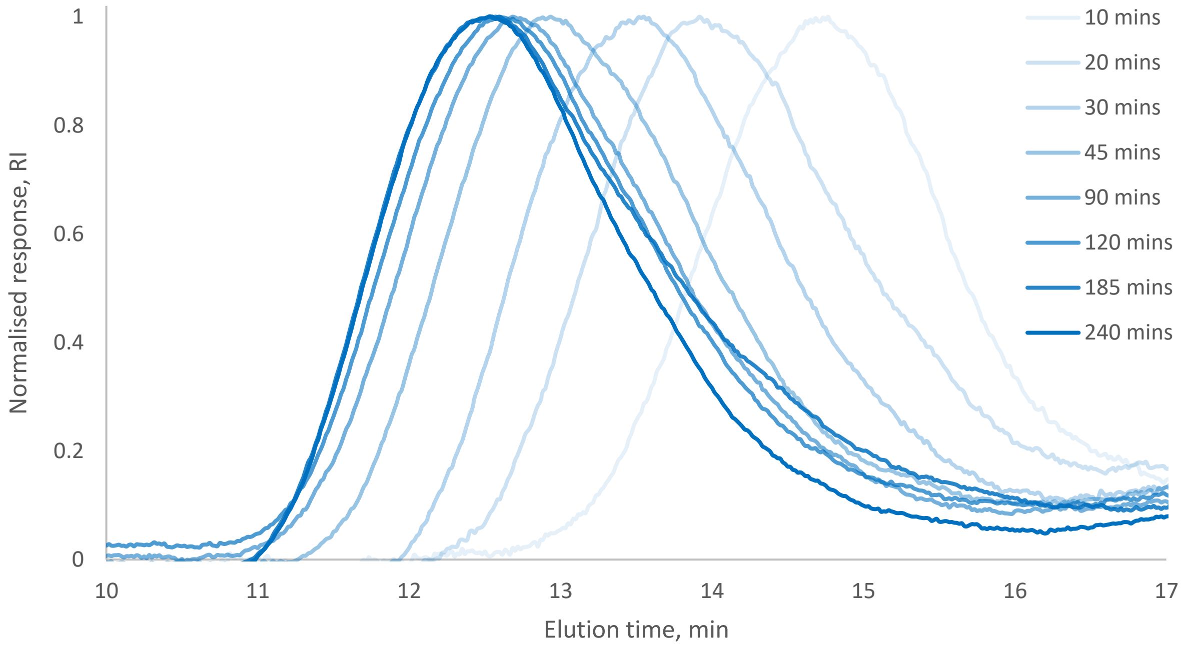

Figure S45. Stacked size exclusion chromatograms of polymer samples produced in the *ex-situ* kinetic study of the ROP of *rac*-LA at 80°C in PhCl (50% wt/vol), in the presence of 0.1 mol% **7** and 0.3 mol% 4-MeBnOH.

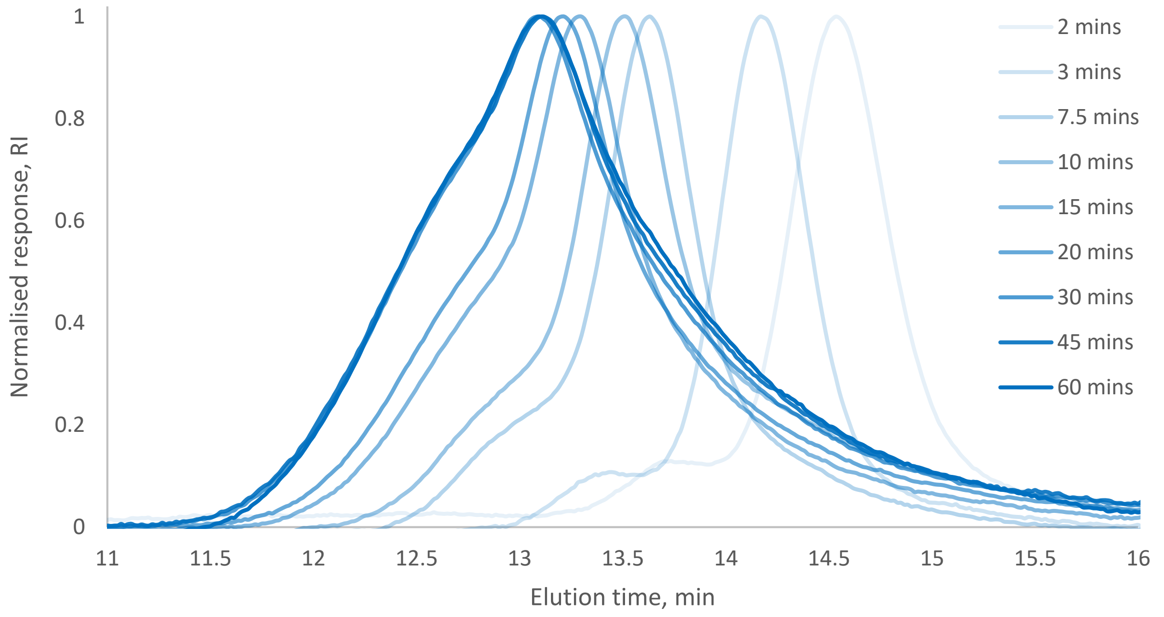

Figure S46. Stacked size exclusion chromatograms of polymer samples produced in the *ex-situ* kinetic study of the ROP of *rac*-LA at 80°C in PhCl (50% wt/vol), in the presence of 0.1 mol% **10** (generated *in situ via* the reduction of **3** in the presence of excess cobaltocene) and 0.3 mol% 4-MeBnOH.

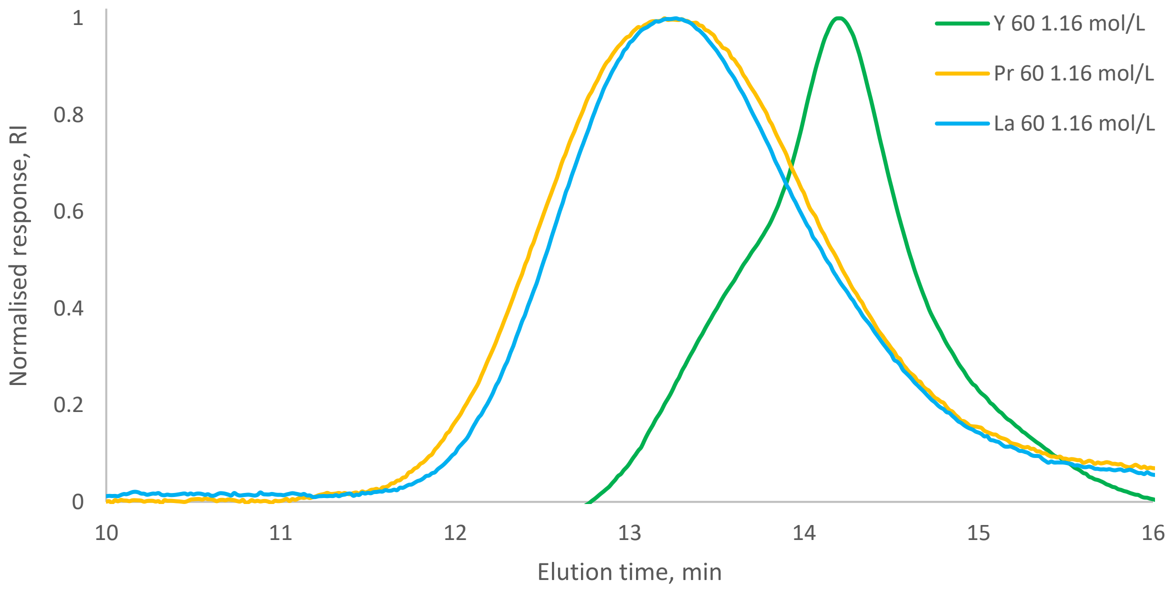

Figure S47. Stacked size exclusion chromatograms of polymer samples produced in the ROP of *rac*-LA at 60°C under very dilute, solution-phase conditions ([LA] = 1.16 mol dm^{-3}) in PhCl, in the presence, variously, of 0.2 mol% of catalyst **5**, **6**, or **7**, and 0.6 mol% 4-MeBnOH.

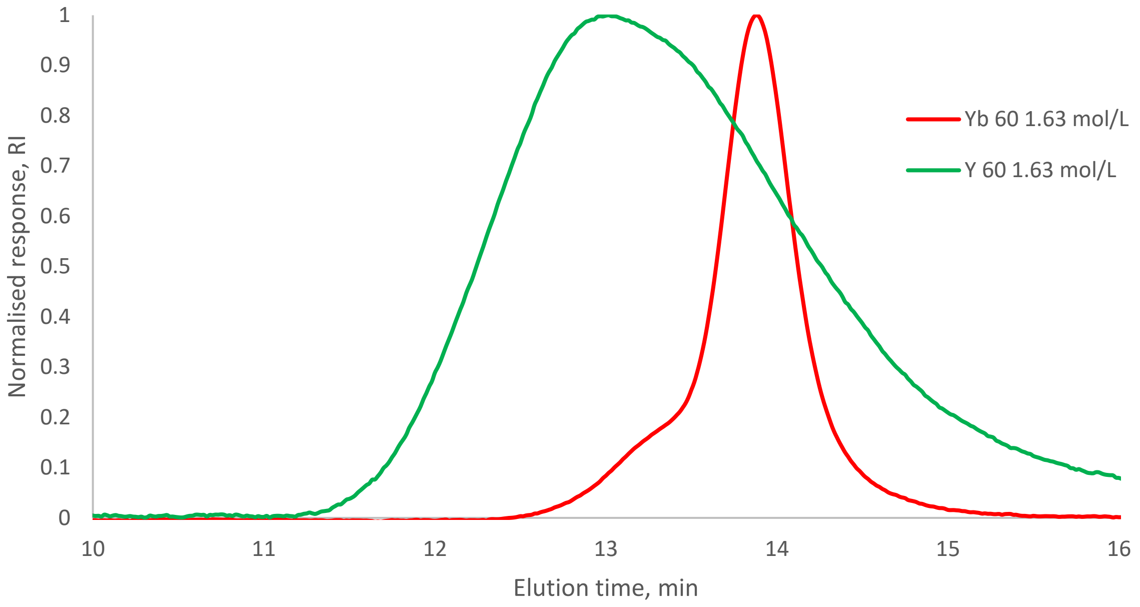

Figure S48. Stacked size exclusion chromatograms of polymer samples produced in the ROP of *rac*-LA at 60˚C under dilute, solution-phase conditions ([LA] = 1.63 mol dm^{-3}) in PhCl, in the presence, variously, of 0.2 mol% of catalyst **4** or **5**, and 0.6 mol% 4-MeBnOH.

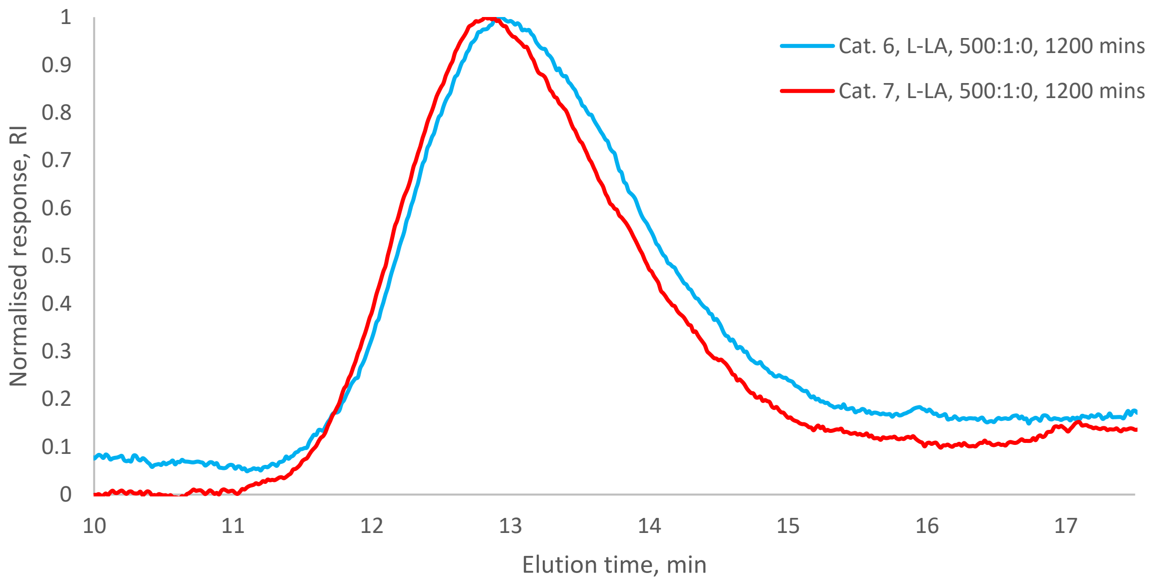

Figure S49. Stacked size exclusion chromatograms of polymer samples produced in the ROP of *L*-LA at 120˚C in PhCl (50% wt/vol), in the presence, variously, of 0.2 mol% of catalyst **6** or **7**, and in the absence of 0.6 mol% 4-MeBnOH, under conditions anticipated to promote the proliferation of side-reactions.

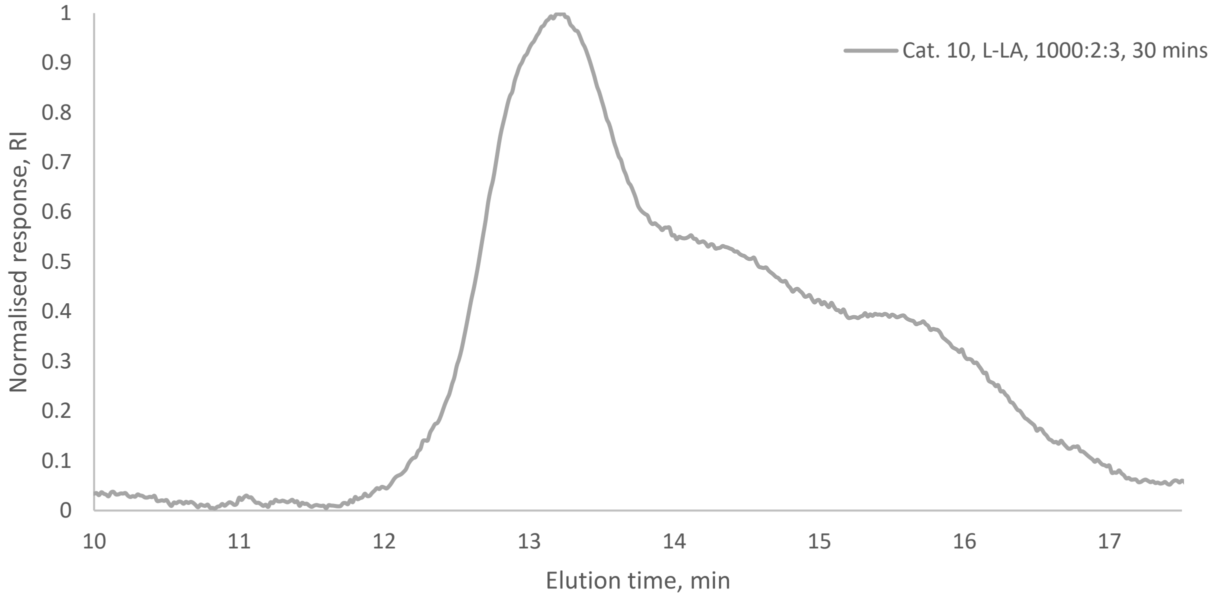

Figure S50. Size exclusion chromatogram of polymer sample produced in the ROP of *L*-LA 120˚C in PhCl (50% wt/vol), in the presence of 0.2 mol% **10** (generated *in situ via* the reduction of **3** in the presence of excess cobaltocene) and 0.3 mol% 4-MeBnOH.

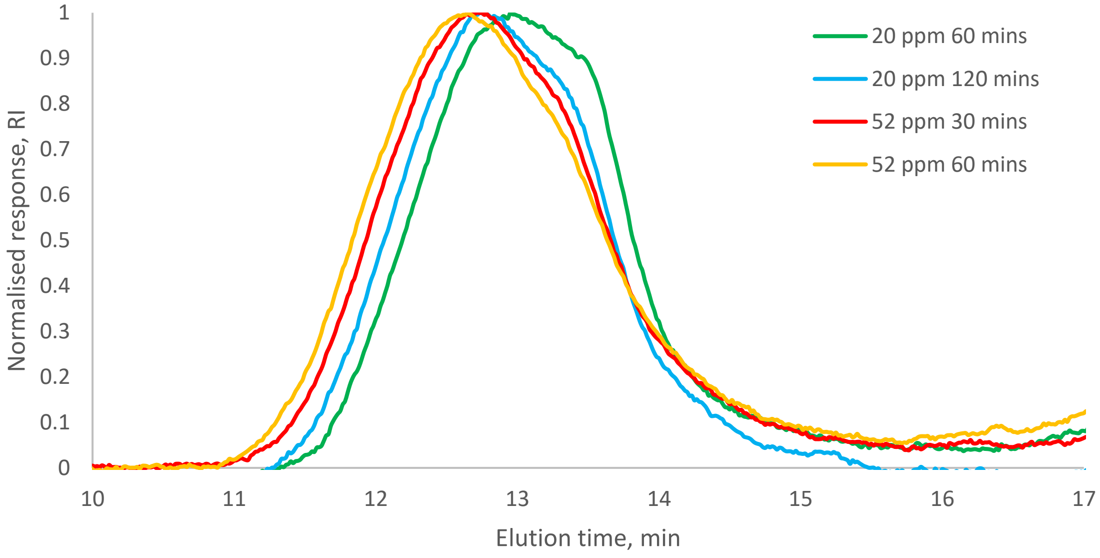

Figure S51. Stacked size exclusion chromatograms of polymer samples produced in the ROP of *L*-LA at 180˚C under solvent-free conditions, in the presence, variously, of $5.35x10^{-3}$ mol% and $2.07x10^{-3}$ mol% **7** (20 ppm and 52 ppm La by weight) and 0.21 mol% 4-MeBnOH, after various reaction durations.

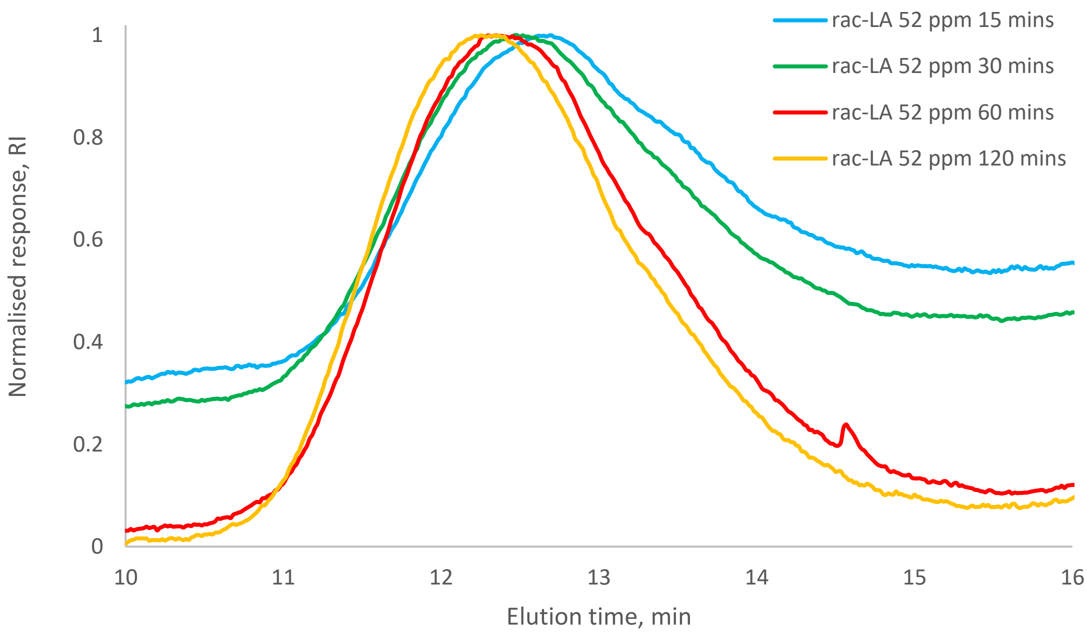

Figure S52. Stacked size exclusion chromatograms of polymer samples produced in the ROP of *rac*-LA at 180˚C under solvent-free conditions, in the presence of 5.35×10^{-3} mol% **7** (52 ppm La by weight) and 0.21 mol% 4-MeBnOH, after various reaction durations.

S9. Sample homonuclear decoupled ^{1}H NMR spectrum of poly(*D*,*L*-LA)

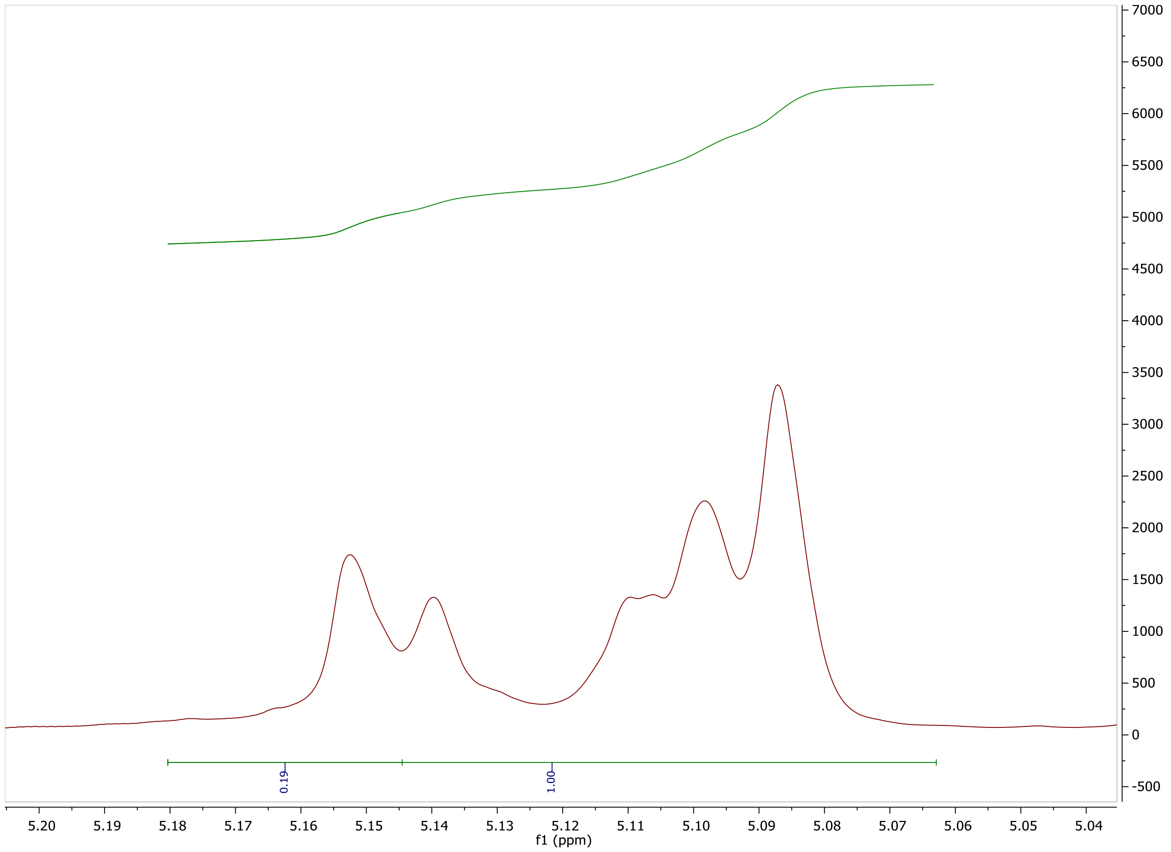

Figure S53. Example ^{1}H NMR spectrum of poly(lactic acid), showing the methine region, wherein homonuclear decoupling from the adjacent methyl protons has been achieved *via* broadband irradiation of the frequency range in which signals corresponding to the latter moiety arise. The integrations represent the method used to determine the abundance of [*sis*] tetrads, from which the tacticity of the polymer can be determined.[11] The specific sample to which this spectrum corresponds is detailed in Entry 5 of Table S11 (P_r = 0.62).

S10. Crystallographic parameters

All solid-phase structures reported in the current work can be found in the Cambridge Structural Database, using Deposition Numbers 2419481-2419493, as detailed in Table S12.

Table S12. CSD deposition numbers for all solid-phase structures of compounds **2** – **10**.

Deposition Number	Compound	Solvent of crystallisation
2419481	**2**	PhMe
2419482	**3**	PhMe
2419483	**4**	$CDCl_3$
2419484	**4**	PhCl
2419485	**5**	$CDCl_3$
2419486	**5**	PhMe, PhCl
2419487	**6**	$CDCl_3$
2419488	**6**	PhMe
2419489	**7**	$CDCl_3$
2419490	**7**	PhMe
2419491	**8**	PhMe
2419492	**9**	PhMe
2419493	**10**	PhCl

Table S13. Crystallographic parameters of all solid-phase structures of compounds **2** – **5**

**Compound reference**	**2**	**3**	**4** (CDCl$_3$)	**4** (PhCl)	**5** (CDCl$_3$)	**5** (PhMe, PhCl)
**Chemical formula**	$C_{54}H_{60}HfN_2O_6$•3(C_7H_8)	$C_{54}H_{62}CeN_2O_6$•3(C_7H_8)	$C_{54}H_{63}N_2O_6Yb$•$CHCl_3$	$C_{54}H_{63}N_2O_6Yb$•2(C_6H_5Cl)	$C_{54}H_{63}N_2O_6Y$•$CHCl_3$	$C_{54}H_{60}N_2O_6Y$•1.21(C_6H_5Cl)•$_0$.79(C_7H_8)
**Formula Mass**	1289.94	1251.57	1128.47	1234.20	1044.34	1130.92
**Crystal system**	Triclinic	Triclinic	Triclinic	Monoclinic	Triclinic	Monoclinic
***a*/Å**	11.2901(3)	11.4795(7)	11.33885(19)	14.36420(10)	11.38802(19)	14.3455(2)
***b*/Å**	11.7290(3)	11.7646(6)	11.86402(17)	24.25915(16)	11.8773(2)	24.4207(3)
***c*/Å**	12.8637(3)	12.7458(6)	20.9291(3)	16.74414(13)	20.9168(3)	16.7365(3)
***α*/°**	81.975(2)	85.911(4)	74.4923(12)	90	74.5949(14)	90
***β*/°**	83.690(2)	84.709(5)	80.3293(13)	93.4523(7)	80.3463(13)	92.6195(15)
***γ*/°**	73.719(2)	71.939(5)	72.8217(14)	90	72.4730(15)	90
**Unit cell volume/Å^3**	1614.62(7)	1627.89(16)	2579.80(7)	5824.13(7)	2589.05(8)	5857.12(15)
**Temperature/K**	150(2)	150(2)	150(2)	150(2)	150(2)	150(2)
**Space group**	$P\bar{1}$	$P\bar{1}$	$P\bar{1}$	*Cc*	$P\bar{1}$	*Cc*
**No. of formula units per unit cell, *Z***	1	1	2	4	2	4
**Radiation type**	Cu Kα	Cu Kα	Cu Kα	Cu Kα	Cu Kα	Cu Kα
**Absorption coefficient, μ/mm^{-1}**	3.404	5.815	5.185	4.233	3.413	2.327
**No. of reflections measured**	17539	10519	39554	60228	36926	54910
**No. of independent reflections**	6418	5951	10758	11030	10953	10248
**R_{int}**	0.0310	0.0357	0.0535	0.0418	0.0298	0.0520
**Final R_1 values ($I > 2\sigma(I)$)**	0.0272	0.0364	0.0460	0.0324	0.0358	0.0678
**Final $wR(F^2)$ values ($I > 2\sigma(I)$)**	0.0724	0.0964	0.1266	0.0831	0.0995	0.1803
**Final R_1 values (all data)**	0.0272	0.0365	0.0494	0.0328	0.0376	0.0685
**Final $wR(F^2)$ values (all data)**	0.0724	0.0966	0.1296	0.0836	0.1011	0.1811
**Goodness of fit on F^2**	1.057	1.035	1.065	1.079	1.079	1.067

Table S14. Crystallographic parameters of all solid-phase structures of compounds **6** – **10**

**Compound reference**	**6** (CDCl$_3$)	**6** (PhMe)	**7** (CDCl$_3$)	**7** (PhMe)	**8**	**9**	**10**
**Chemical formula**	$C_{54}H_{62}N_2O_6Pr$•$CHCl_3$	$C_{54}H_{60}N_2O_6Pr$•2(C_7H_8)	$C_{54}H_{63}LaN_2O_6$•$CHCl_3$	$C_{54}H_{62}LaN_2O_6$•2(C_7H_8)	$C_{54}H_{62}N_2O_6Sc$•5(C_7H_8)	$C_{54}H_{62}N_2O_6Sm$•2(C_7H_8)	$C_{54}H_{62}CeN_2O_6$•$C_{10}H_{10}Co$•2(C_6H_5Cl)
**Formula Mass**	1095.33	1158.21	1094.34	1158.23	1340.68	1169.67	1389.38
**Crystal system**	Monoclinic	Monoclinic	Monoclinic	Monoclinic	Monoclinic	Monoclinic	Monoclinic
***a*/Å**	14.3093(2)	14.6306(7)	14.2972(2)	14.0619(11)	21.3697(2)	14.2030(4)	14.3633(2)
***b*/Å**	23.9865(3)	24.2935(11)	23.9845(4)	25.1538(17)	15.75210(10)	25.2920(7)	11.1731(2)
***c*/Å**	16.6332(2)	16.6001(5)	16.6479(2)	16.6656(10)	22.9250(2)	16.5970(3)	41.3493(7)
***α*/°**	90	90	90	90	90	90	90
***β*/°**	91.4240(15)	93.149(4)	91.5368(13)	92.034(6)	98.8403(4)	92.3130(10)	94.0720(16)
***γ*/°**	90	90	90	90	90	90	90
**Unit cell volume/Å^3**	5707.25(13)	5891.2(4)	5706.70(14)	5891.1(7)	7625.29(11)	5957.2(3)	6619.09(19)
**Temperature/K**	150(2)	150(2)	150(2)	150(2)	150(2)	200(2)	150(2)
**Space group**	*Cc*	*C*2/*c*	*Cc*	$P2_1/c$	$P2_1$	$P2_1/c$	$P2_1/c$
**No. of formula units per unit cell, *Z***	4	4	4	4	4	4	4
**Radiation type**	Cu Kα	Cu Kα	Mo Kα	Mo Kα	MoKα	MoKα	Cu Kα
**Absorption coefficient, μ/mm^{-1}**	8.205	6.758	0.935	0.778	0.151	1.038	8.378
**No. of reflections measured**	50433	26661	45085	26315	136282	90332	108143
**No. of independent reflections**	10987	5861	13779	26315	34224	10458	13217
**R_{int}**	0.0539	0.0893	0.0399	-	0.0762	0.1356	0.0739
**Final R_1 values ($I > 2\sigma(I)$)**	0.0449	0.0940	0.0289	0.0608	0.0649	0.0513	0.0578
**Final $wR(F^2)$ values ($I > 2\sigma(I)$)**	0.1198	0.2231	0.0624	0.1214	0.1456	0.0903	0.1408
**Final R_1 values (all data)**	0.0463	0.1035	0.0314	0.0872	0.1134	0.0971	0.0709
**Final $wR(F^2)$ values (all data)**	0.1210	0.2285	0.0634	0.1272	0.1685	0.1026	0.1493
**Goodness of fit on F^2**	1.111	1.172	1.034	1.047	1.025	1.081	1.035

S11. Additional crystal structure figures

Selected representations of solid-phase structures, additional to those appearing in the main text, are included below. A complete record of all structures discussed in this work, including those corresponding to compound conformations for which Figures have not been included in the main text or the Supporting Information can be accessed *via* the Cambridge Structural Database, using the deposition numbers provided in Table S12.

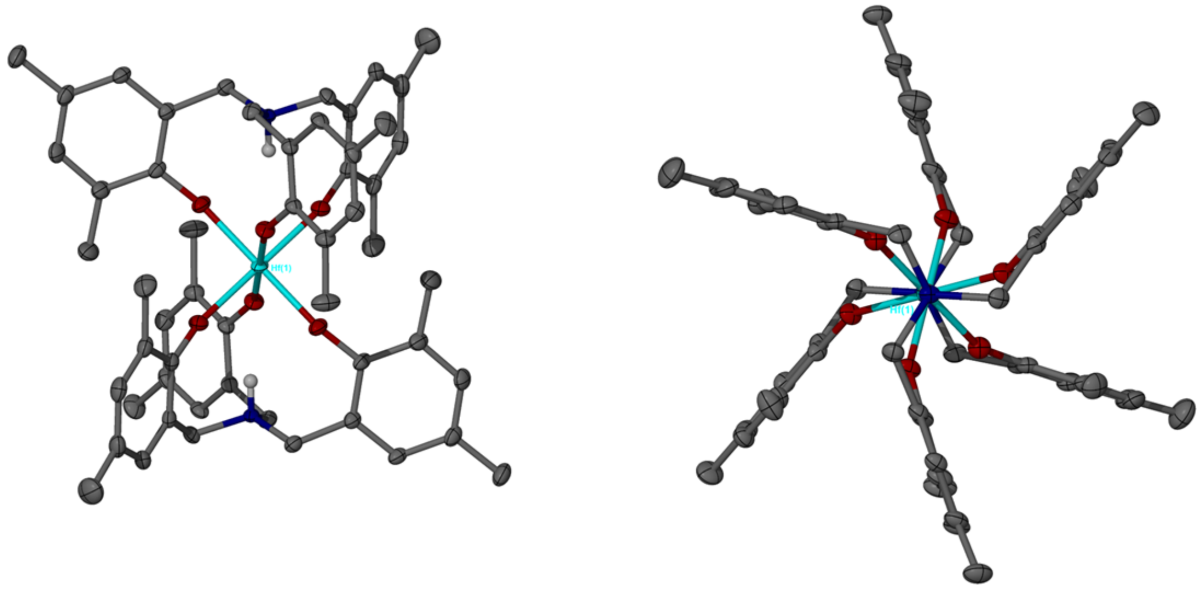

Figure S54. Solid-phase structure of Hf(IV) compound **2**, including N-M-N axial view, showing the heteroditopic ligand conformation and staggered M-O bonds. Selected structural parameters are provided in Table 1 in the main text. Ellipsoids are drawn at the 50% probability level. All solvent molecules, carbon-bonded hydrogen atoms and intramolecular N-H···O interactions have been omitted for clarity.

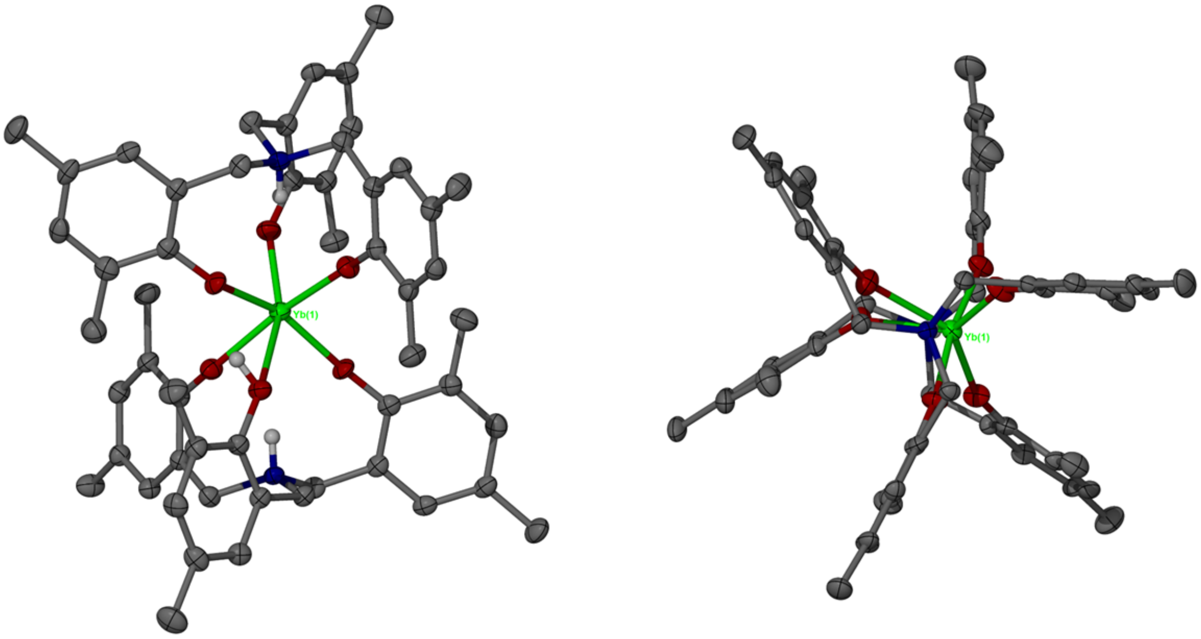

Figure S55. Solid-phase structure of the homoditopic form of Yb(III) compound **4**, including N-M-N axial view, showing the ligand conformation and eclipsed M-O bonds. Selected structural parameters are provided in Table 1 in the main text. Ellipsoids are drawn at the 50% probability level. All solvent molecules, carbon-bonded hydrogen atoms and intramolecular N-H···O interactions have been omitted for clarity.

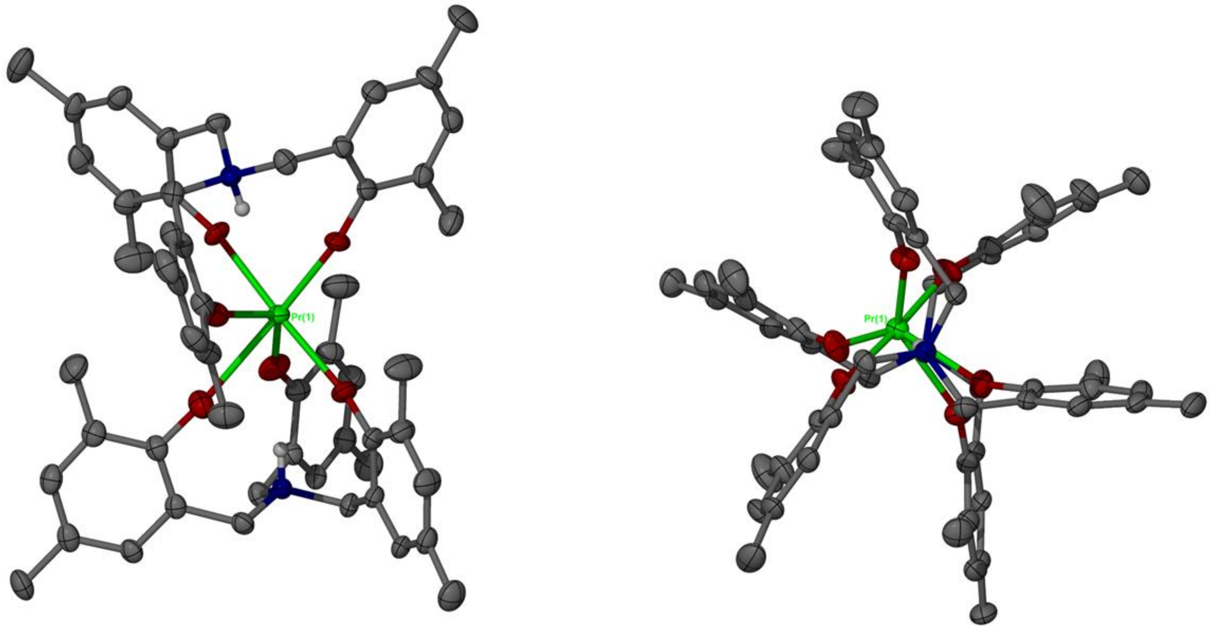

Figure S56. Solid-phase structure of a homoditopic form of Pr(III) compound **6**, including N-M-N axial view, showing the ligand conformation and eclipsed M-O bonds. Selected structural parameters are provided in Table 1 in the main text. Ellipsoids are drawn at the 50% probability level. All solvent molecules, carbon-bonded hydrogen atoms and intramolecular N-H···O interactions have been omitted for clarity.

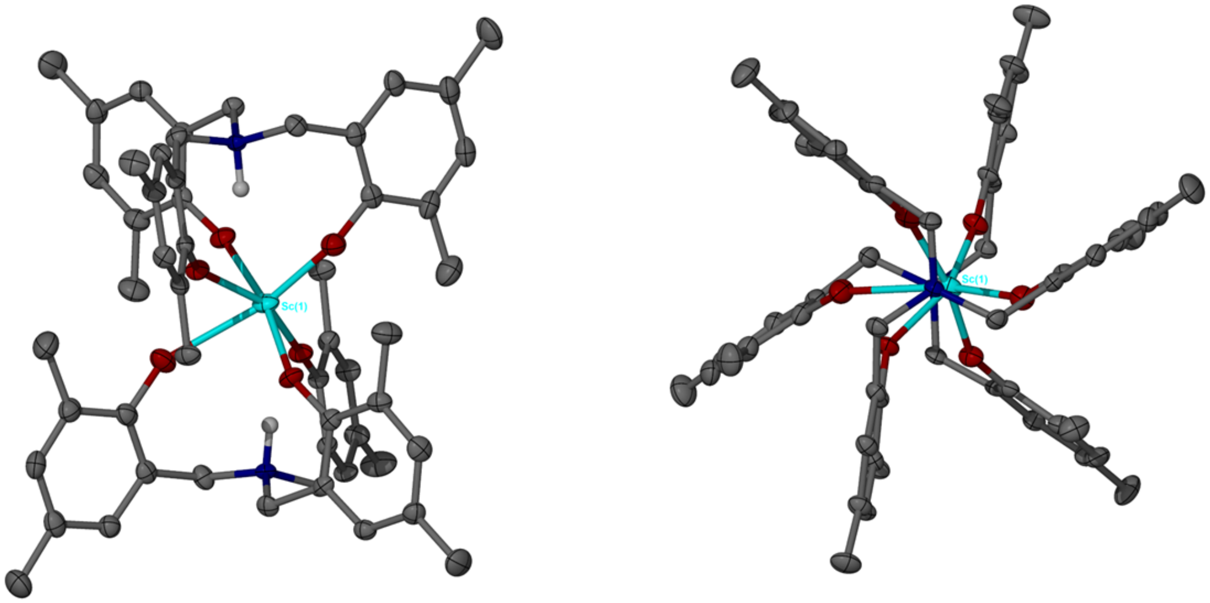

Figure S57. Solid-phase structure of Sc(III) compound **8**, including N-M-N axial view, showing the heteroditopic ligand conformation and staggered M-O bonds. Selected structural parameters are provided in Table 1 in the main text. Ellipsoids are drawn at the 50% probability level. All solvent molecules, carbon-bonded hydrogen atoms and intramolecular N-H···O interactions have been omitted for clarity.

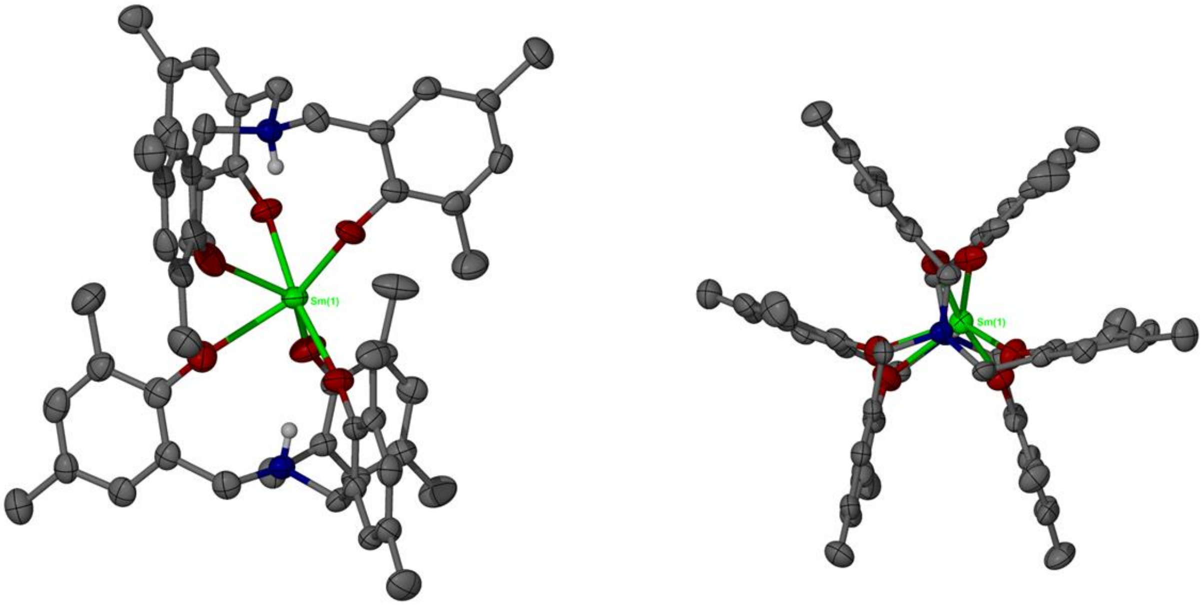

Figure S58. Solid-phase structure of Sm(III) compound **9**, including N-M-N axial view, showing the homoditopic ligand conformation and eclipsed M-O bonds. Selected structural parameters are provided in Table 1 in the main text. Ellipsoids are drawn at the 50% probability level. All solvent molecules, carbon-bonded hydrogen atoms and intramolecular N-H···O interactions have been omitted for clarity.

References

(1) Otwinowski, Z.; Minor, W. [20] Processing of X-Ray Diffraction Data Collected in Oscillation Mode. *Methods Enzymol.* **1997**, *276*, 307–326. https://doi.org/10.1016/S0076-6879(97)76066-X.

(2) Sheldrick, G. M. Crystal Structure Refinement with SHELXL. *Acta Crystallogr. Sect. C Struct. Chem.* **2015**, *71* (1), 3–8. https://doi.org/10.1107/S2053229614024218.

(3) Hübschle, C. B.; Sheldrick, G. M.; Dittrich, B. ShelXle: A Qt Graphical User Interface for SHELXL. *J. Appl. Crystallogr.* **2011**, *44* (6), 1281–1284. https://doi.org/10.1107/S0021889811043202.

(4) Macrae, C. F.; Edgington, P. R.; McCabe, P.; Pidcock, E.; Shields, G. P.; Taylor, R.; Towler, M.; Van De Streek, J. Mercury: Visualization and Analysis of Crystal Structures. *J. Appl. Crystallogr.* **2006**, *39* (3), 453–457. https://doi.org/10.1107/S002188980600731X.

(5) Kol, M.; Shamis, M.; Goldberg, I.; Goldschmidt, Z.; Alfi, S.; Hayut-Salant, E. Titanium(IV) Complexes of Trianionic Amine Triphenolate Ligands. *Inorg. Chem. Commun.* **2001**, *4* (4), 177–179. https://doi.org/10.1016/S1387-7003(01)00157-5.

(6) Buchard, A.; Davidson, M. G.; Gobius du Sart, G.; Jones, M. D.; Kociok-Köhn, G.; McCormick, S. N.; Mckeown, P. Coordination of ε-Caprolactone to a Cationic Niobium(V) Alkoxide Complex: Fundamental Insight into Ring-Opening Polymerization via Coordination–Insertion. *Inorg. Chem.* **2023**, *62* (38), 15688–15699. https://doi.org/10.1021/acs.inorgchem.3c02491.

(7) Buchard, A.; Chuck, C. J.; Davidson, M. G.; Gobius du Sart, G.; Jones, M. D.; McCormick, S. N.; Russell, A. D. A Highly Active and Selective Zirconium-Based Catalyst System for the Industrial Production of Poly(Lactic Acid). *ACS Catal.* **2023**, 2681–2695. https://doi.org/10.1021/ACSCATAL.2C05690.

(8) Davidson, M. G.; Doherty, C. L.; Johnson, A. L.; Mahon, M. F. Isolation and Characterisation of

Transition and Main Group Metal Complexes Supported by Hydrogen-Bonded Zwitterionic Polyphenolic Ligands. *Chem. Commun.* **2003**, No. 15, 1832. https://doi.org/10.1039/b303618a.

(9) Elgrishi, N.; Rountree, K. J.; McCarthy, B. D.; Rountree, E. S.; Eisenhart, T. T.; Dempsey, J. L. A Practical Beginner's Guide to Cyclic Voltammetry. *J. Chem. Educ.* **2018**, *95* (2), 197–206. https://doi.org/10.1021/ACS.JCHEMED.7B00361/SUPPL_FILE/ED7B00361_SI_002.DOCX.

(10) Baer, A.; Wawra, S. E.; Bielmeier, K.; Uttinger, M. J.; Smith, D. M.; Peukert, W.; Walter, J.; Smith, A. S. The Stokes–Einstein–Sutherland Equation at the Nanoscale Revisited. *Small* **2024**, *20* (6), 2304670. https://doi.org/10.1002/SMLL.202304670.

(11) Cheng, M.; Attygalle, A. B.; Lobkovsky, E. B.; Coates, G. W. Single-Site Catalysts for Ring-Opening Polymerization: Synthesis of Heterotactic Poly(Lactic Acid) from Rac-Lactide [9]. *J. Am. Chem. Soc.* **1999**, *121* (49), 11583–11584. https://doi.org/10.1021/ja992678o.

(12) Shannon, R. D. Revised Effective Ionic Radii and Systematic Studies of Interatomic Distances in Halides and Chalcogenides. *urn:issn:0567-7394* **1976**, *32* (5), 751–767. https://doi.org/10.1107/S0567739476001551.
